# Supplementary figures and images for: CHOmics: A web-based tool for multi-omics data analysis and interactive visualization in CHO cell lines
Source: PLoS Comput Biol. 2020 Dec 22;16(12):e1008498. doi: 10.1371/journal.pcbi.1008498 (PMC7790544; doi:10.1371/journal.pcbi.1008498)

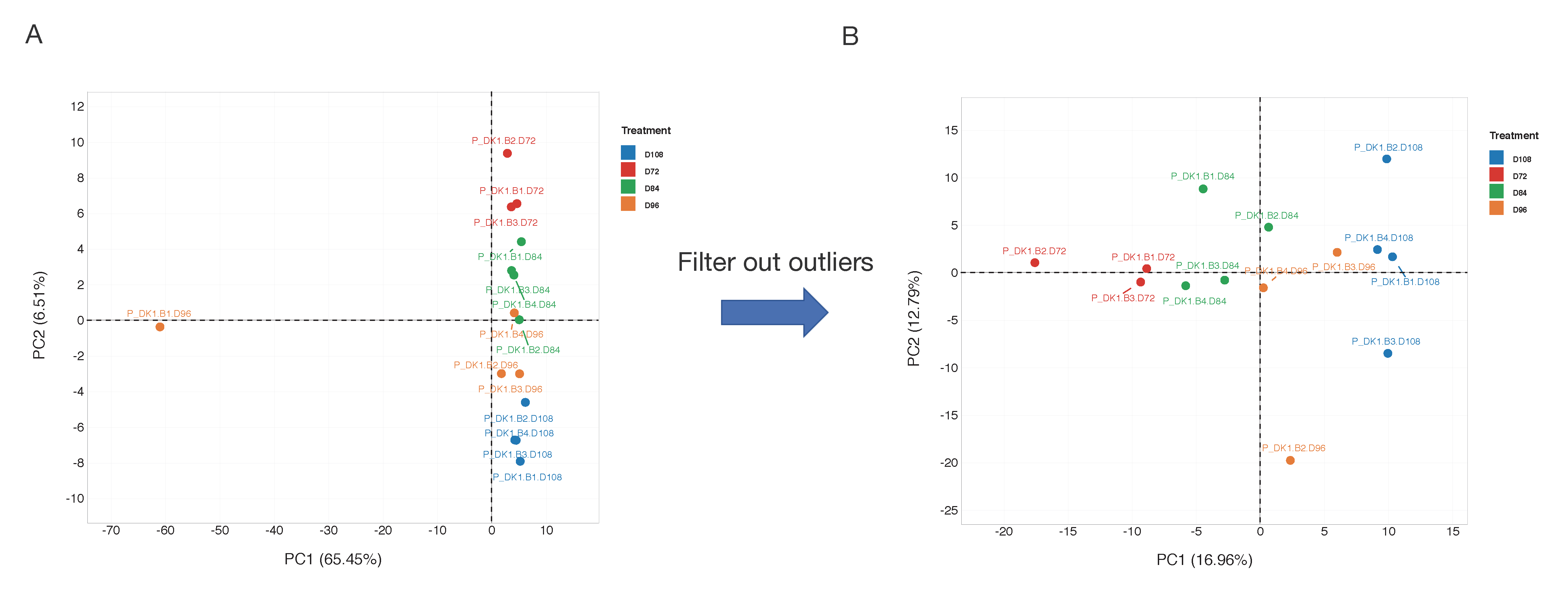

Supplement: S1 Fig — (A) PCA analysis on proteomics data shows that one sample at 96 hr is outlier. (B) The samples are clustered mainly by treatment (i.e., time points) after filtering out the outlier. (TIFF) [file pcbi.1008498.s001.tiff]

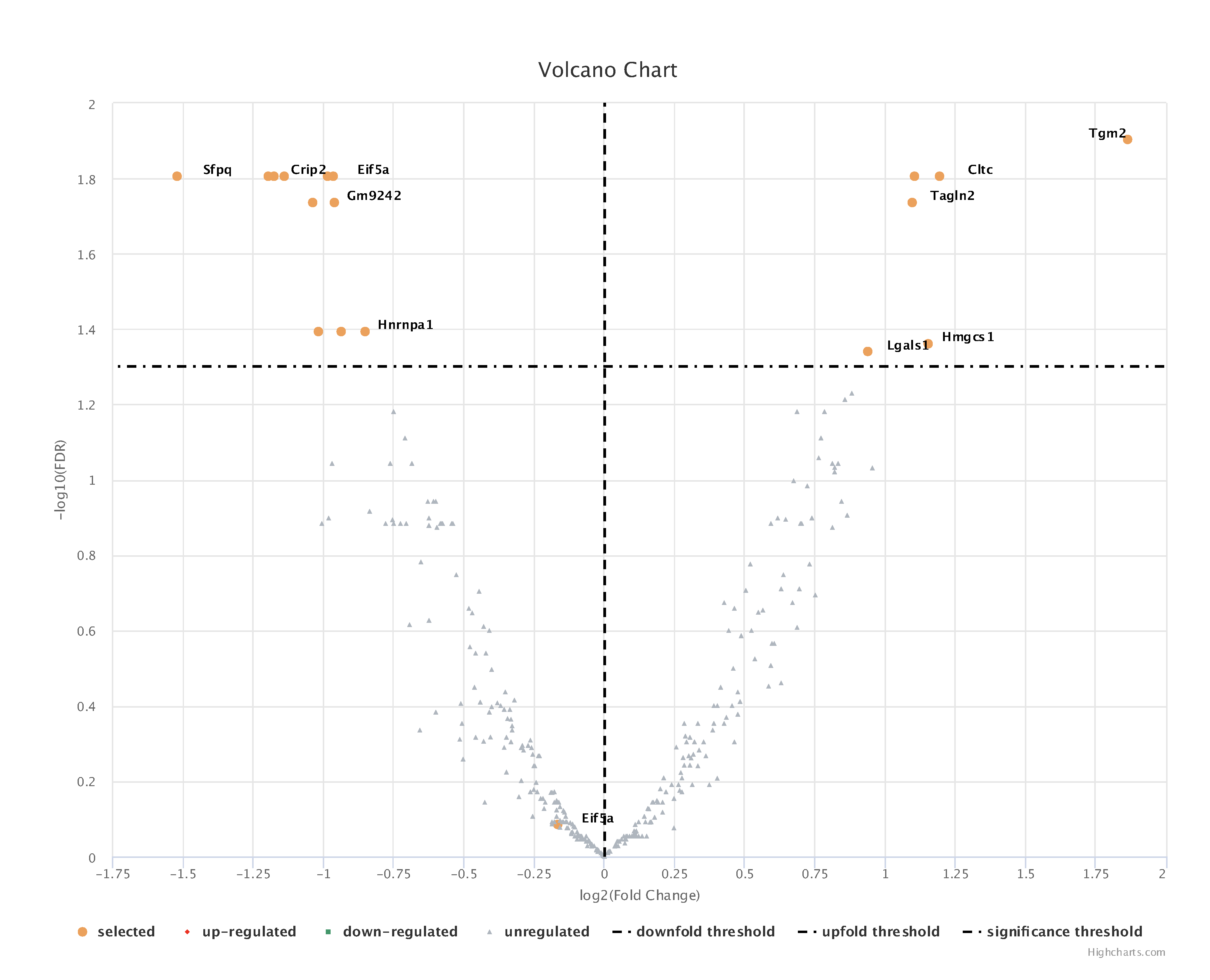

Supplement: S2 Fig — The top differentially expressed proteins between 108 hr and 72 hr. (TIFF) [file pcbi.1008498.s002.tiff]

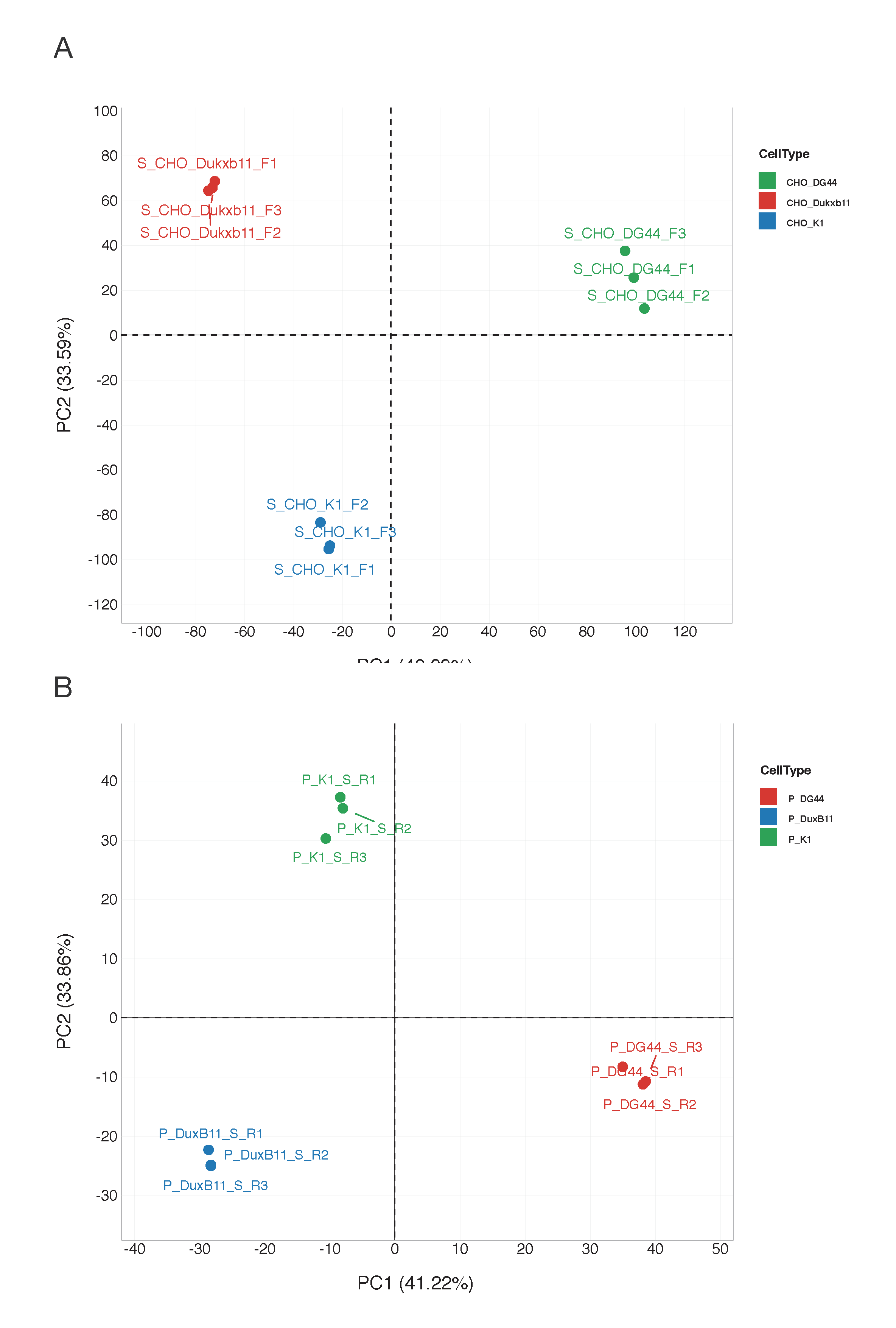

Supplement: S3 Fig — (A) Nine samples from three groups (CHO_DG44, CHO_Dukxb11, and CHO_K1) were clustered based on the first and second PCs of transcriptomics data. (B) The samples were clustered based on the first and second PCs of proteomics data. (TIFF) [file pcbi.1008498.s003.tiff]

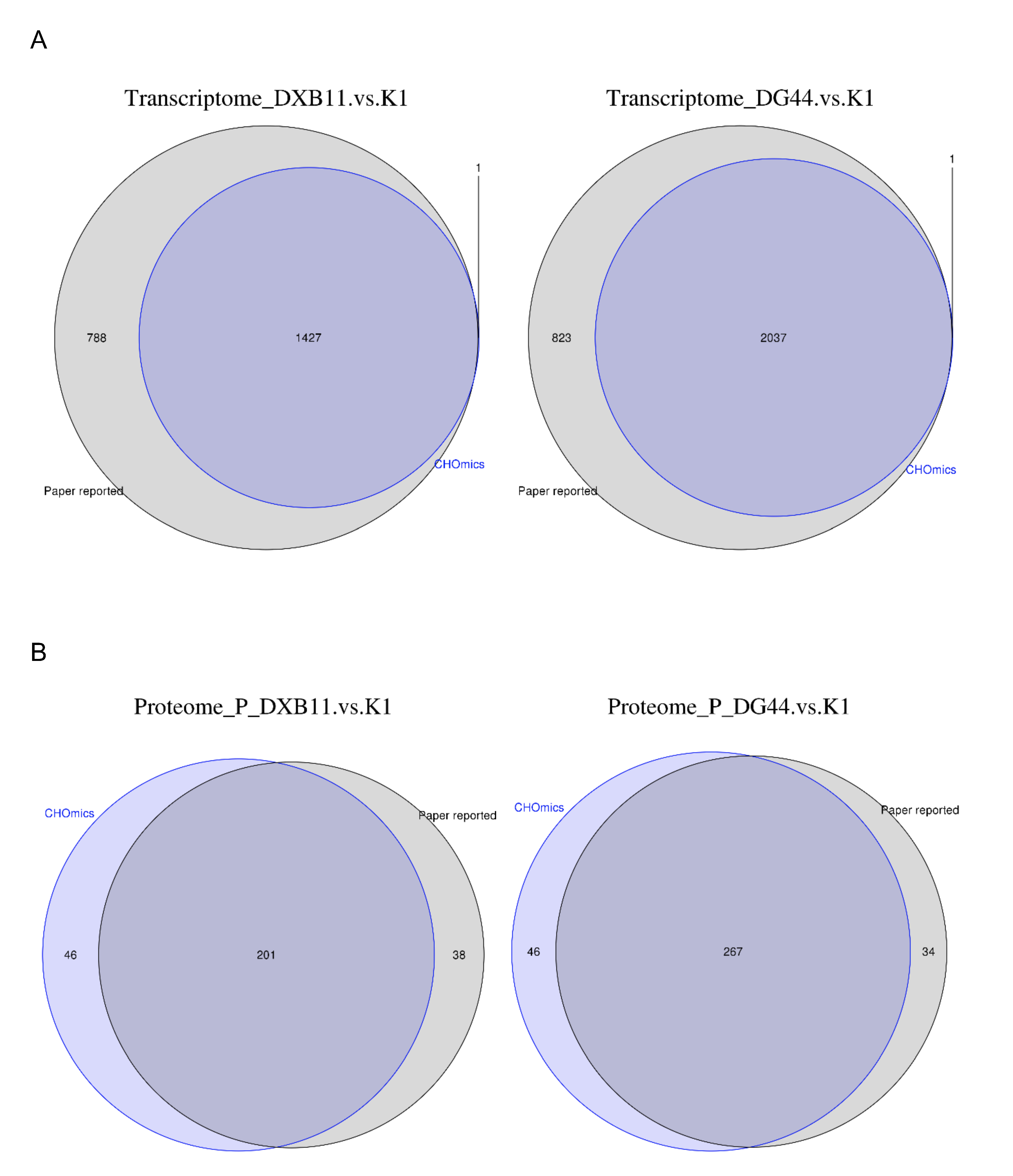

Supplement: S4 Fig — (TIFF) [file pcbi.1008498.s004.tiff]

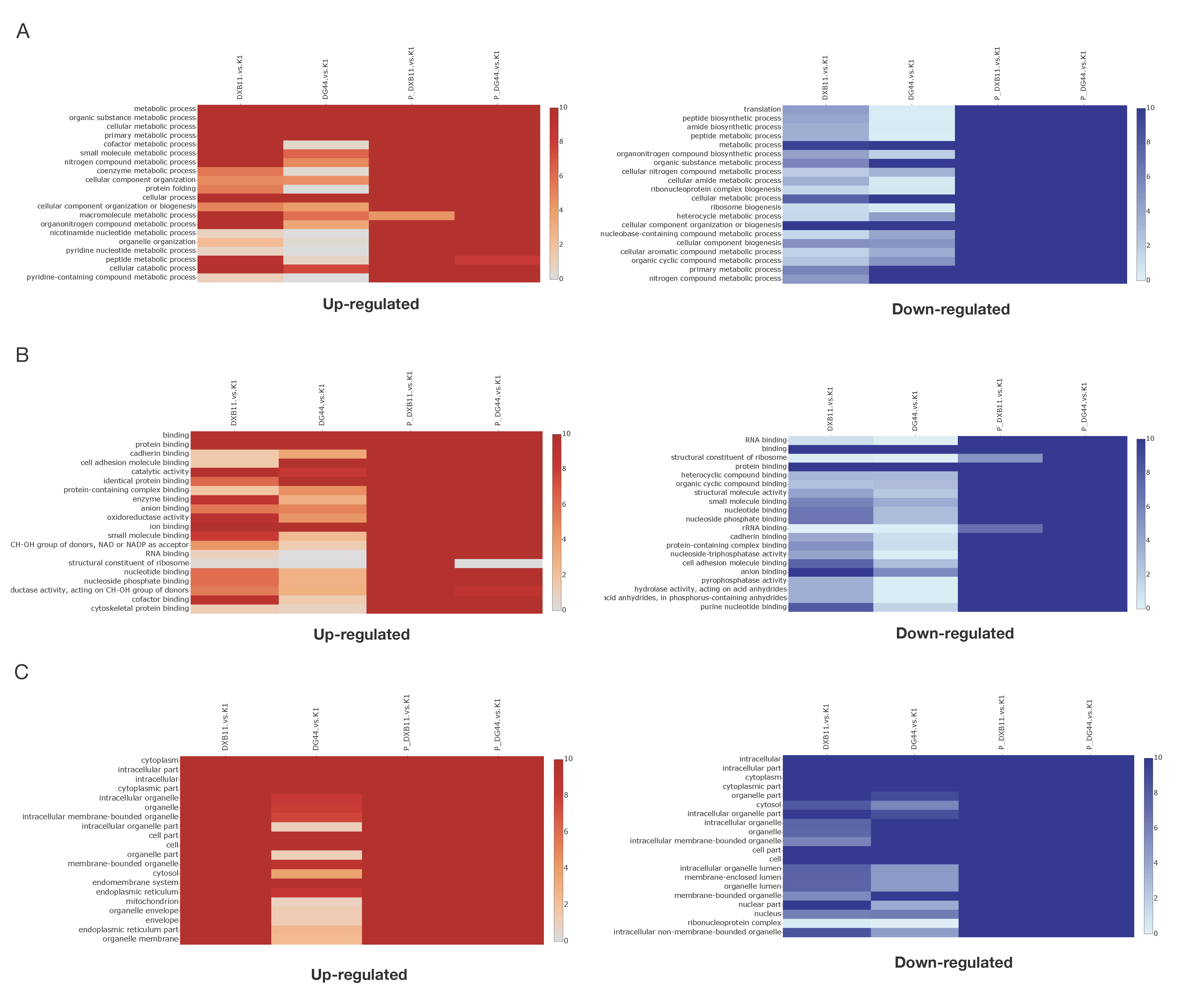

Supplement: S5 Fig — The enrichment analysis on (A) biological processes, (B) molecular functions, and (C) cellular components of GO. (TIFF) [file pcbi.1008498.s005.tiff]

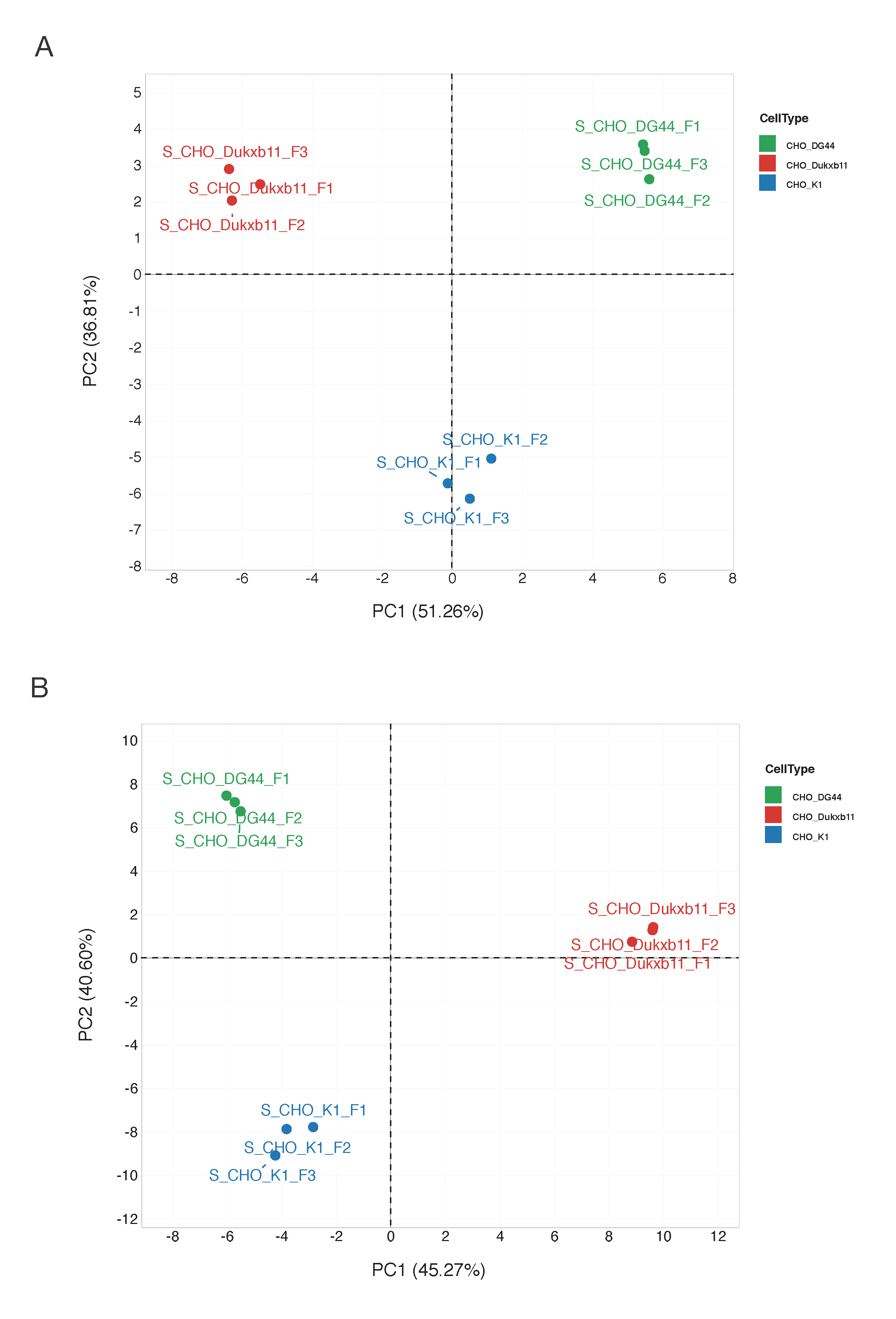

Supplement: S6 Fig — (A) The genes were selected from (A) N-glycan biosynthesis pathway, and (B) oxidative phosphorylation pathway of KEGG. (TIFF) [file pcbi.1008498.s006.tiff]

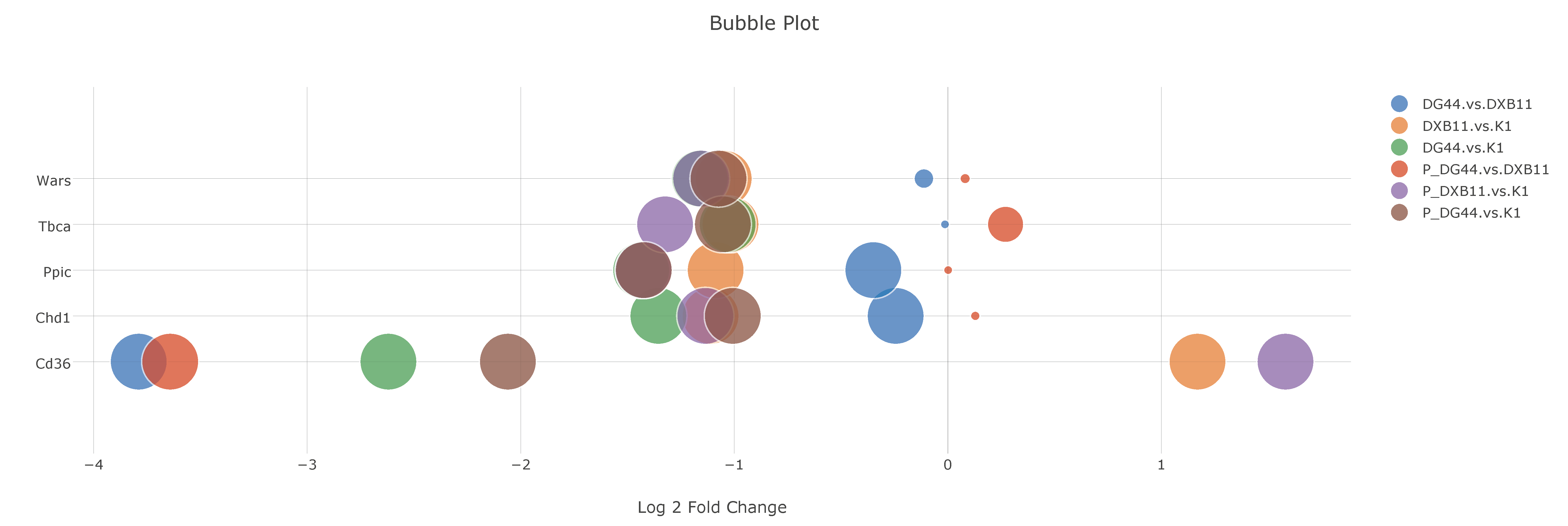

Supplement: S7 Fig — Common differentially expressed genes from comparisons of both transcriptomics and proteomics data analysis are shown. The bubble sizes are proportional to significance levels (-logFDR) of differentially expressed genes in various comparisons that are color-coded. (TIFF) [file pcbi.1008498.s007.tiff]

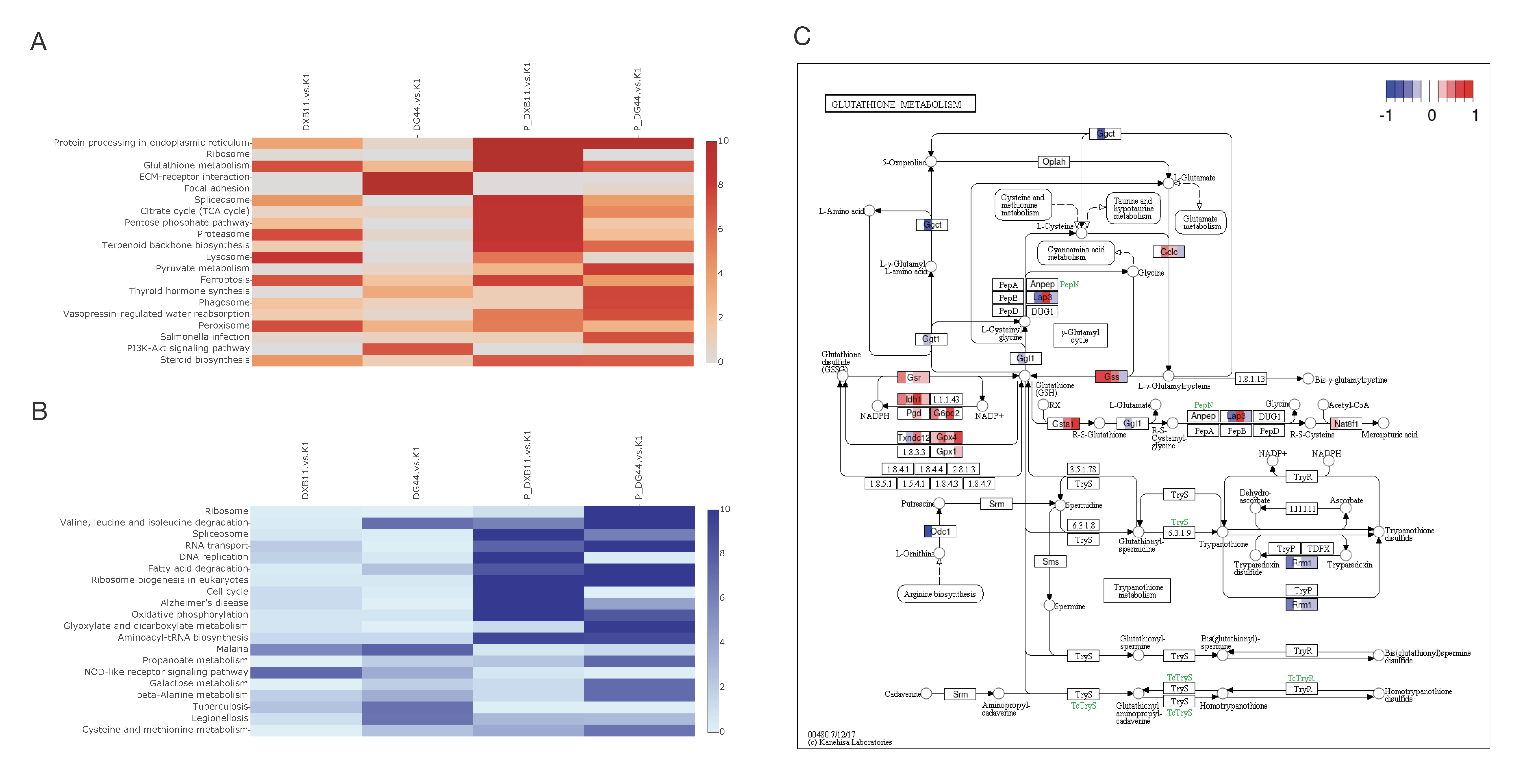

Supplement: S8 Fig — (A) Top 20 pathways enriched by up-regulated differentially expressed genes from both transcriptomics and proteomics data. (B) Pathways enriched by down-regulated differentially expressed genes. (C) Differential analysis statistics from multi-omics data were aggregated into the pathway diagram of Glutathione metabolism from KEGG database. Each box is divided into equal stripes to show color-coded log2 fold changes capped at 1 where each stripe corresponds to one comparison. (TIFF) [file pcbi.1008498.s008.tiff]
