# Supplementary material for: CHOmics: A web-based tool for multi-omics data analysis and interactive visualization in CHO cell lines
Source: PLoS Comput Biol. 2020 Dec 22;16(12):e1008498. doi: 10.1371/journal.pcbi.1008498 (PMC7790544; doi:10.1371/journal.pcbi.1008498)
Supplement: S1 Text — (PDF) [file pcbi.1008498.s009.pdf]

CHOmics Tutorial

Version 1.0, Feb, 2020

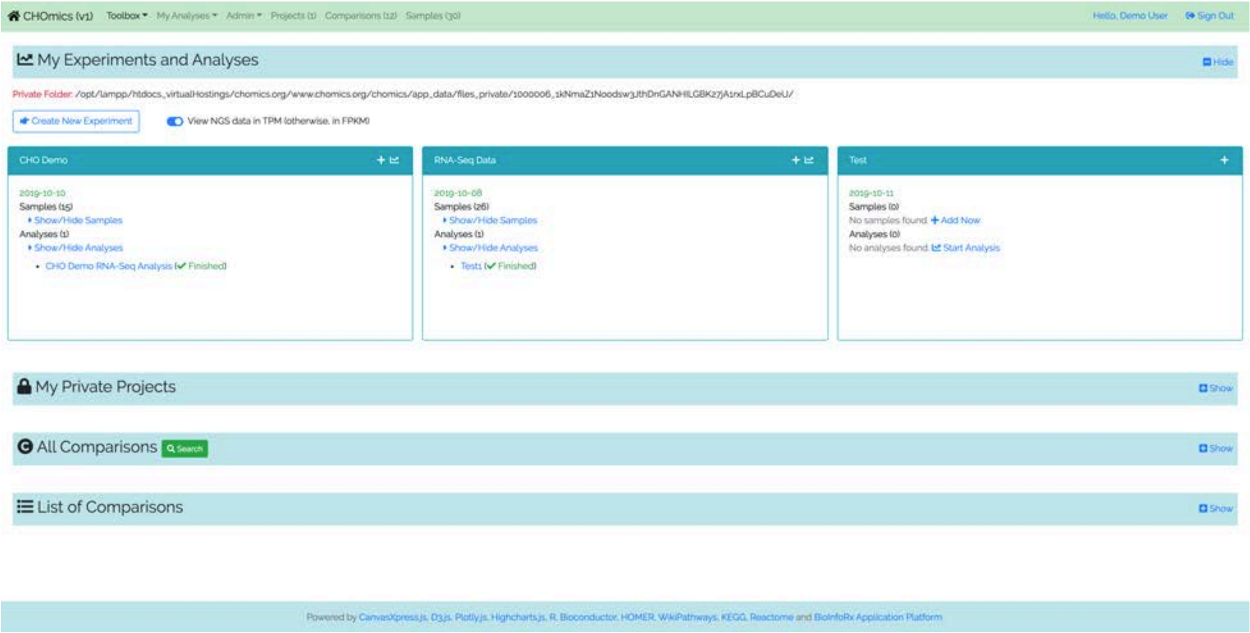

http://chomics.org

user:demo@bioinforx.com

password:CHO_demo

From the login page, you can use your email to register an account that is recommended, as you will be able to save results and upload your own data. Otherwise just use guest account to view public data.

Contents

1. Overview of CHOmics 4
1.1 Menu Bar 4
1.2 Experiments and Analyses 4
1.3 Projects 5
1.4 Samples 5
1.5 Comparisons 6
1.6 Genes 8
2 Data Input 9
2.1 Upload fastq files to experiment 9
2.2 Upload data file to project 10
3 Data Analysis 12
3.1 RNAseq analysis pipeline 12
3.2 DE, GSEA and GO analysis 18
3.3 Saved Genes and Comparisons 21
3.4 Advanced Analysis 23
3.3.1 Correlation Tools 23
3.3.2 PCA Analysis 24
3.3.3 Meta-Analysis 26
4 Visualization 29
4.1 Visualize Gene Expression 29
4.1.1 View Gene Expression from multiple samples 29
4.1.2 View Gene Expression in Heatmap 32
4.1.3 Multi-omics Expression View 33
4.2 Visualize Comparison Data 34
4.2.1 Dashboard View of Comparison 34
4.2.2 Bubble Plot 36
4.2.3 Get significant genes from comparisons 41
4.2.4 Volcano Plot 44
4.3 Visualize functional pathway 46
4.3.1 Enrichment from Up and Down Regulated Genes 46
4.3.2 View Changed Genes from a Functional Term in Volcano Plot 47

4.3.3 View Enriched Pathways Directly from Comparison Details 49
4.3.4 Multi-layer visualization 50
4.3.5 Pathway Heatmap From Comparisons 54
5 Customized analysis pipeline 56
5.1 Use alternative tool or algorithm 56

1. Overview of CHOmics

There are several panels stacked in the main interface. The recent experiment and projects are listed in the panel separately for quick access. You can also access them and other functions from the shortcuts at the top menu bar.

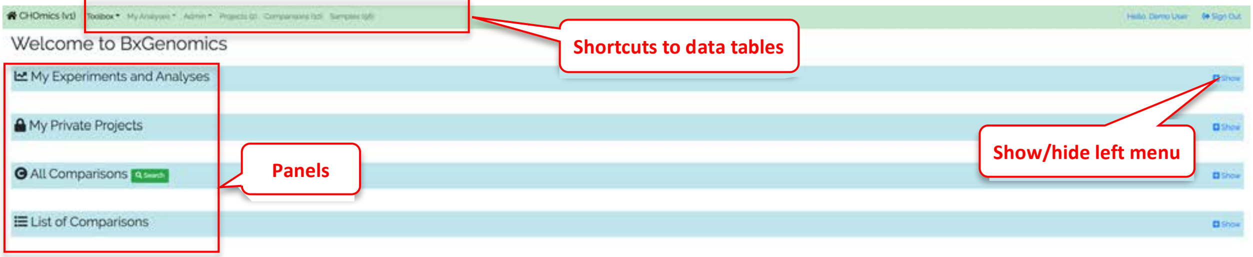

1.1 Menu Bar

In top menu bar, several shortcuts are listed for quick access of functions including: Toolbox, My Analysis, and Admin, Projects, Comparisons and Samples.

'Toolbox' contains a list of functional modules including: 'Import Project Data','Gene Expression Analysis', 'Comparison-based Analysis', 'Pathway Visualization' and 'Other tools'.

'My Analysis' provides quick access to the information of all 'Experiments', 'Samples' and 'Analysis'.

'Admin' allows the users to manage the data files from private folder, shared folder and overview the platforms applied to all data sets.

'Projects', 'Comparisons' and 'Samples' all provide searching function and access to specific project, comparison and sample respectively.

1.2 Experiments and Analyses

Experiment is designed for running the built-in RNA sequencing pipeline on the raw sequencing data. Once the experiment is created, users can upload raw fastq files and sample meta information, and then launch the built-in pipeline for analysis. After the analysis is completed, the analysis report is generated and the results can be exported as one 'Project' for visualization and cross-project comparison.

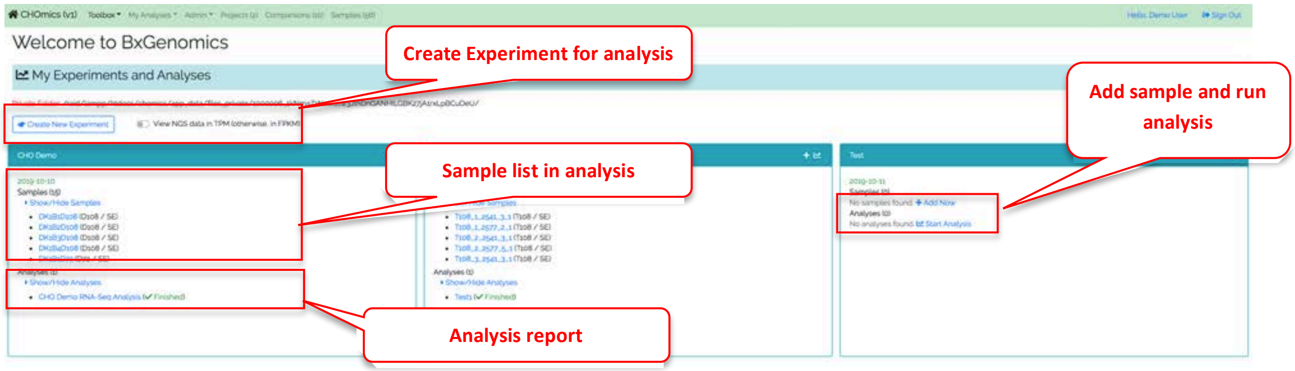

1.3 Projects

The project is used to perform data mining and data visualization. Users can either import analysis report from ‘Experiment’ or upload pre-processed data to create a project. In the project, users can easily explore different features of the data (e.g, Gene expression profiling, sample clustering, PCA, differential expression genes and pathways, etc), compare the analysis with the other projects or perform the meta-analysis by combining multiple projects.

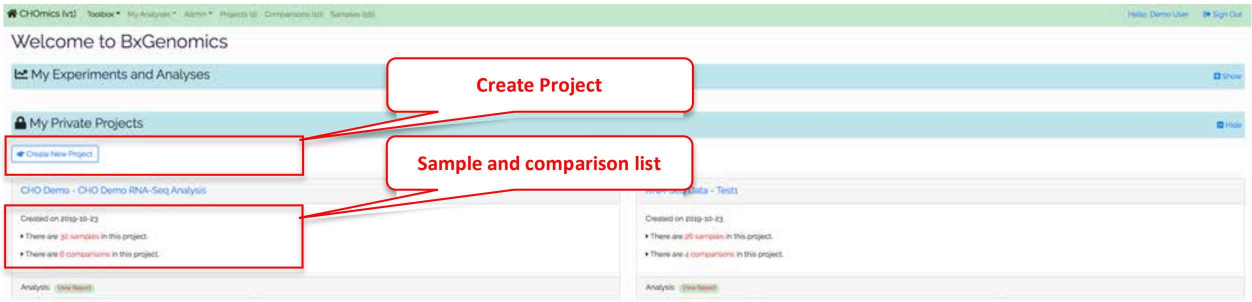

Each project mainly consists of samples including both meta information and omics profiling, and comparisons showing the statistical differences among samples.

1.4 Samples

A project may include many samples which can be searched by the ‘Sample’ in top menu bar. Each sample has its own properties including Species, CellType, DiseaseState,etc (details available by clicking the ☰ button on the left ends).

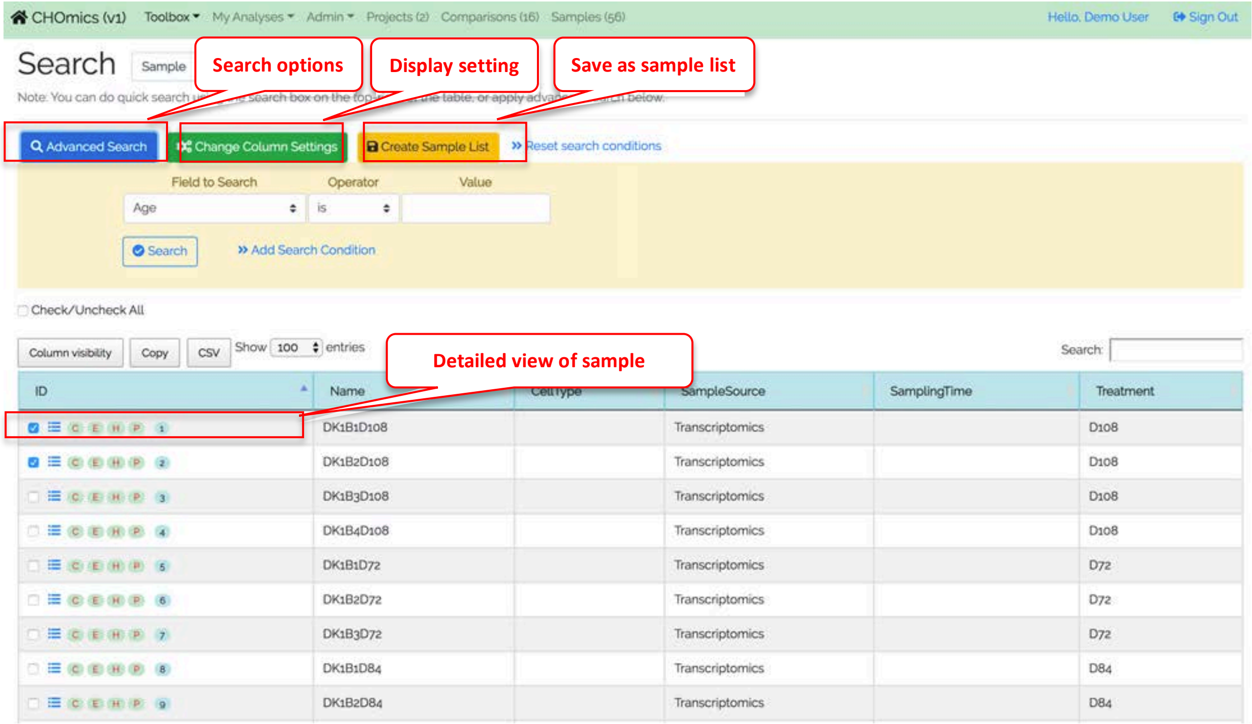

To change columns displayed in the table, using the table settings (green button). Users can also select the samples to save them into the sample list. Samples from the list can be loaded to other analysis or visualization stools like heatmap.

Each sample has a gene expression profile. In CHOmics, there are multiple ways to analyze and visualize the samples including: correlation tool (noted by ‘C’), gene expression plot(noted by ‘E’), expression heatmap (noted by ‘H’), and PCA analysis (noted by ‘P’).

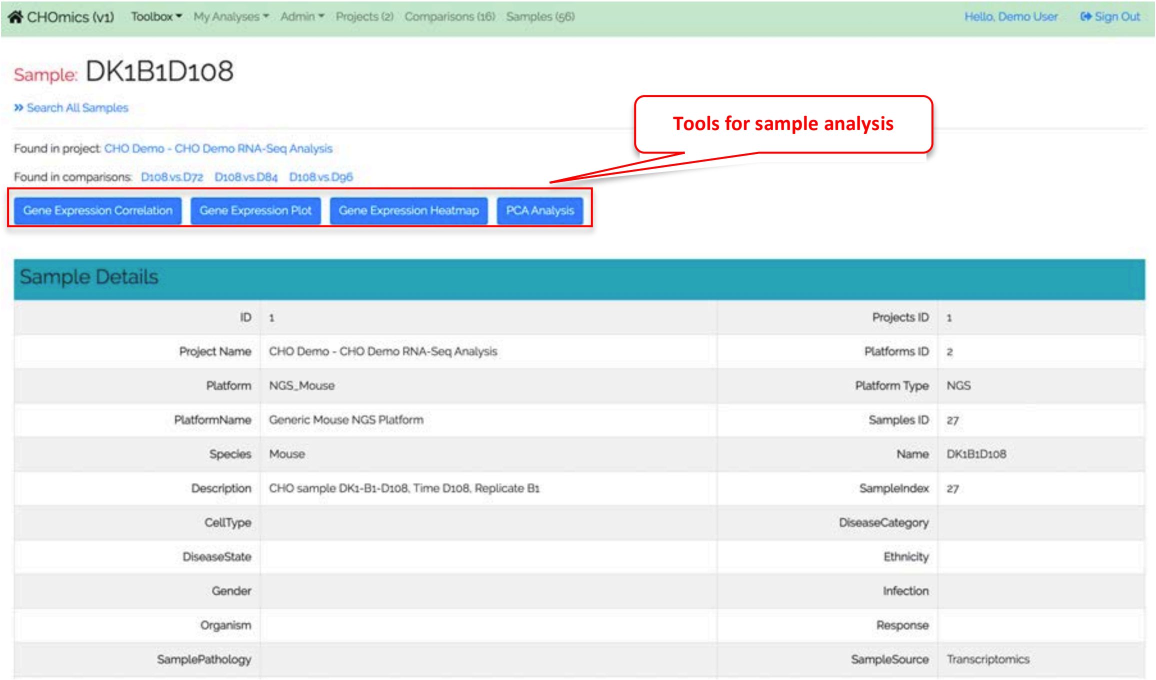

1.5 Comparisons

Comparison is defined by the comparative analysis between two groups of samples including differential gene analysis and pathway enrichment analysis.

There are a lot of meta data available for each comparison. See the dashboard for an overview of key categories, and the detailed description of each comparison has the full information.

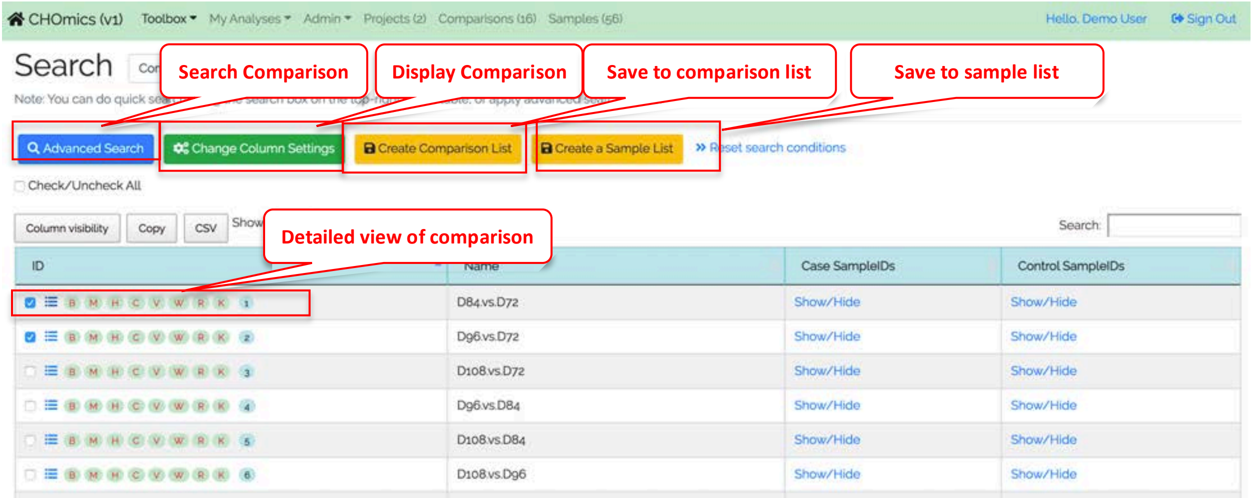

The selected comparisons can be saved to the comparison list (yellow button) for easy loading into the plotting tools.

Several options on each comparison for complicated visualization and analysis are also listed including: bubble plot of gene expressions(noted by ‘B’), meta analysis (noted by ‘M’), pathway heamap plot(noted by ‘H’), significant changes genes (noted by ‘C’), volcano plot(noted by ‘V’), Wikipathway mapping(noted by ‘W’), and Rectome and KEGG pathway mapping (noted by ‘R’ and ‘K’ respectively).

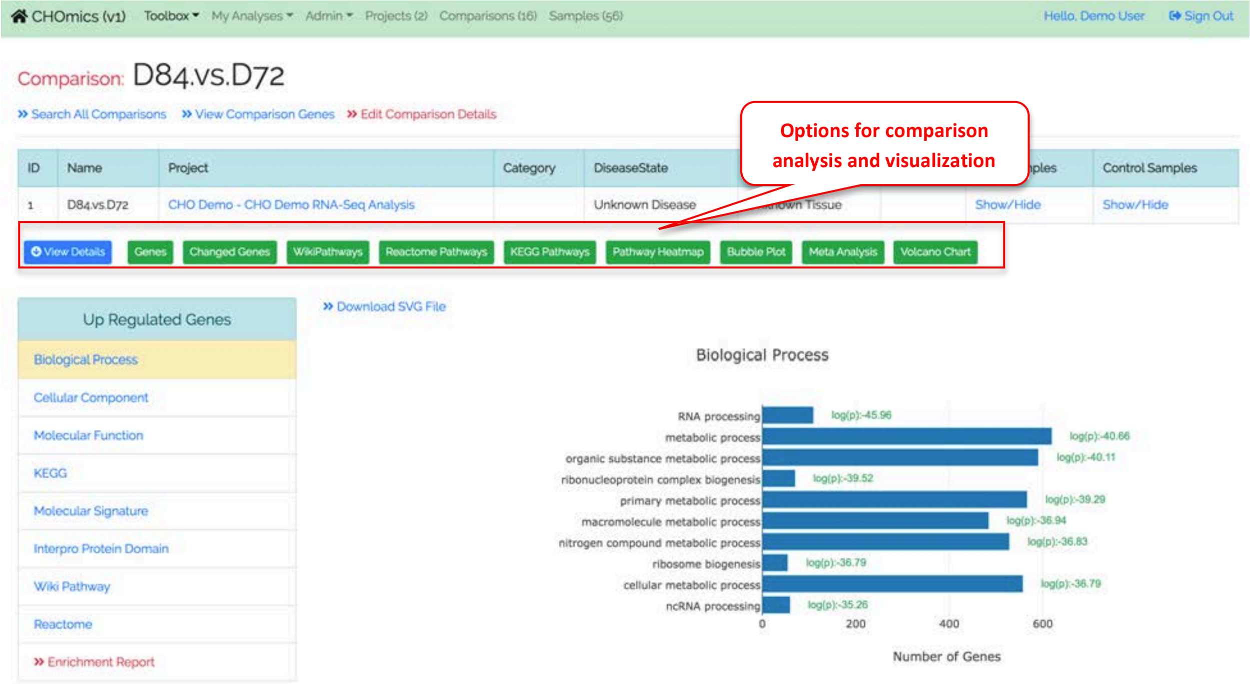

1.6 Genes

The genome-wide gene expression values were detected in each sample using RNA-Seq or microarrays. All the human genes that have expression values are listed in gene table. The gene annotation from difference platforms were all mapped to NCBI gene ID (EntrezID) for consistence across platforms.

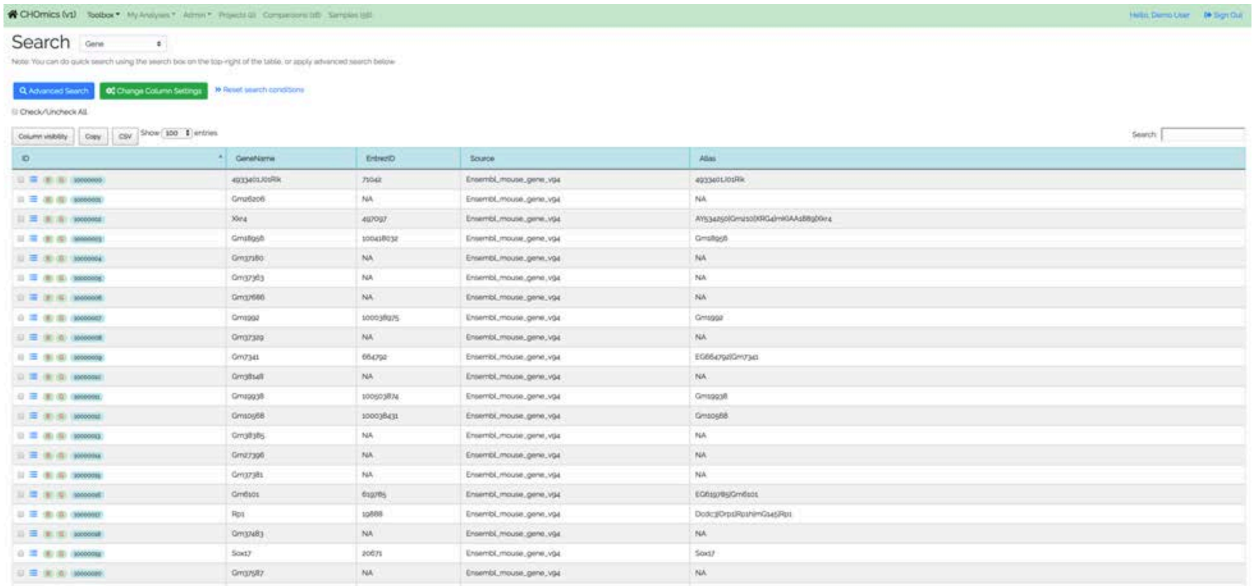

To find a gene, you can use gene symbol, gene description, gene alias, NCBI gene ID, Ensembl gene ID or Uniprot ID.

For some common genes, the symbols used in publications are often not the official symbol, and you can try search alias field. For example, TP53 is often referred to as P53 in publication. You need to search P53 in alias or tumor protein p53 in description to find it if you don't know its official symbol.

The NCBI Gene search https://www.ncbi.nlm.nih.gov/gene is a good source to get official gene symbols and IDs.

You can view full details of a gene by clicking the 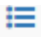 button .

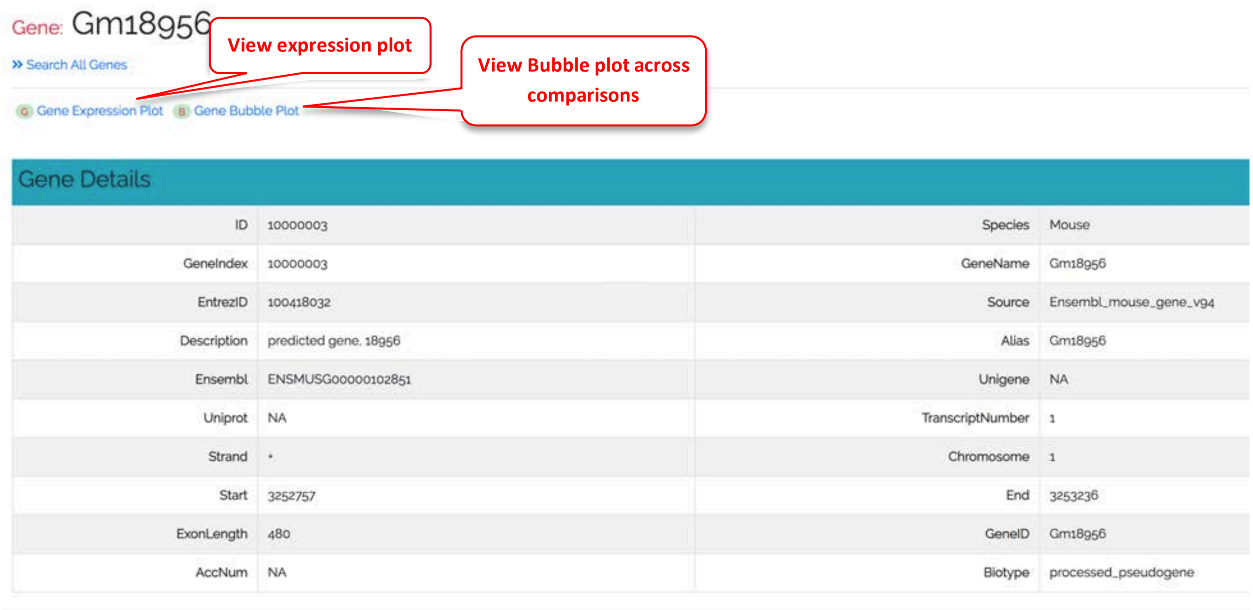

ID	10000003	Species	Mouse
GeneIndex	10000003	GeneName	Gm18956
EntrezID	100418032	Source	Ensembl_mouse_gene_v94
Description	predicted gene, 18956	Alias	Gm18956
Ensembl	ENSMUSG00000102851	Unigene	NA
Uniprot	NA	TranscriptNumber	1
Strand	-	Chromosome	1
Start	3252757	End	3253236
ExonLength	480	GeneID	Gm18956
AccNum	NA	Biotype	processed_pseudogene

From gene details, you can access RNA-Seq data in a box plot, or view all comparisons including this gene in a bubble plot.

2 Data Input

2.1 Upload fastq files to experiment

After the experiment is created, users can upload fastq or fastq.gz files through remote URLs, server files or local files. The files are uploaded to the private folder named ‘Experiments’ automatically.

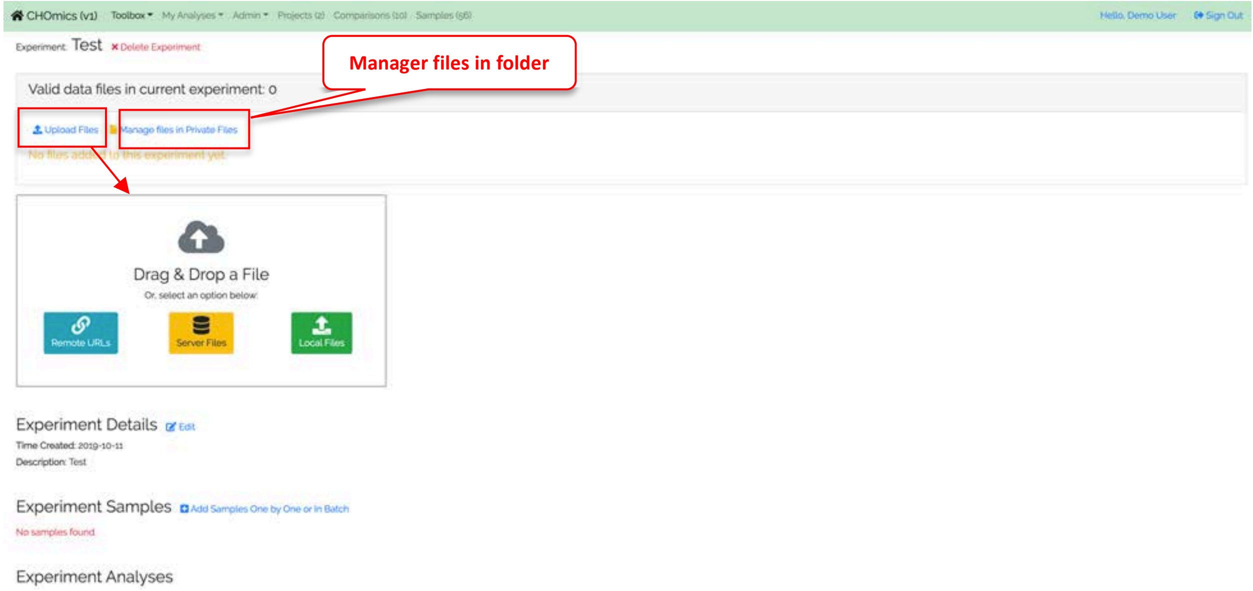

In the folder 'Experiments', there may be multiple subfolders corresponding to different experiments. Users can easily modify the folder or upload new files to the folder.

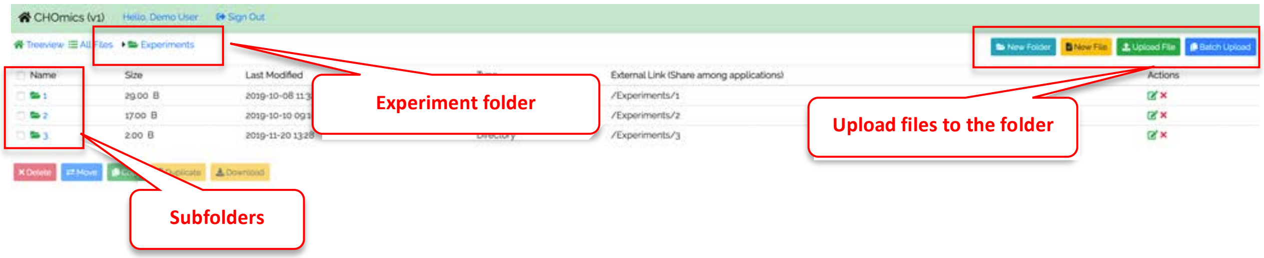

2.2 Upload data file to project

Besides raw RNA sequencing data (fastq files), CHOmics also allow the input of other types of data to start a project, including meta data (i.e,project and samples), expression data, and summary data. Those data should be uploaded in comma separated values (CSV) or tab separated values (TSV) with either

fixed or flexible format.

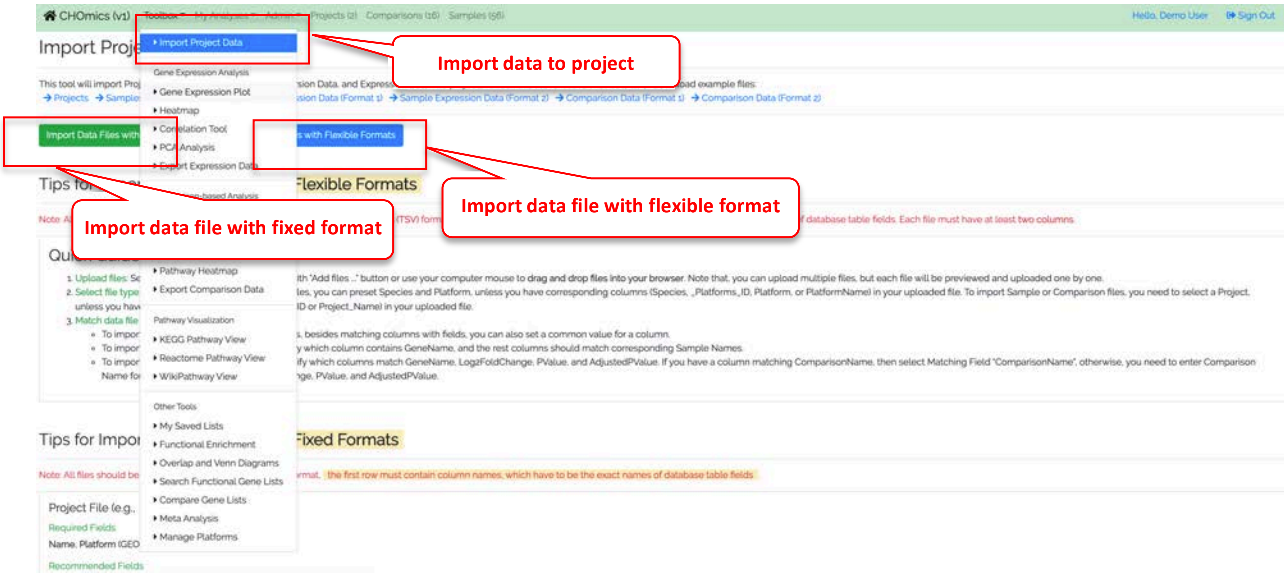

Project file can be uploaded to create a new project. The project file contains some required information such as Name, Platform and other optional fields such as Disease, Description etc.

Sample file can be uploaded to register samples for a project. The sample file contains required information such as Name and Project_Name and optional fields such as Description, Tissue, DiseaseState, SampleSource, Gender, etc.

Expression file can be uploaded with quantified expression measure at gene level. The expression could be transcriptomics, proteomics or other gene-level counts. The file is required to contain GeneName, SampleName, Value.

Comparison file and Comparison data file are used to upload summary results for statistical comparison test applied externally. Comparison file needs to contain the Project_Name, Case_SampleIDs, and Control_SampleIDs while comparison data file contains statistical results such as GeneName, ComparisonName, Log2FoldChange, PValue, Adjusted PValue for each comparison.

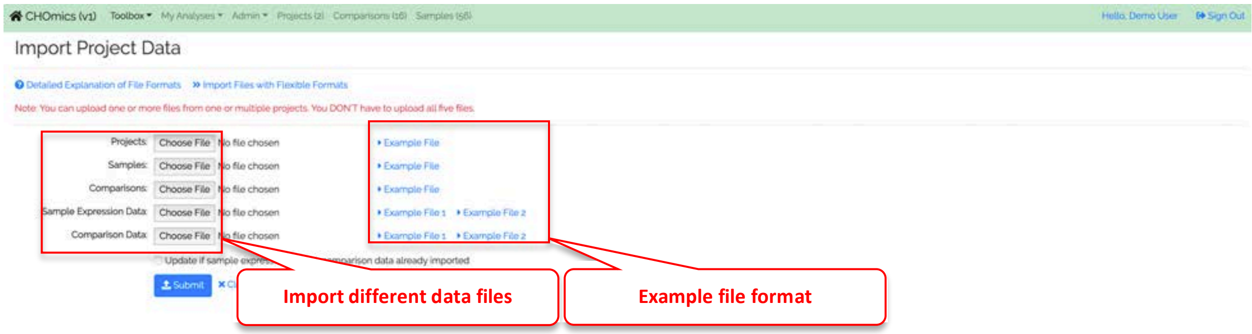

3 Data Analysis

3.1 RNAseq analysis pipeline

After fastq files are uploaded to the experiment by following the Section 2.1, users can start the analysis by applying the built-in pipeline mainly including: Raw Data QC (quality control), Alignment, Gene Counts and QC, and DEG, GSEA and GO analysis. After the analysis is completed, the results can be exported into a project for visualization.

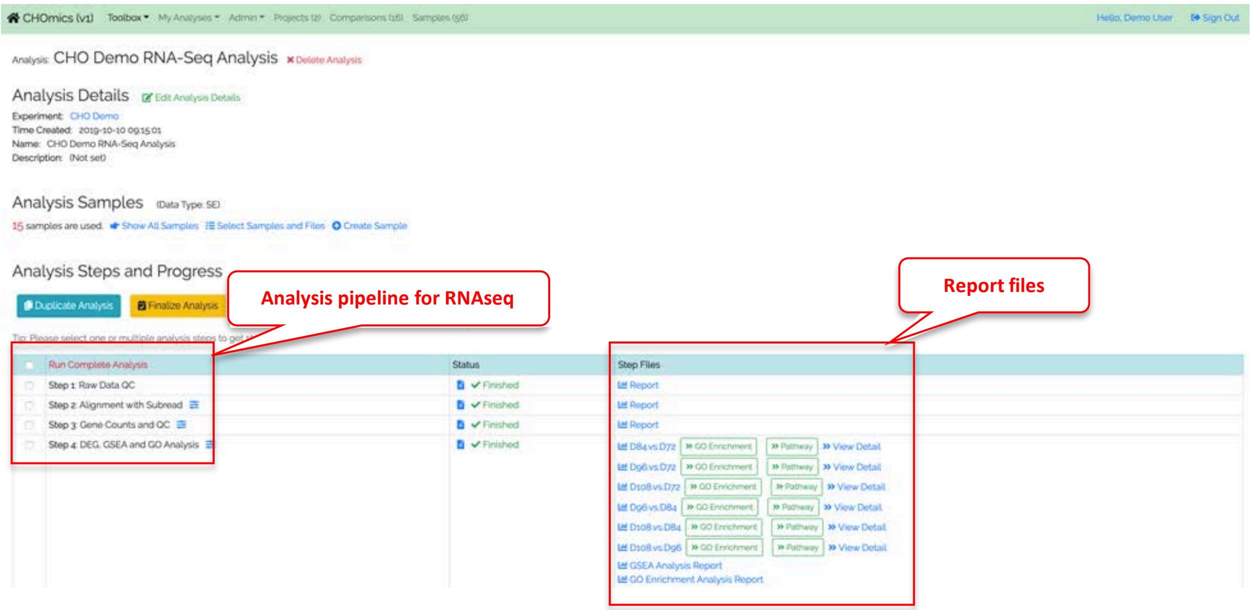

After completion of each step, a report is generated for summarizing the metrics in each step to quantify raw data QC, alignment with Subread method, and gene count distribution and sample/gene count QC, respectively.

In the report for raw data QC, all fastq files are verified in quality by software fastQC. Sequencing read information and quality control metrics are summarized for each individual fastq file.

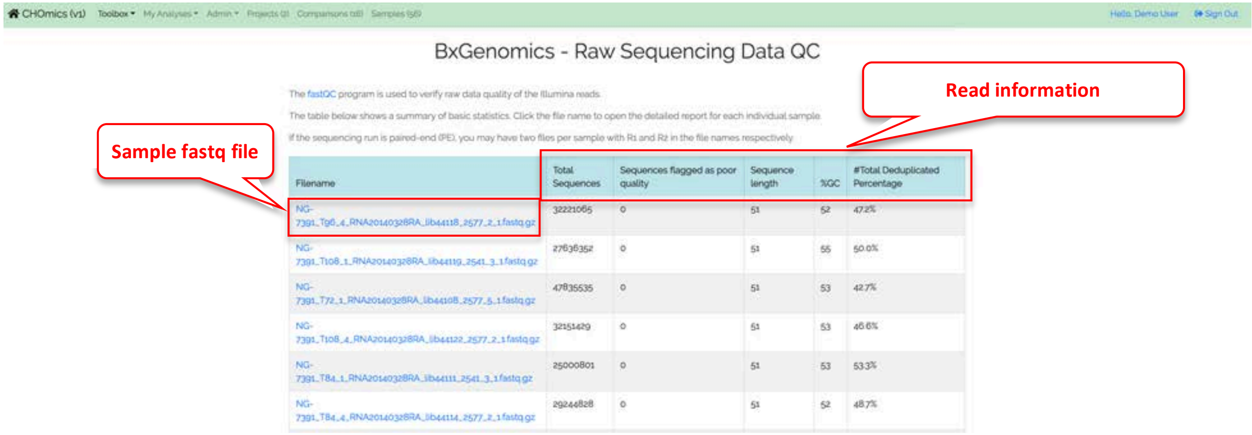

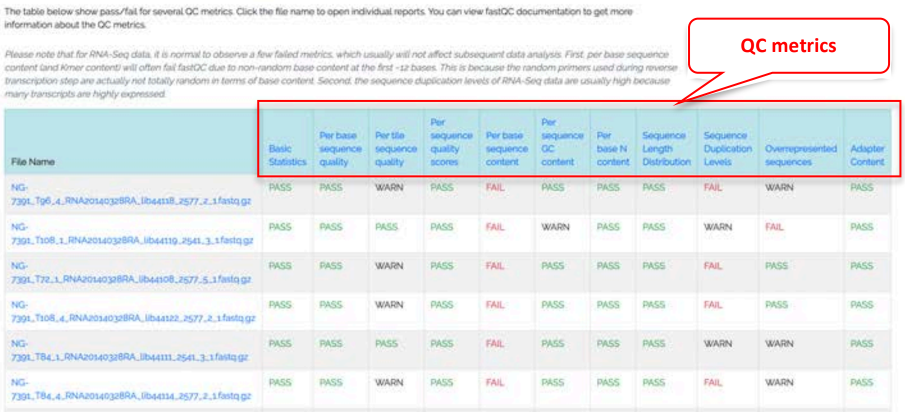

The table below show pass/fail for several QC metrics. Click the file name to open individual reports. You can view fastQC documentation to get more information about the QC metrics.

Please note that for RNA-Seq data, it is normal to observe a few failed metrics, which usually will not affect subsequent data analysis. First, per base sequence content (and Kmer content) will often fail fastQC due to non-random base content at the first ~12 bases. This is because the random primers used during reverse transcription step are actually not totally random in terms of base content. Second, the sequence duplication levels of RNA-Seq data are usually high because many transcripts are highly expressed.

File Name	Basic Statistics	Per base sequence quality	Per tile sequence quality	Per sequence quality scores	Per base sequence content	Per sequence GC content	Per base N content	Sequence Length Distribution	Sequence Duplication Levels	Overrepresented sequences	Adapter Content
NG-7391_T96_4_RNA20140328RA_lib44118_2577_2_1.fastq.gz	PASS	PASS	WARN	PASS	FAIL	PASS	PASS	PASS	FAIL	WARN	PASS
NG-7391_T108_1_RNA20140328RA_lib44119_2541_3_1.fastq.gz	PASS	PASS	PASS	PASS	FAIL	WARN	PASS	PASS	WARN	FAIL	PASS
NG-7391_T72_1_RNA20140328RA_lib44108_2577_5_1.fastq.gz	PASS	PASS	WARN	PASS	FAIL	PASS	PASS	PASS	FAIL	PASS	PASS
NG-7391_T108_4_RNA20140328RA_lib44122_2577_2_1.fastq.gz	PASS	PASS	WARN	PASS	FAIL	PASS	PASS	PASS	FAIL	PASS	PASS
NG-7391_T84_1_RNA20140328RA_lib44111_2541_3_1.fastq.gz	PASS	PASS	PASS	PASS	FAIL	PASS	PASS	PASS	WARN	WARN	PASS
NG-7391_T84_4_RNA20140328RA_lib44114_2577_2_1.fastq.gz	PASS	PASS	WARN	PASS	FAIL	PASS	PASS	PASS	FAIL	WARN	PASS

In the report for alignment, parameter setting and quality metrics (e.g, mapped, junctions,etc) for alignment are listed for each fastq file.

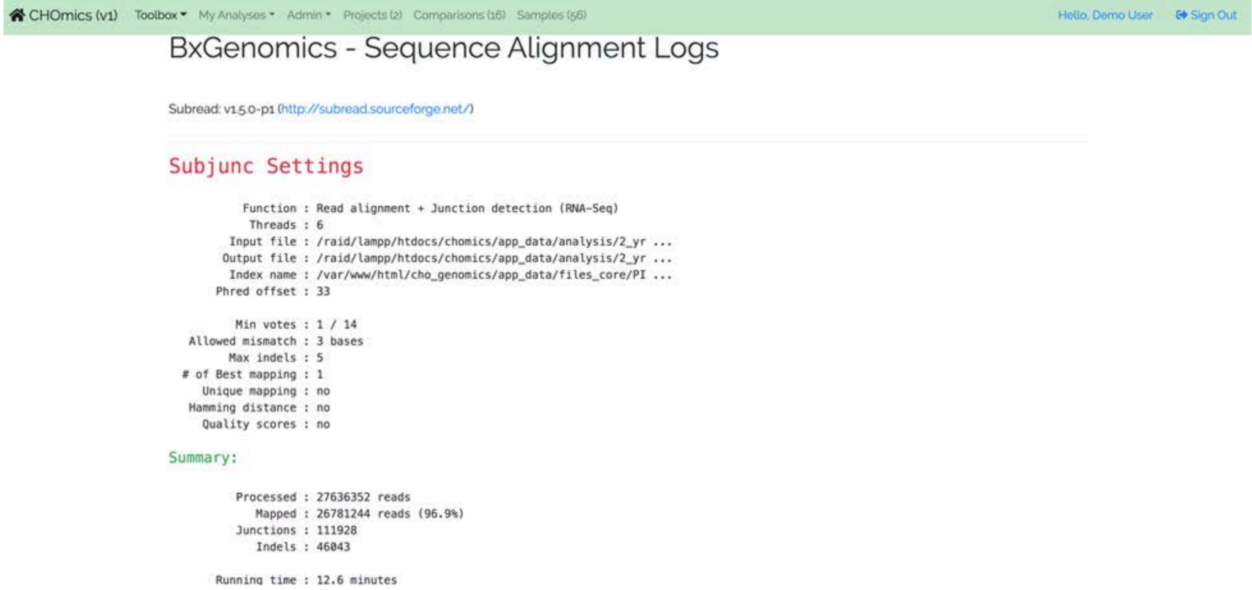

CHOmics (v1) Toolbox My Analyses Admin Projects (2) Comparisons (16) Samples (56) Hello, Demo User Sign Out

BxGenomics - Sequence Alignment Logs

Subread: v1.5.0-p1 (http://subread.sourceforge.net/)

Subjunc Settings

```
        Function : Read alignment + Junction detection (RNA-Seq)
         Threads : 6
      Input file : /raid/lampp/htdocs/chomics/app_data/analysis/2_yr ...
     Output file : /raid/lampp/htdocs/chomics/app_data/analysis/2_yr ...
      Index name : /var/www/html/cho_genomics/app_data/files_core/PI ...
    Phred offset : 33

       Min votes : 1 / 14
Allowed mismatch : 3 bases
      Max indels : 5
# of Best mapping : 1
  Unique mapping : no
Hamming distance : no
  Quality scores : no
```

Summary:

```
       Processed : 27636352 reads
          Mapped : 26781244 reads (96.9%)
       Junctions : 111928
          Indels : 46043

    Running time : 12.6 minutes
```

In the report for Gene Counts and QC step, several metrics have been calculated and plotted for comprehensive evaluation of genes and samples, including: reads mapping to genes, distribution of detected genes, percentage of reads for highly expressed genes, normalization and boxplot of gene expression, sample grouping and clustering, sample correlation and outlier detection.

‘1. Assign reads to genes’ plots the mapping summary of reads to genes, showing the percentage of reads assigned to genes or unassigned due to unmapping, no features or ambiguous mapping.

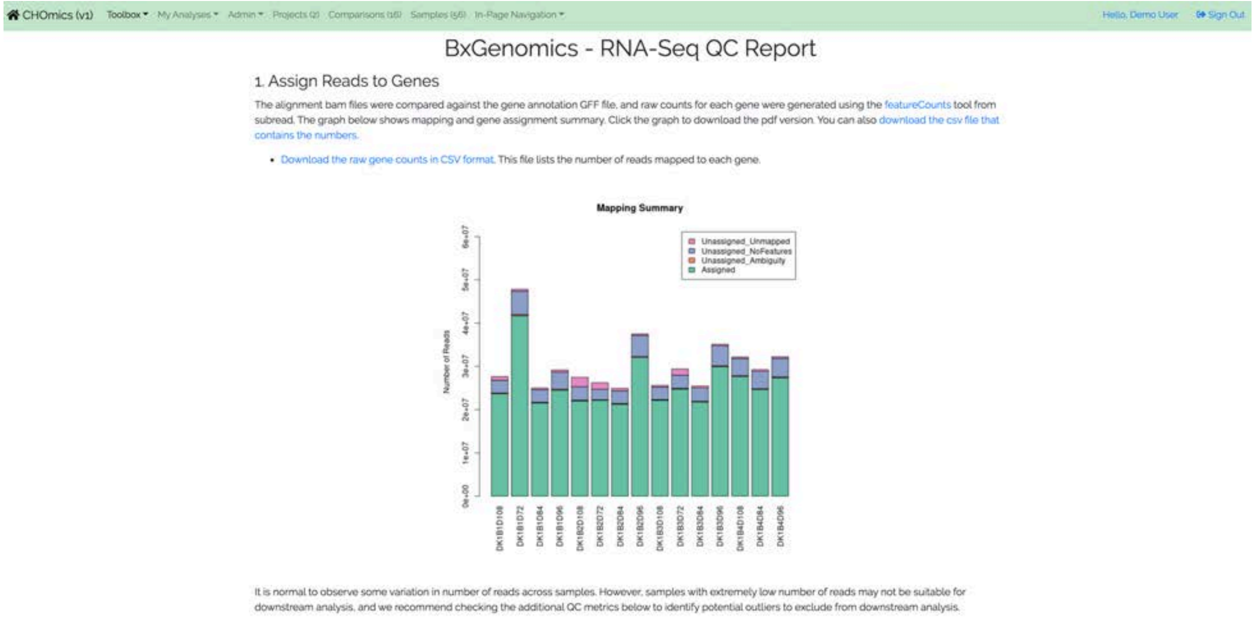

‘2. Number of Genes Detected’ plots the number of expressed genes with read count from intervals of >=1, >=2, >=10, >=50 and >=100.

‘3. Percentage Reads from Most Highly Expressed Genes’ plots the percentage of reads mapped to the top expressed genes (up to 100 genes).

2. Number of Genes Detected

Next, we performed additional QC at gene level. We first looked at number of genes detected. We count the number of genes that have at least 1, 2, 10, 50 or 100 counts. In generally, number of genes with 2 or more counts can be used as a rough estimate of how many genes are expressed. Genes with only 1 read could be noise. In addition, the number of genes with 10 or more reads is a good indicator of how many genes have enough reads for downstream statistical analysis. Click the graph to download a pdf version, you can also download a csv file containing the numbers.

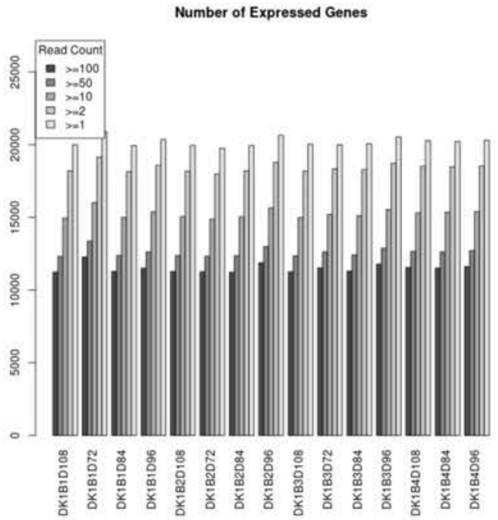

We also try to detect outliers from this step. Any samples that show very small number of genes with 10 or more reads are potential outliers. The cutoff we used is 1/2 of the median across all samples.

3. Percentage Reads from Most Highly Expressed Genes

We also look at the percentage of reads belonging to the top genes. Basically we rank the genes by read counts, and compute the percentage of reads belonging to the top genes (up to top 100).

If majority of the reads come from top genes, then the sample probably has bottlenecking issues where a few genes were amplified many times by PCR during library preparation.

Most samples should have ~ 20% reads mapped to the top 100 genes.

If the top 100 genes account for more than 35% of all reads, we consider this sample as a potential outlier.

Click the graph to download a pdf version. You can also download a csv file download the csv file that contains the numbers.

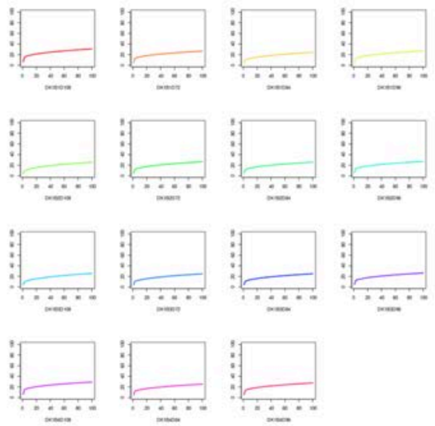

'4. Normalization and Boxplot of Gene Expression' evaluates gene expression after normalization by TMM method and then draws boxplot of normalized expression (logCPM: log of counts per million reads) after log2 transformation.

4. Normalization and Boxplot of Gene Expression

The raw counts data were further processed by the following steps:

a) Remove genes that were not expressed. If a gene has counts per million (CPM) value >=1 in at least two of the samples, we consider it expressed in the experiment and include it for downstream QC analysis. **From 32871 total genes, 14212 genes are selected as expressed and used in downstream QC analysis.**

b) The TMM normalization method was used to scale samples to remove differences in the composition of the RNA population between samples. It is performed with the edgeR package. The normalization factors for all samples are listed below. You can download the csv file.

At this step, we also try to identity outliers that have extreme normalization factors (>1.5 or <0.66). Note sometimes samples with large biological differences can have extreme normalization factors.

Name	group	lib.size	norm.factors
DK1B1D108	1	23619476	0.927
DK1B1D72	1	41669151	1.010
DK1B1D84	1	21522805	1.028
DK1B1D96	1	24476888	0.995

c) The normalized gene counts were transformed to log2 scale using voom method from the R Limma package. We created boxplot for each sample to summarize gene expression.

Since this is normalized data, most samples should look similar. Samples with high or low distribution may be outliers (or have large biological differences).

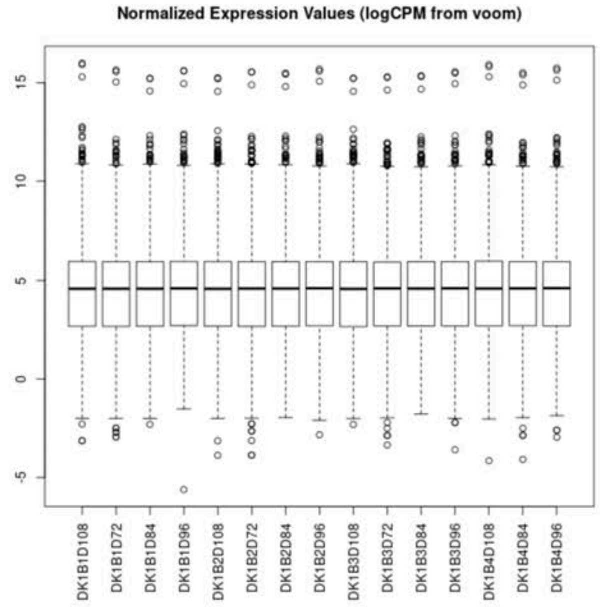

'5 Grouping and Clustering of Samples' plots the relationship among samples by multidimensional scale. Samples are clustered by hierarchical clustering method based on the expression of top genes with large variation(SD/mean>0.3).

5. Grouping and Clustering of Samples

a) We first create multidimensional plot to view sample relationships. This is done using R Limma package.

Here biological replicates should cluster together, and difference conditions ideally should separate from each other.

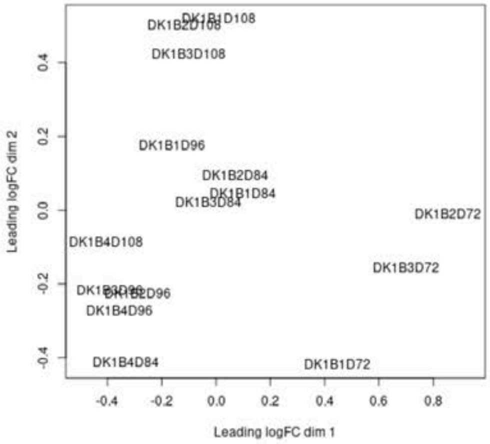

b) Very often hierarchical clustering can give better indication of the sample and gene relationships. We used made4 package from R to cluster samples and draw a heatmap.

We selected genes that have variable expression across samples to make the heatmap. These variable genes were chosen based on standard deviation (SD) of expression values larger than 30% of the mean expression values (Mean). If there are more than >5000 variable genes, we first remove genes with mean logCPM<1, then rank genes by SD/Mean to get the top 5000 genes.

The heatmap is created from 1124 variable genes.

In the heatmap above, we selected genes that changed across samples (normally by SD/mean > 0.3), and plotted the relatively gene expression levels (blue is low, red is high). Gene names are not shown due to large number of genes used to create the heatmap. Both genes and samples are clustered in the heatmap. Normally biological replicates should cluster together, and ideally there should be up- or down- regulated genes between different conditions.

Heatmap can be used to detect overall patterns, as well as outlier samples.

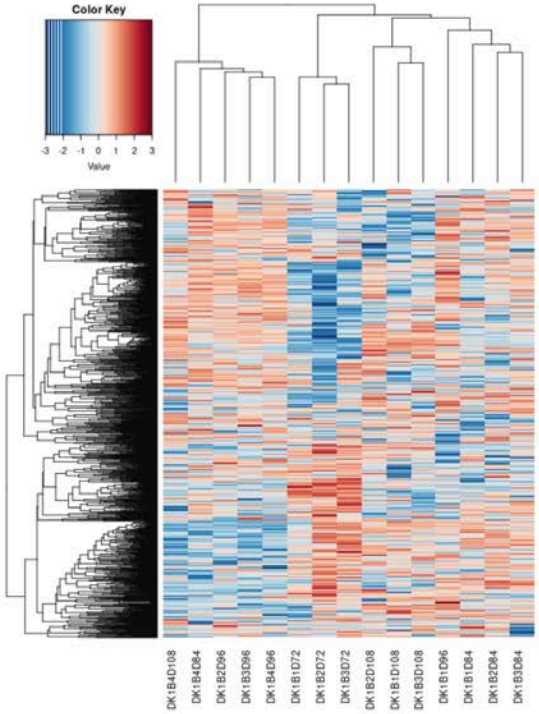

'6 Sample correlation' creates scatter plots for the correlation between sample pairs. The idea is that biological replicates from the same group should look similar in the scatter plot, and should have high correlation values compared to the samples from other groups.

6. Sample correlation

We also created scatter plots for the correlation between sample pairs. If there are many samples, you may need to download the graph and view it at full size. Again, the idea here is that biological replicates should look similar in the scatter plot, and should have high correlation values.

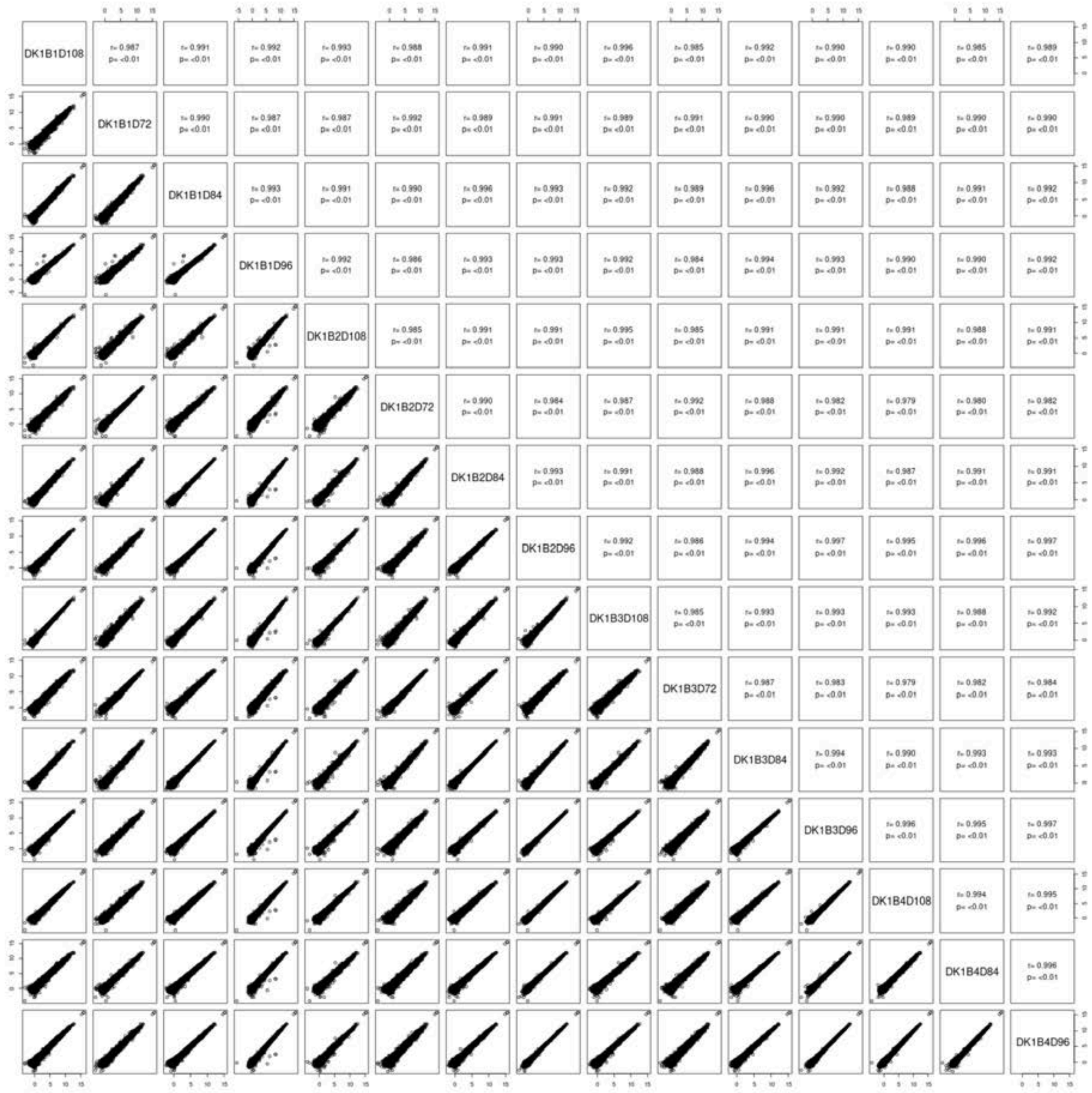

3.2 DE, GSEA and GO analysis

After completing the first three steps for sample quality control and gene count readout, users can start statistical analysis as the last step of pipeline, including differential expression analysis (DEG), gene set enrichment analysis (GSEA) and gene ontology (GO) analysis.

DEG analysis is applied to compare gene expression between two groups, namely comparison. Users can design one or multiple comparisons for DEG analysis. In each comparison, differential expressed genes are identified by LIMMA model, followed by GSEA pathway analysis and GO enrichment analysis which explore the enrichment of DEGs in diverse pathways.

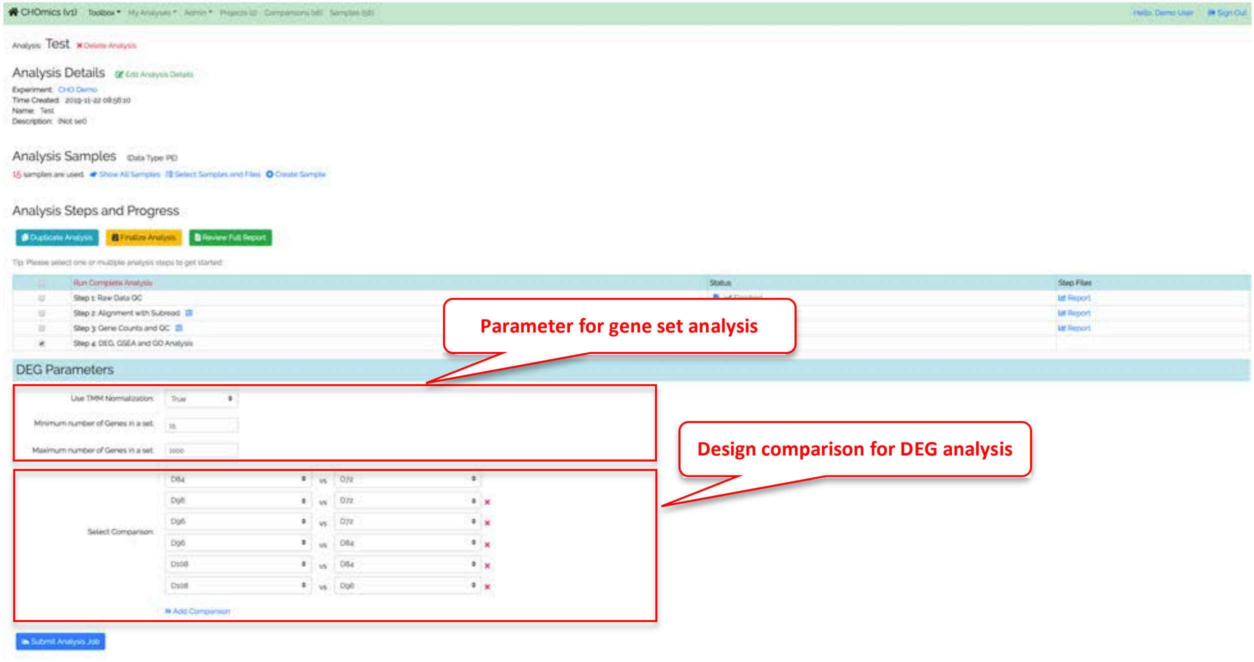

The reports for DEG and pathway analysis are attached for each comparison after completion of analysis.

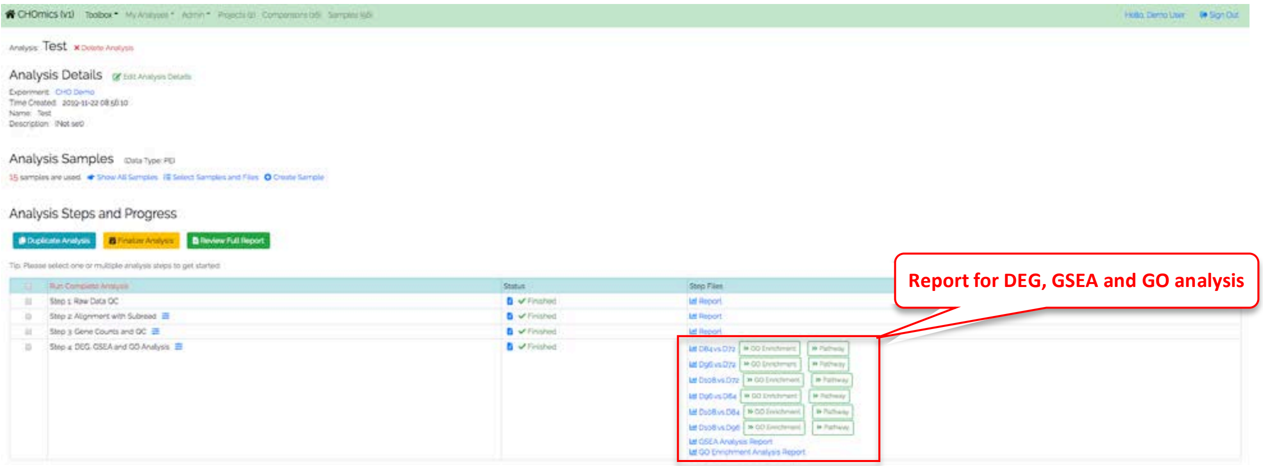

In the report for DEG analysis, the table summarizing DEGs with up- and down-regulation is listed along with a heatmap clustering the DEGs expression (up to top 1000 DEGs).

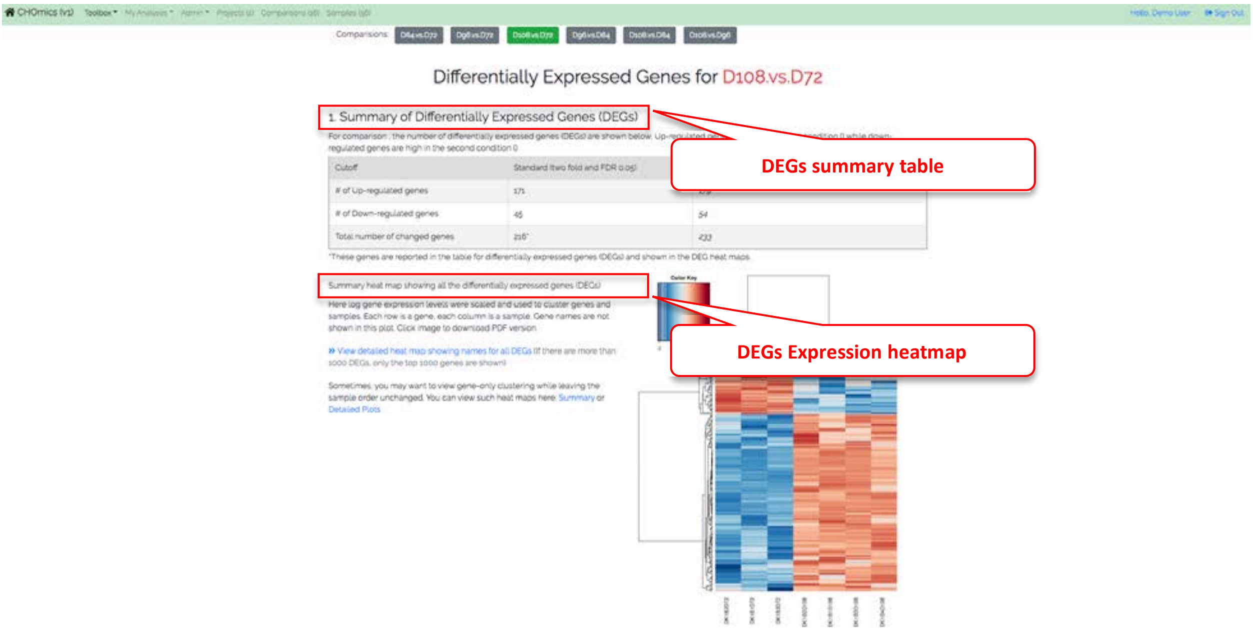

In the report for GO enrichment analysis, barplots show the significance of enrichment of up- or down-regulated DEGs in different pathway databases, e.g, GO, KEGG, Wiki pathways, etc.

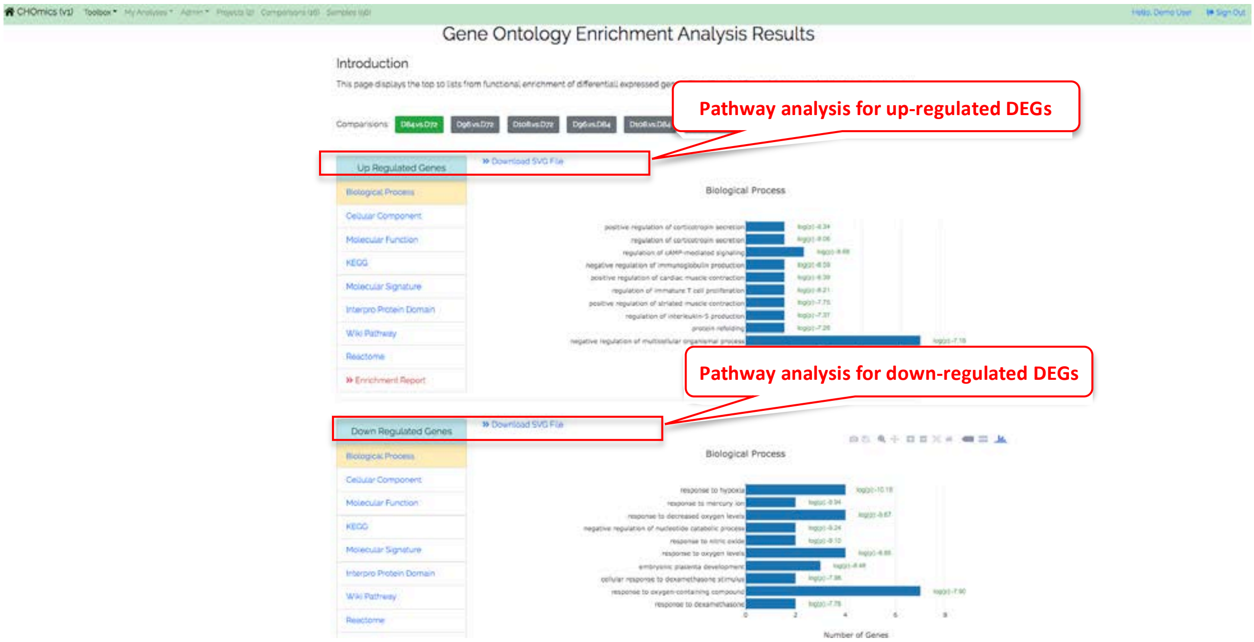

Similarly, in the report for GSEA analysis, enrichment results for up- and down- regulated DEGs in pathways from MigDB database are plotted with significance level (FDR), respectively.

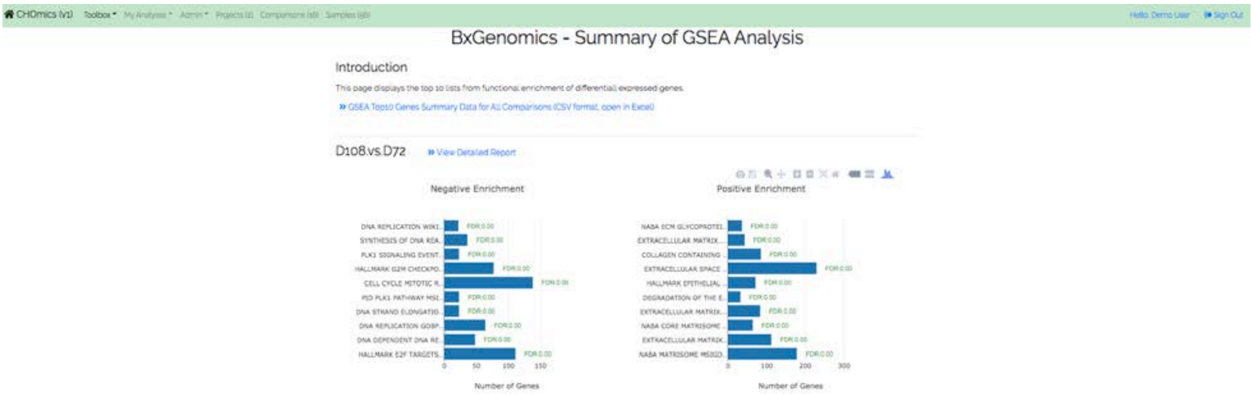

3.3 Saved Genes and Comparisons

Customers can save selected genes or comparison for future use (e.g. multiple gene and multiple comparisons bubble plot). From gene search, check the genes you want to save, and click the yellow button "save selected genes

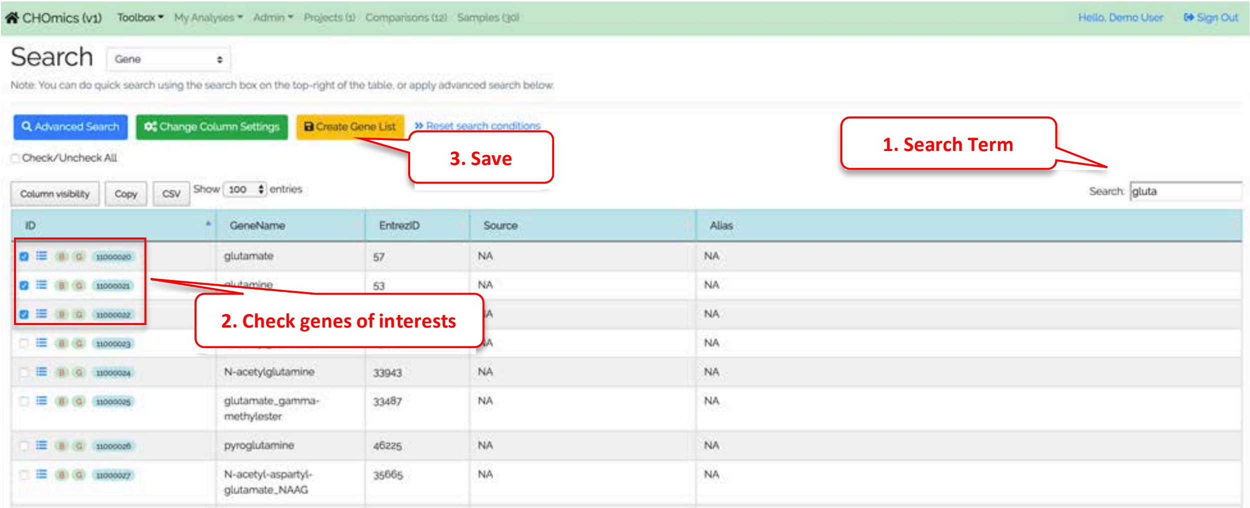

You can do the same with comparisons.

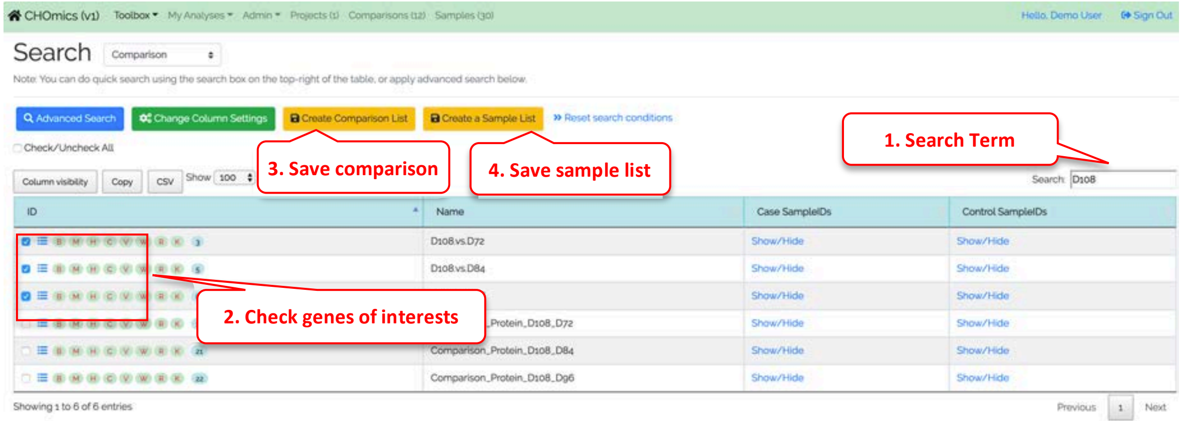

To view selected genes or comparison, click “My Results” link on the left menu and then “Gene Lists”.

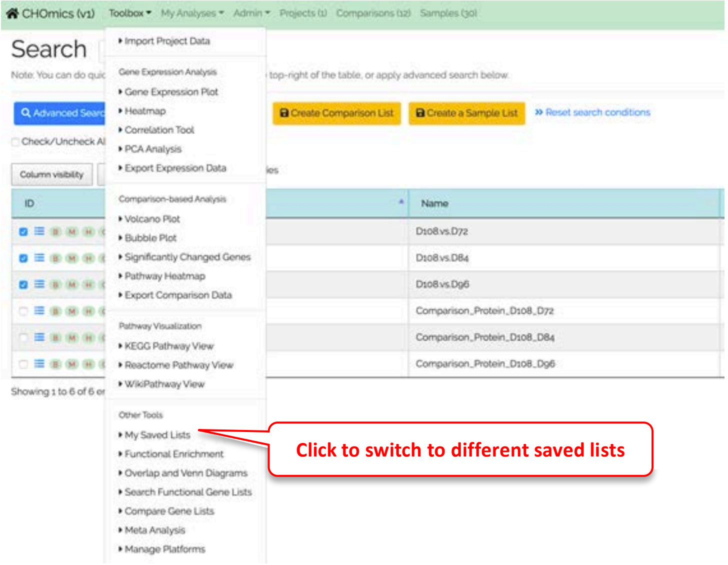

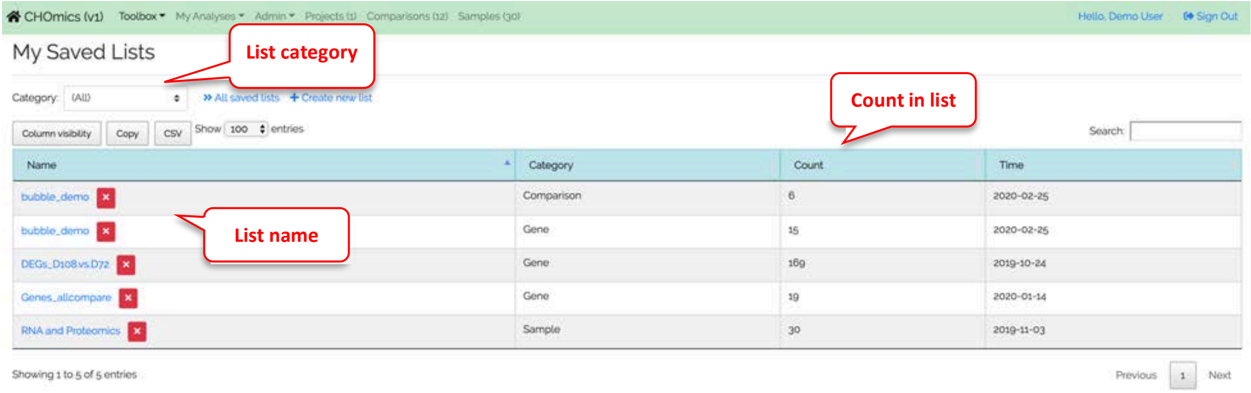

One additional way to select and save genes or comparisons is from the ‘significant changed genes’ in toolbox. Using the dynamic filters to choose the comparisons or genes you are interested in, and you can use the table at the bottom to save comparisons or genes.

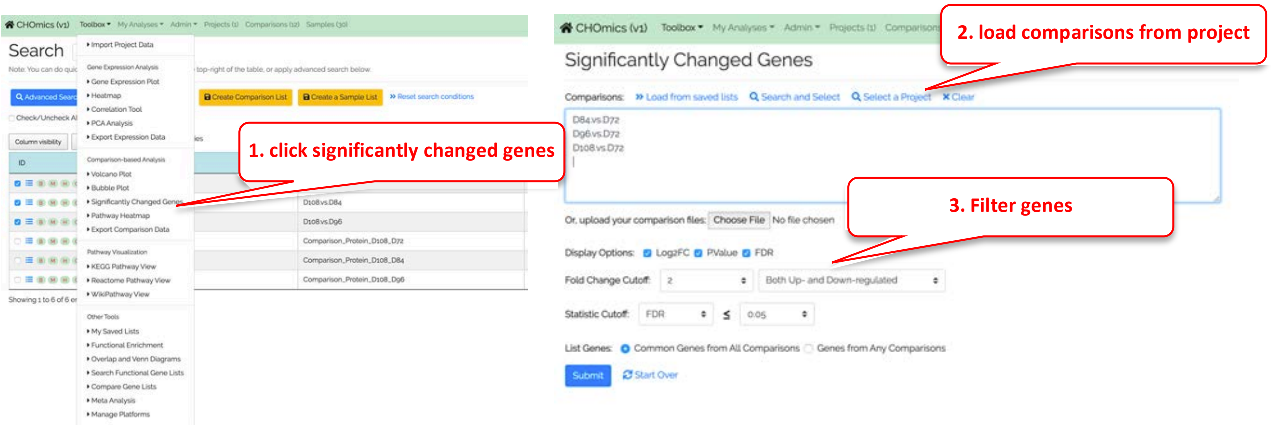

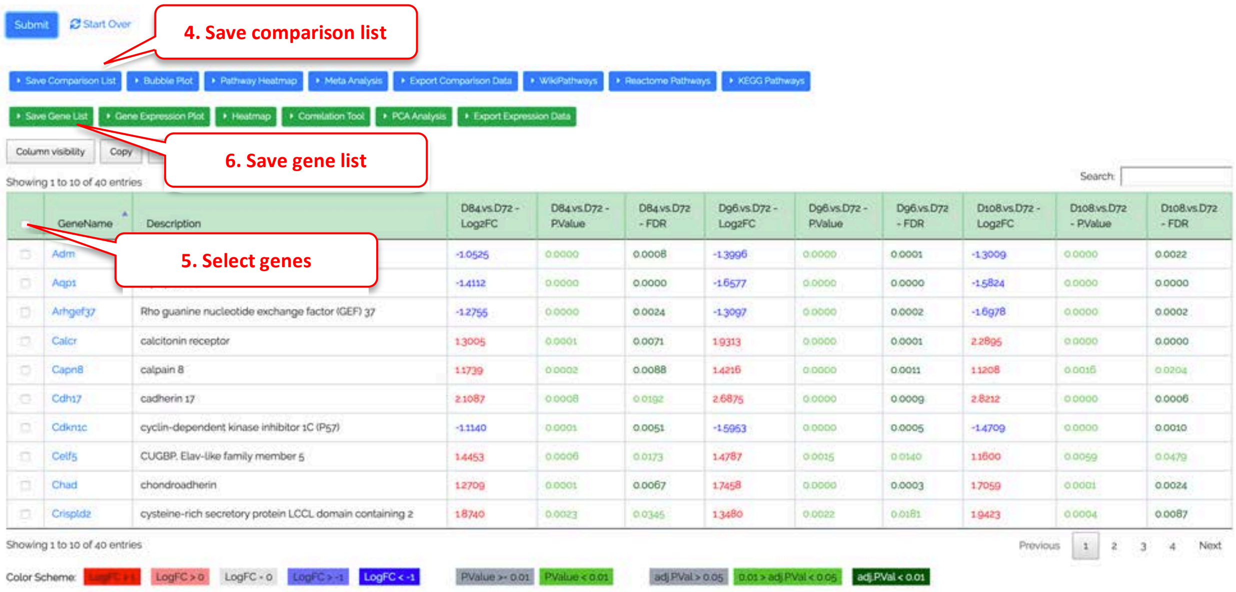

3.4 Advanced Analysis

Besides the above analyses, the CHOmics also provides several advanced tools.

3.3.1 Correlation Tools

Once the user has identified a gene of interest, the user can use correlation tools to find other genes that share similar (or opposite) profiles in terms of gene expression or fold change. First, enter the gene of interest, and samples to be used for correlation. In the example below, we entered a saved gene list, and 15 samples.

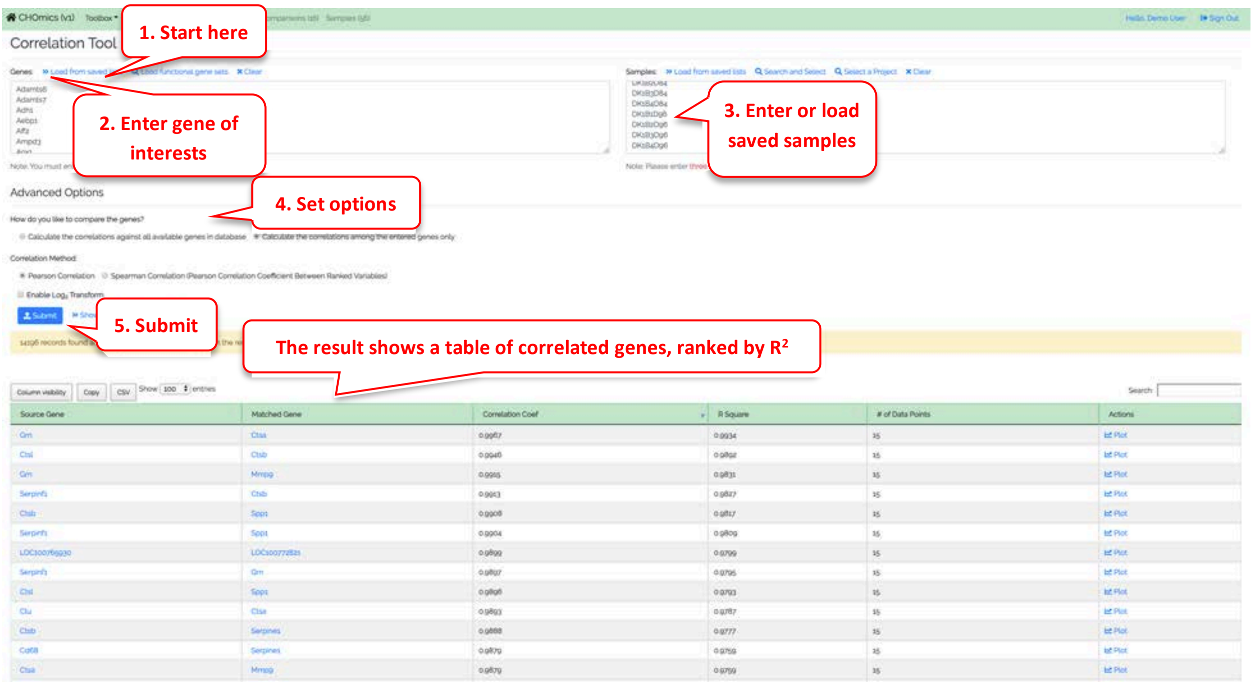

Click the plot icon will show scatter plot of the target and the correlated gene (e.g, gene Grn vs. Ctsa).

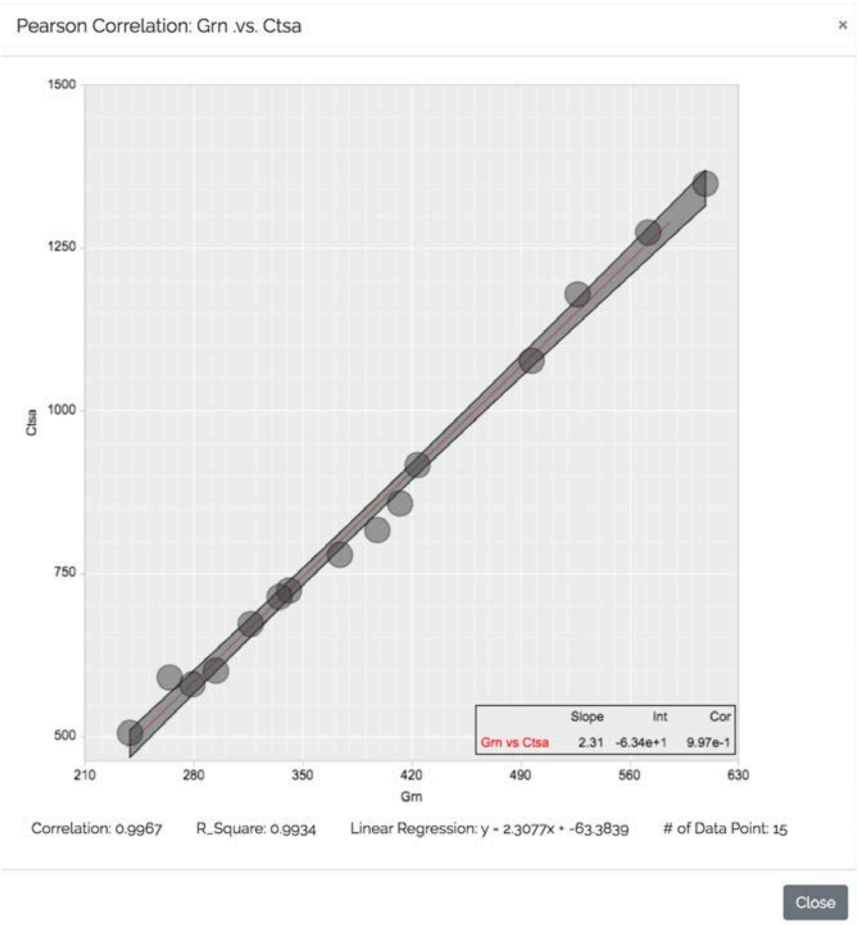

3.3.2 PCA Analysis

You can select a set of samples and genes and use PCA plot to visualize the sample relationships on the target gene set.

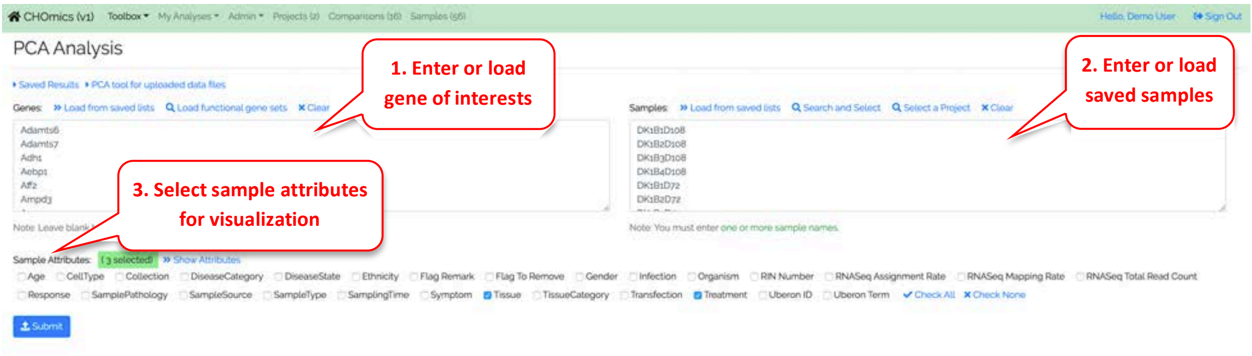

The system will use FactorMineR package to run PCA analysis and display the results. Several PCA metrics are plotted for interactive visualization:

Eigenvalues plot the percentage of variance explained by top PCs.

Variables Plot shows the weights of top contributing genes in each PCs.

Variable Data summarizes the weights of each gene in each individual PC.

Individuals Plot shows the relationship of samples on the spanned space by different PCs.

Individual Data summarizes the score vector of each sample in each individual PC.

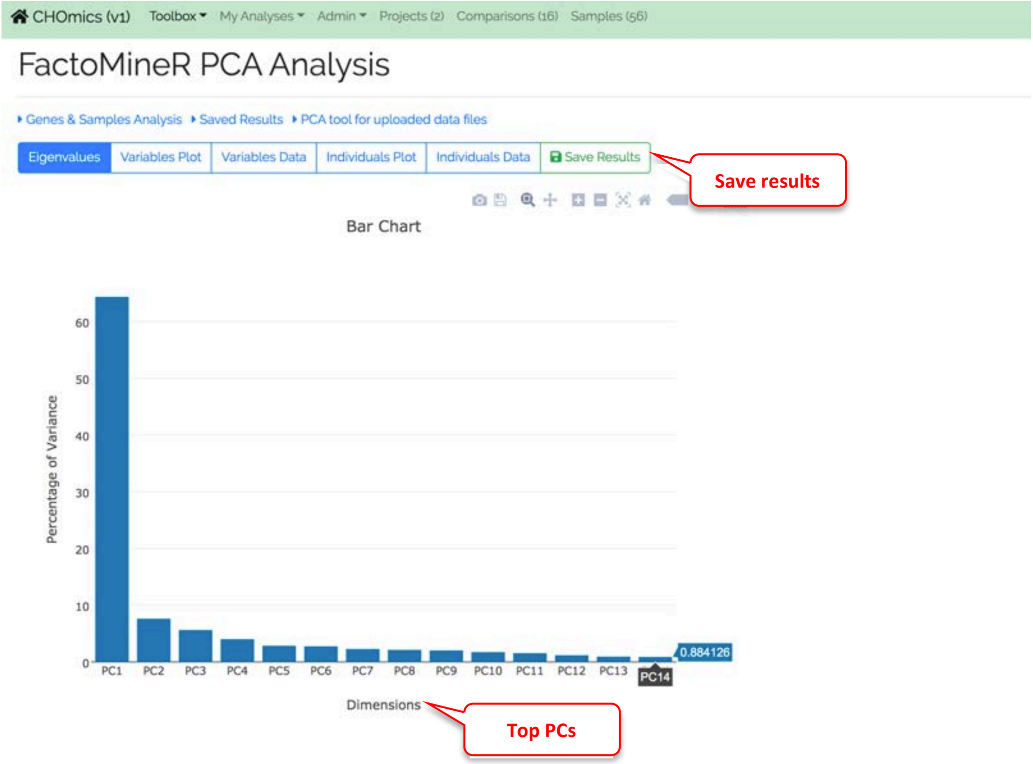

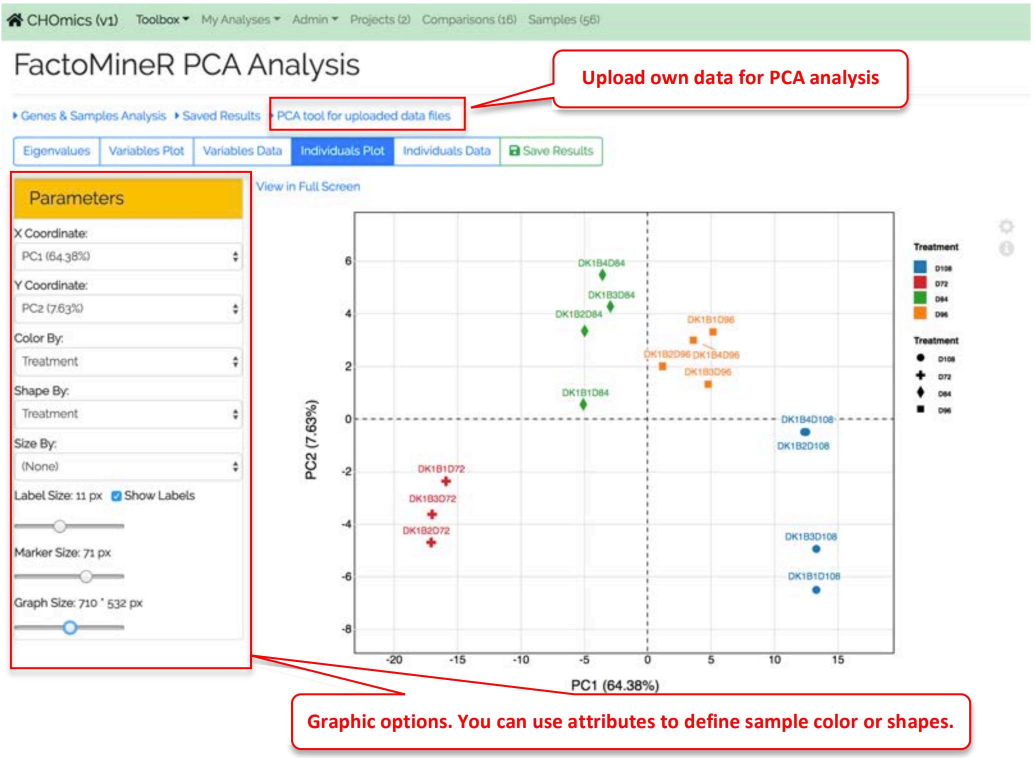

The PCA results can be saved. Users can load it in the future.

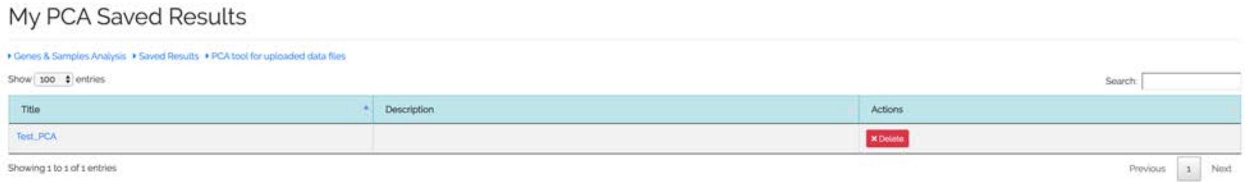

Users can upload their own data matrix or pre-calculated data for PCA analysis and visualization.

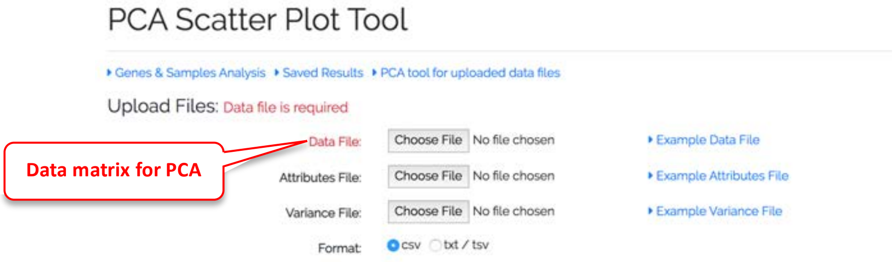

3.3.3 Meta-Analysis

Meta-Analysis can be used to identify genes that are changed consistently across multiple projects. It is listed as one functional module in toolbox panel. In the example below, we are looking for the most significant DEGs in three comparisons.

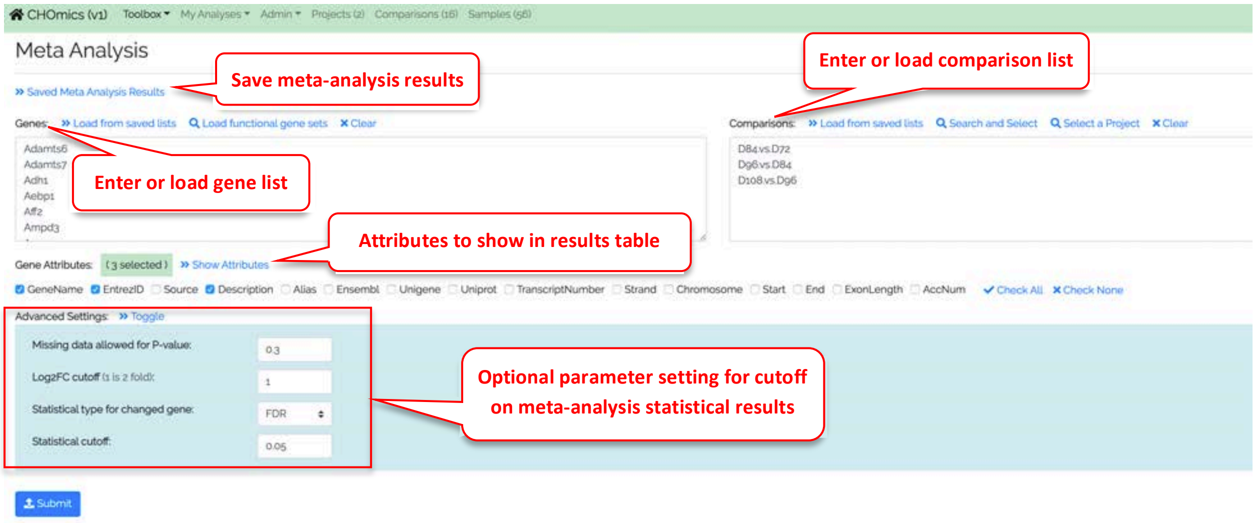

The meta-analysis pipeline will compute three types of results:

1) Maximum p-value (maxP). This method targets on DEGs have small *p*-values in "all" comparisons. We recommend using maxP if you are looking for DEGs that are common among several studies.
2) Fisher's p-value. The Fisher's method sums up the log-transformed p-values obtained from individual studies. This p-value combination method is useful if you want to identify DEGs in any of the comparisons.
3) We also applied simple counting method to report the frequency a gene is classified as up or down-regulated DEG from all the comparisons. The default DEG cutoff is two-fold change and FDR<0.05. but user can change the cutoff.

In most cases, combing maxP (smaller values are more significant) and the counting method (e.g. up-regulated in 50% of studies) will give the most biological relevant results for consistently regulated genes across comparisons.

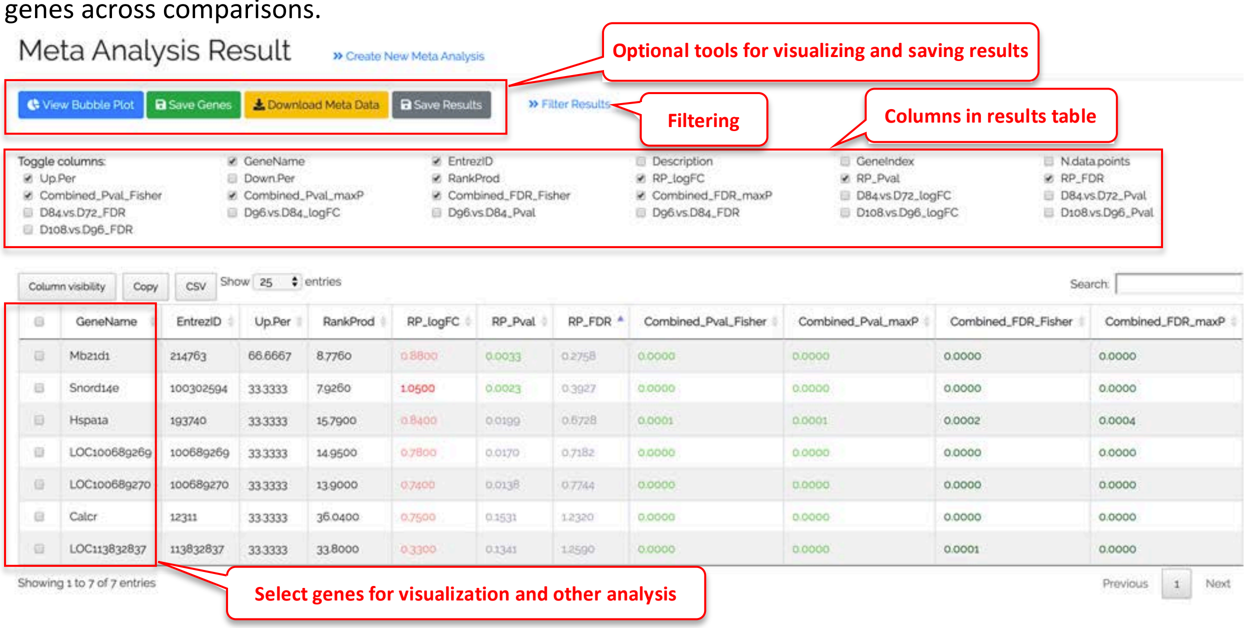

GeneName	EntrezID	Up.Per	RankProd	RP_logFC	RP_Pval	RP_FDR	Combined_Pval_Fisher	Combined_Pval_maxP	Combined_FDR_Fisher	Combined_FDR_maxP
Mb21d1	214763	66.6667	8.7760	0.8800	0.0033	0.2758	0.0000	0.0000	0.0000	0.0000
Snord14e	100302594	33.3333	7.9260	1.0500	0.0023	0.3927	0.0000	0.0000	0.0000	0.0000
Hspa1a	193740	33.3333	15.7900	0.8400	0.0199	0.6728	0.0001	0.0001	0.0002	0.0004
LOC100689269	100689269	33.3333	14.9500	0.7800	0.0170	0.7182	0.0000	0.0000	0.0000	0.0000
LOC100689270	100689270	33.3333	13.9000	0.7400	0.0138	0.7744	0.0000	0.0000	0.0000	0.0000
Calcr	12311	33.3333	36.0400	0.7500	0.1531	1.2320	0.0000	0.0000	0.0000	0.0000
LOC113832837	113832837	33.3333	33.8000	0.3300	0.1341	1.2590	0.0000	0.0000	0.0001	0.0000

In the above example, we used a relatively loose filtering criterion (N.data.points>1, and up-regulation in percentage>30% of studies, and Combined_Pval_MaxP <=0.0001) because only small number of genes pass the stringent default criterion.

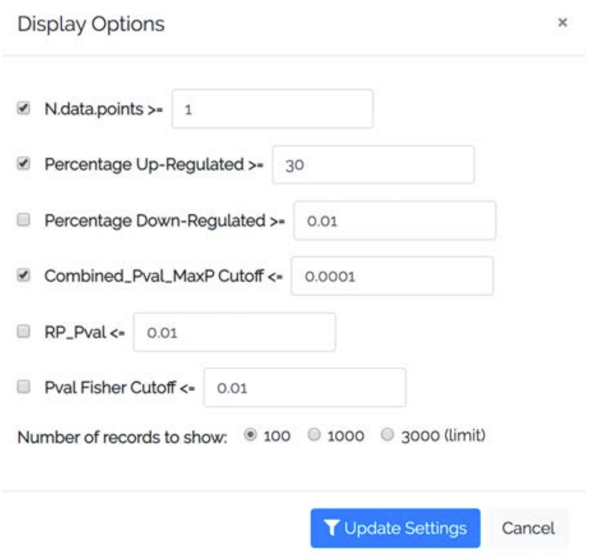

The data table shows the genes that pass the filters. We can sort the table by maxP value. A different filter can be applied to get down-regulated genes.

The results can be saved for future access. There are also links to several other tools. The download meta data link will save a CSV file that contain results from all genes.

Next, we will choose all the genes that pass filter by checking the box for all listed genes, and use bubble plot to visualize the results.

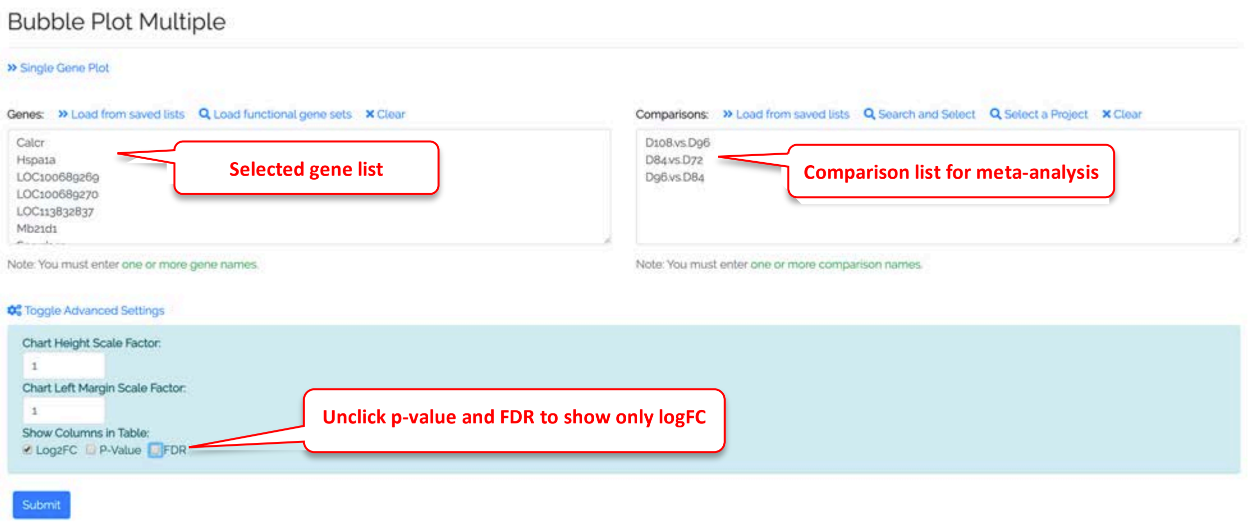

The resulting bubble plot will show all three comparisons for each gene.

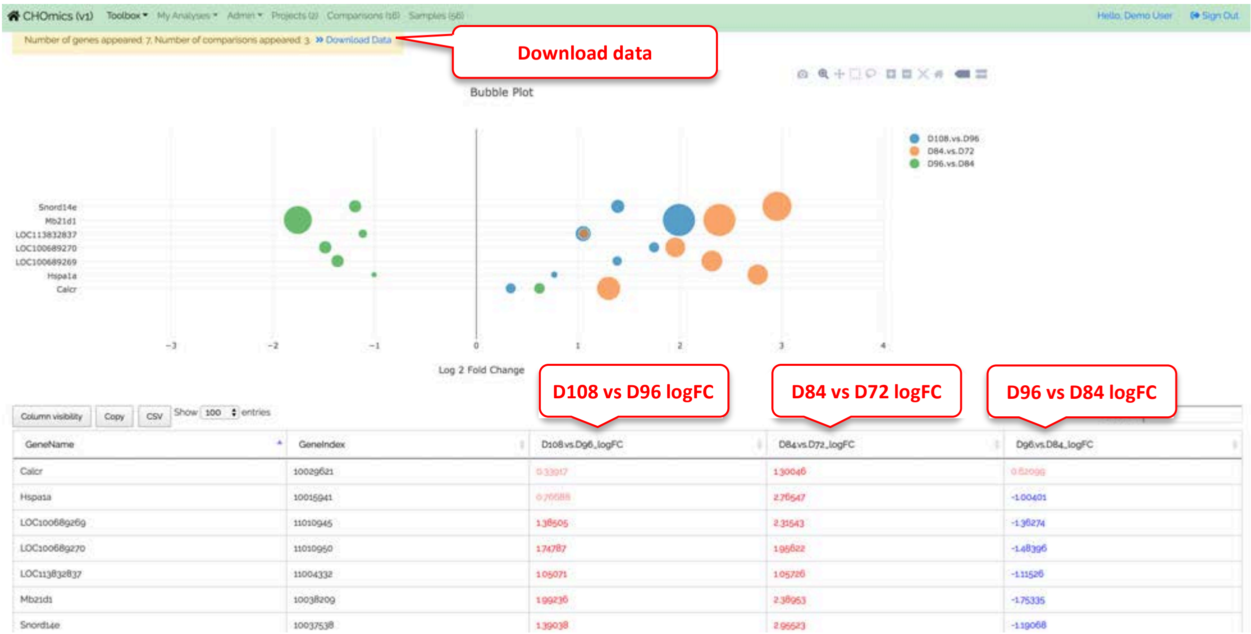

The data table below the bubble plot can also be used for filtering. Remember in the advanced settings, we choose to display logFC only, this makes it easier to look for genes that are reverted in different time points. The logFC values are colored coded (red, increase, blue, decrease), therefore we can see that most of genes show upregulation in D84, and then downregulation in D96 and then upregulation in D108.

You can also redo the plot, check all columns to include p-value and FDR in the table, and export the results to excel file.

The workflow above uses up-regulated genes as example. You can get down-regulated genes from the filter step in meta-analysis result page.

4 Visualization

4.1 Visualize Gene Expression

CHOmics provides tool to easily visualize gene expression level across multiple genes, samples and omics. For each gene, you can view its expression levels across multiple samples.

4.1.1 View Gene Expression from multiple samples

Choose the Gene Expression tool from Toolbox -> Gene Expression Plot from top menu, and enter the official symbol of genes or load gene list from saved lists. Alternatively, in the gene details page, click View Gene Expression link.

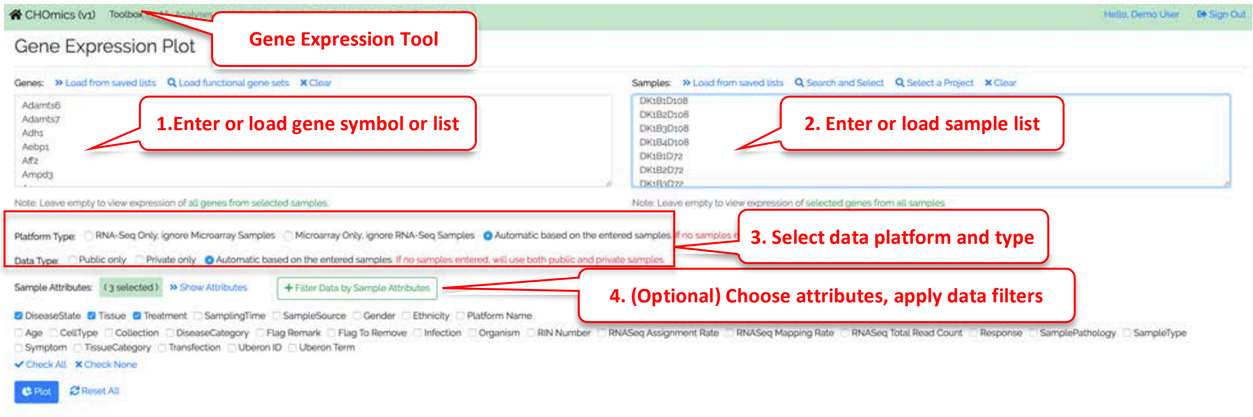

As an optional step, you can choose what sample attributes to pass to the plot, and use data filter to choose only a subset of data points.

The Data Filter can be very useful if there are too many data points, and you want to focus on a few diseases or tissue types.

The screenshots below show default boxplot showing all samples by different time points (i.e, treatment).

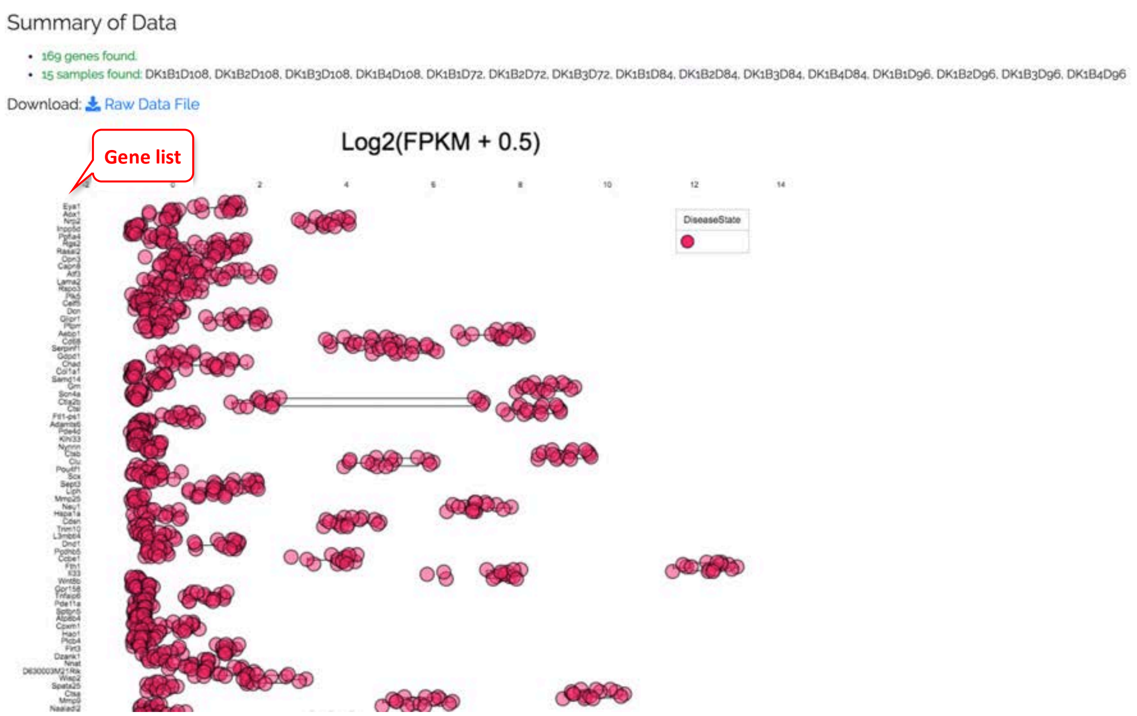

Customize Gene Expression Plot

The boxplot is created using CanvasXpress (https://canvasxpress.org)plug-in, and sample grouping and coloring can be customized by the user. In the example below, we show how data points are colored.

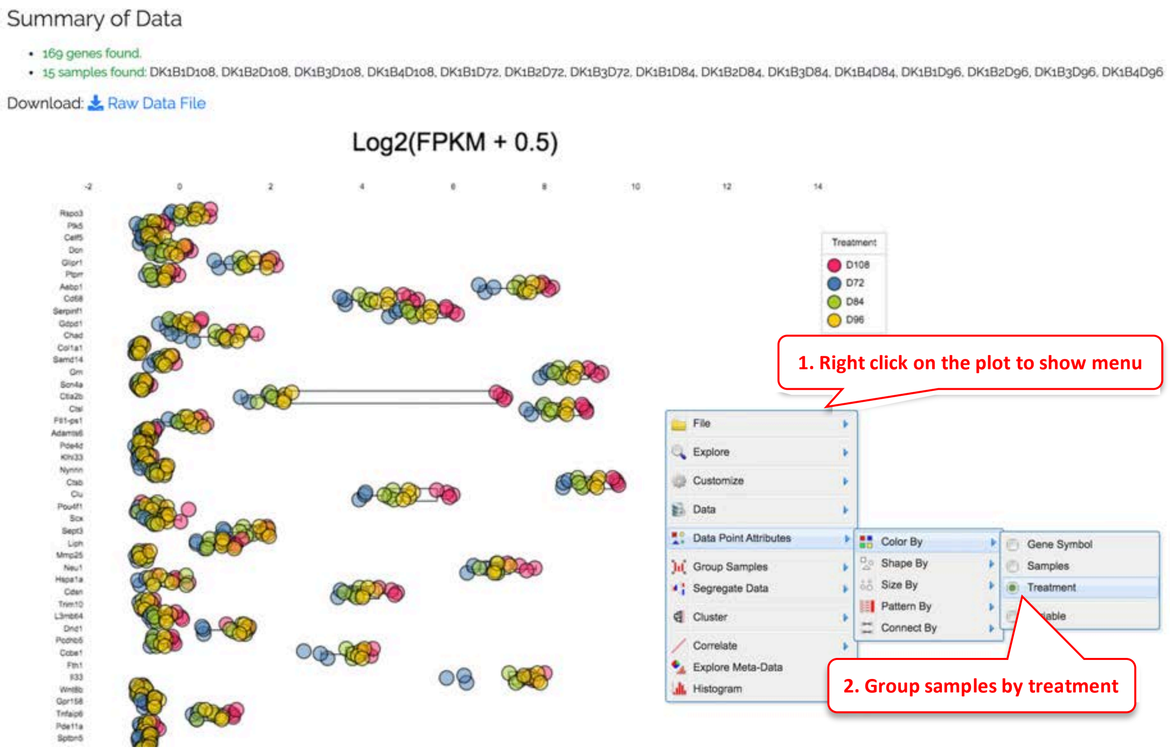

4.1.2 View Gene Expression in Heatmap

Heatmap can be useful to visualize gene profiles from multiple samples. It can also provide information about how genes and samples cluster.

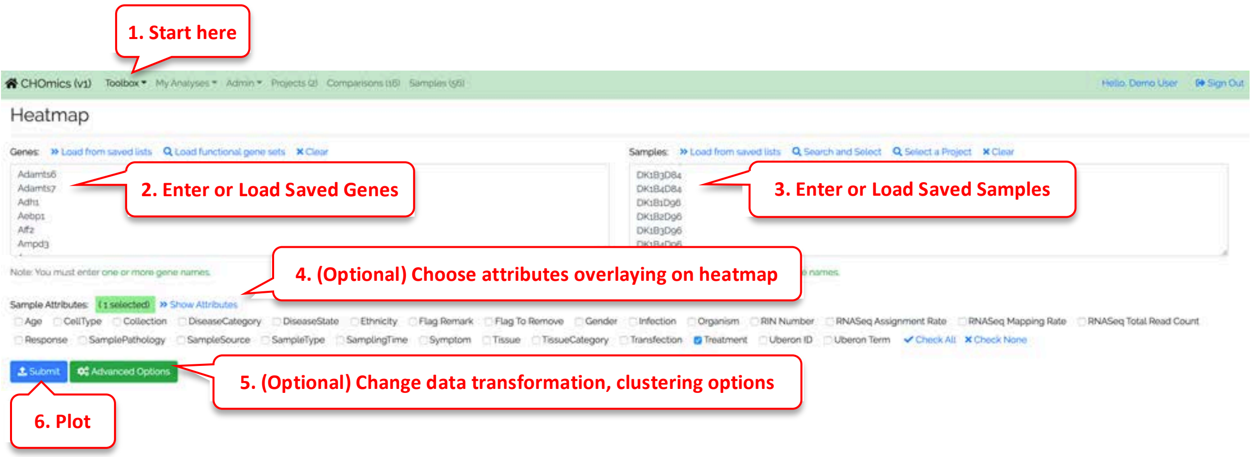

You can enter genes and samples in the box, or load pre-saved genes and samples quickly from your collection. Be default, we will log2 transform the gene expression data, perform scaling of the data across samples for each gene, and limit the scaled value to -3 to 3 before displaying the data in heatmap. This works well in most situations. However, advanced users can change the options. For example, if you want to keep the order of samples as you entered, just uncheck "Cluster Samples".

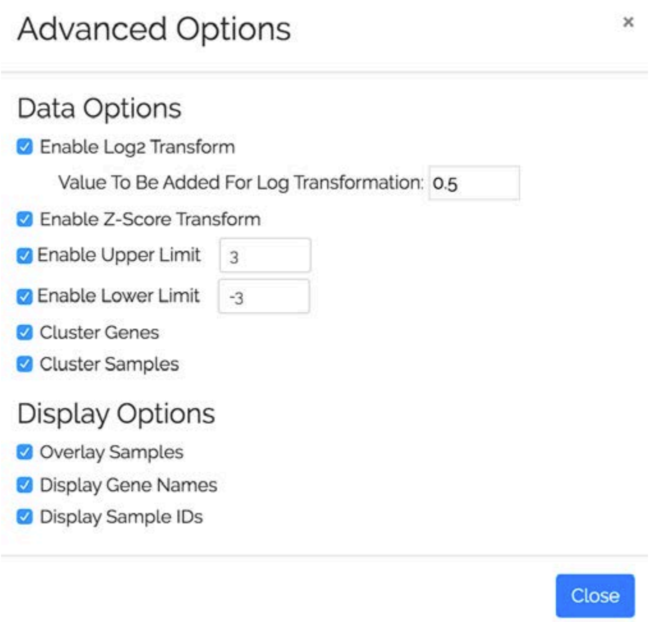

The heatmap is rendered by CanvassXpress. You can change the plot size if needed.

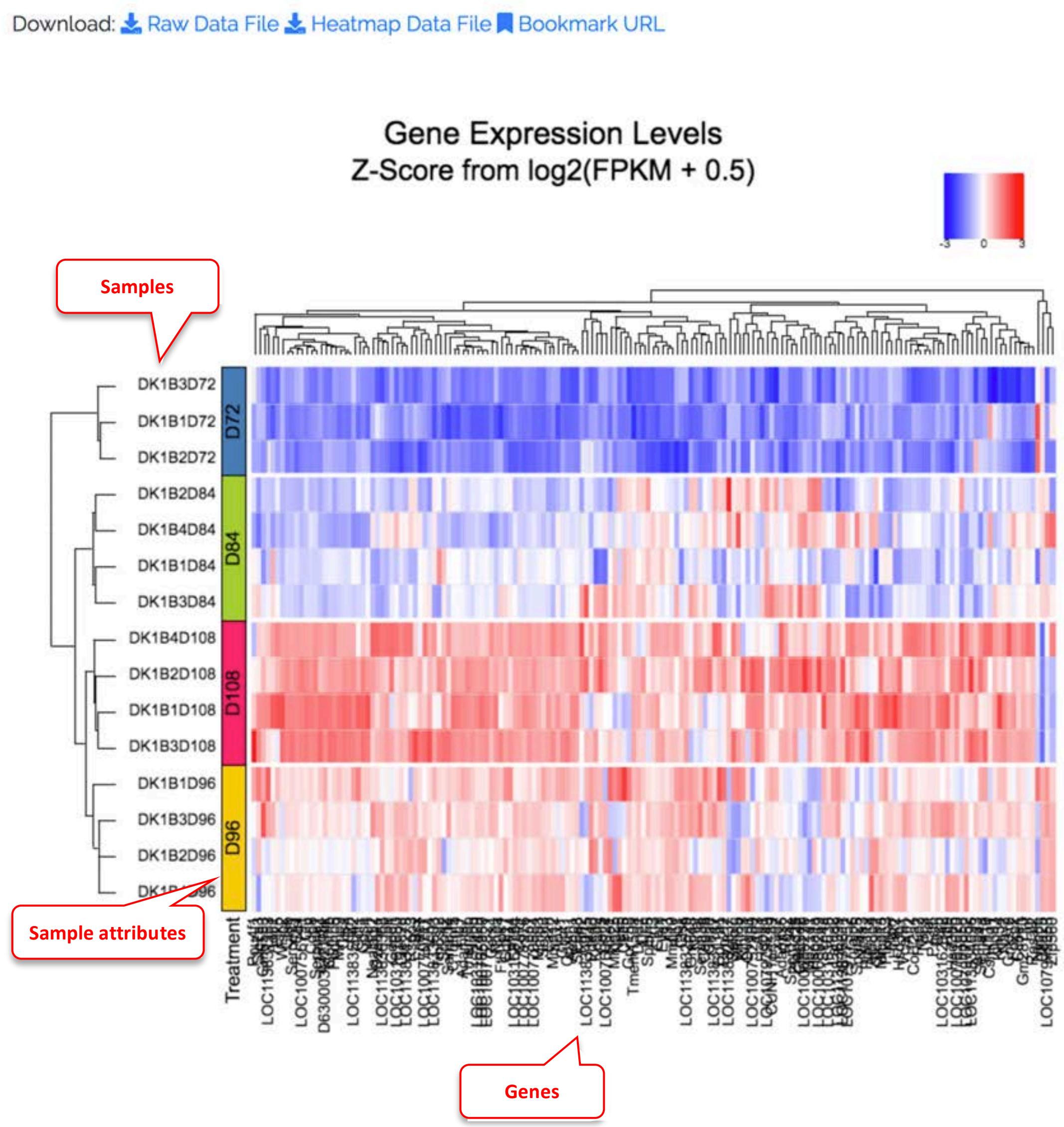

In the example heatmap, we entered a few significantly differential expressed genes between time D72 vs time D108. From heatmap clustering, we can see that the samples are clearly clustered by time points with increase of expression on most of genes along with time.

4.1.3 Multi-omics Expression View

Besides the plotting of transcriptomics data, CHOmics also enables the visualization of other types of omics data such as proteomics, and the comparison across omics.

Here is an example of comparing gene expression (transcriptomics) and protein expression (proteomics) of gene CTSA at different time points, using the ‘Gene Expression Plot’ tool. By righ clicking the plotting area, users can group the samples by different treatment time points while segregating the data by omics type(i.e, Samplesource).

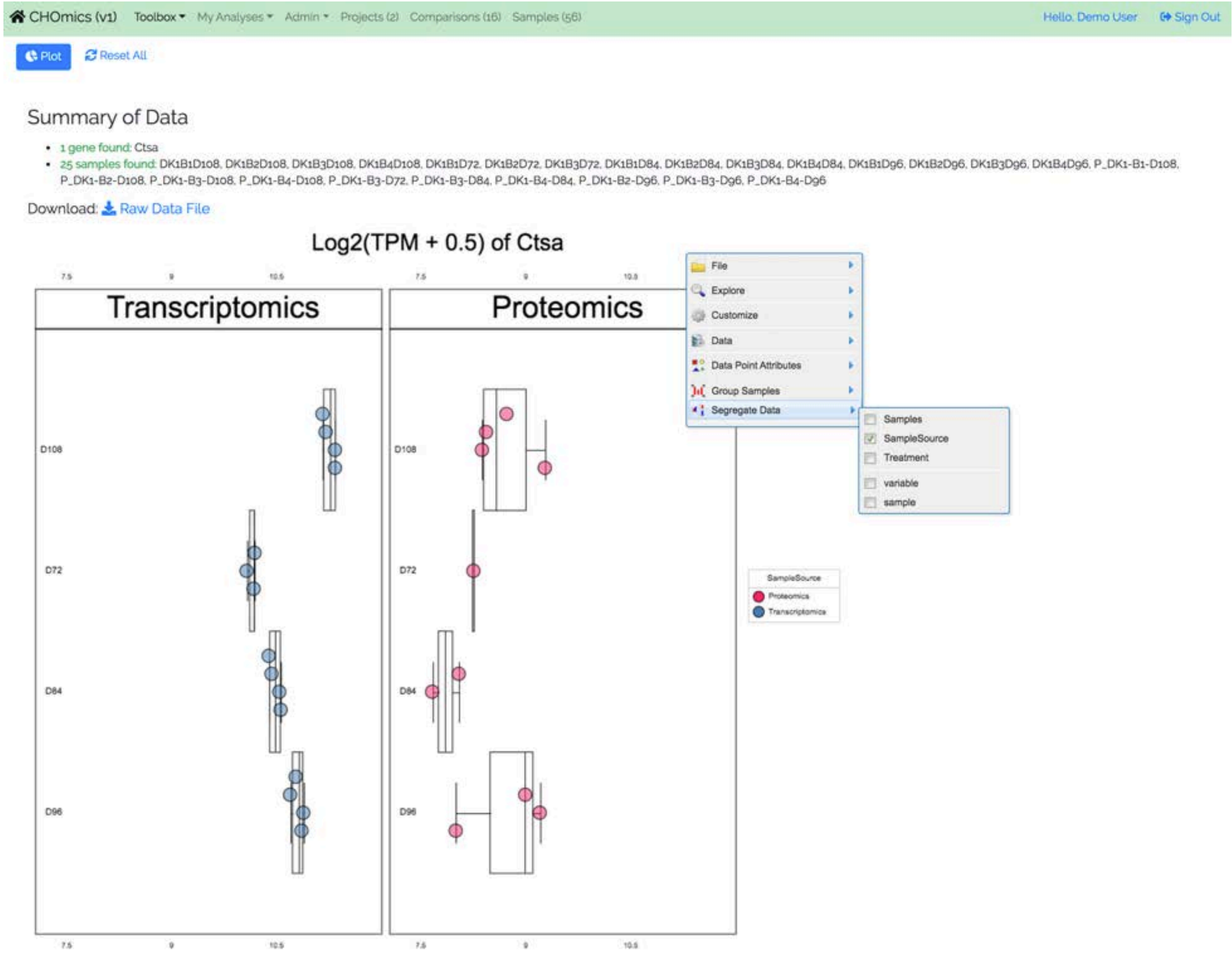

4.2 Visualize Comparison Data

4.2.1 Dashboard View of Comparison

The dashboard shows a summary of all the comparisons.

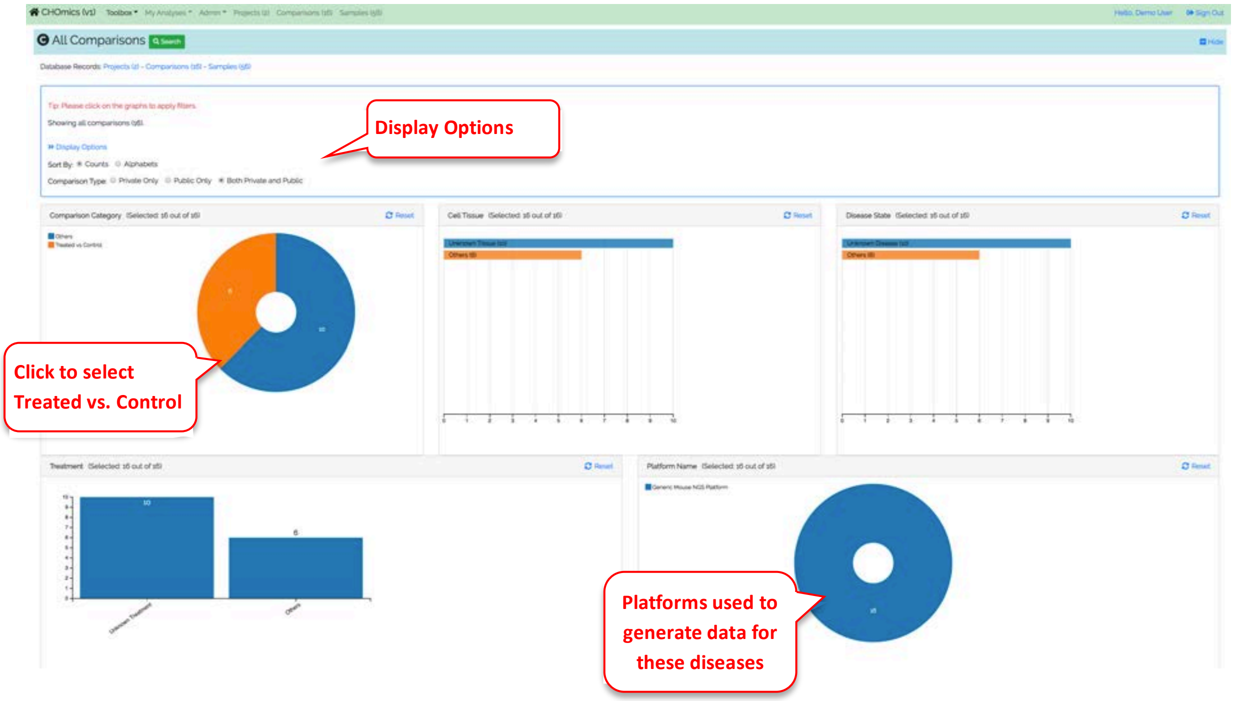

The above dashboard shows the comparisons from different Categories, Cell Type, Disease State, Treatment, Platform, etc. Below the dashboard, there is also a table listing all the comparisons.

In addition, users can set Dashboard Preference to change how the comparison summary is displayed.

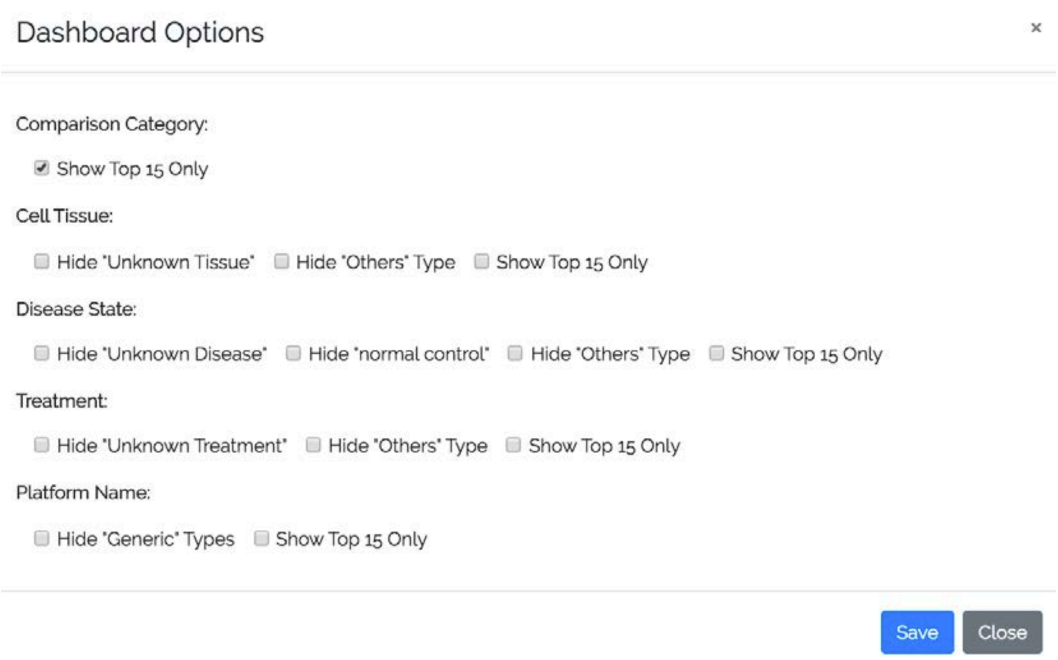

4.2.2 Bubble Plot

Bubble plot is another useful demonstration of gene or gene set in comparisons. For each gene, you can view all the available comparisons in a bubble chart.

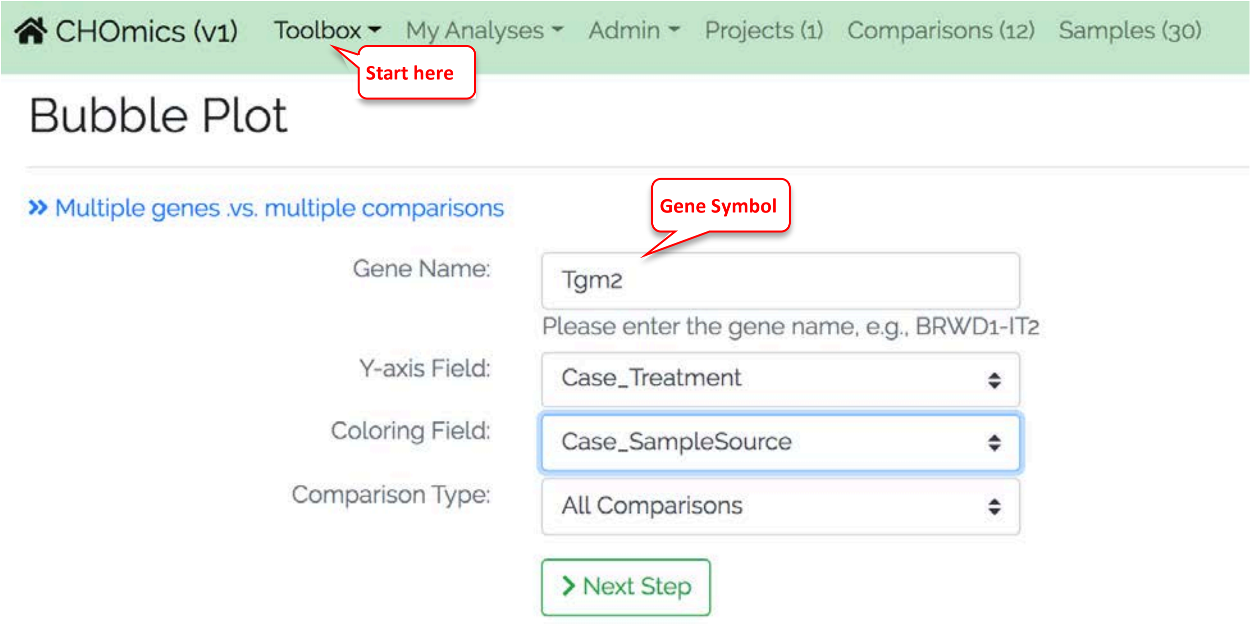

The default settings work for most users. After clicking the Next Step button, you will see a plot like:

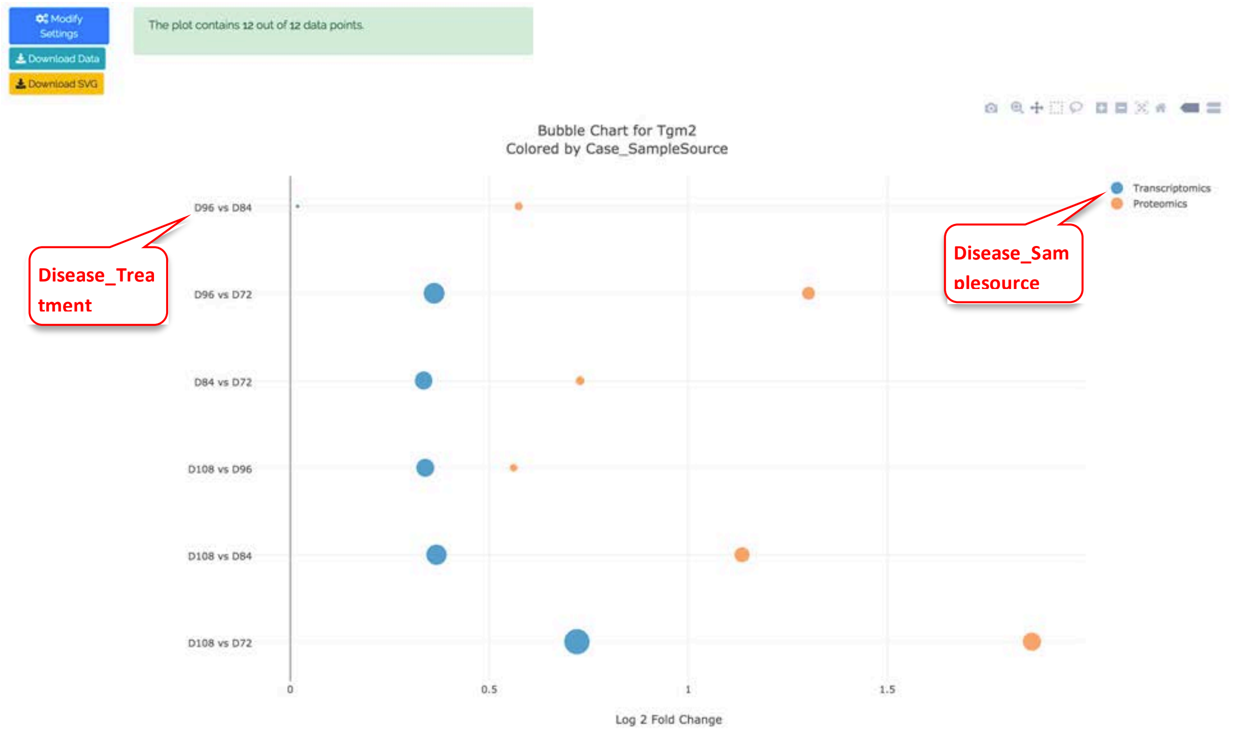

In the bubble plot, the X-axis shows log2 Fold Change of the comparison, the Y-axis shows 'Case_treatment'. Each dot represents the comparison result of this gene from one comparison. The color of the dot represent 'Case_Samplesource' (i.e, here we set as omics type), and the size of the dot represent significance (-log10(FDR), larger is more significant).

The user can click and unclick the color legend at right to select or deselect omics types. When mouse over a dot, more details are shown. And the user can also click the dot to link to other graphs.

The tool bars at top right corner allows the user to zoom and pan the graph.

The screenshot below shows the same bubble chart after selecting one omics type (i.e,transcriptomics), and zoom into a portion of the chart.

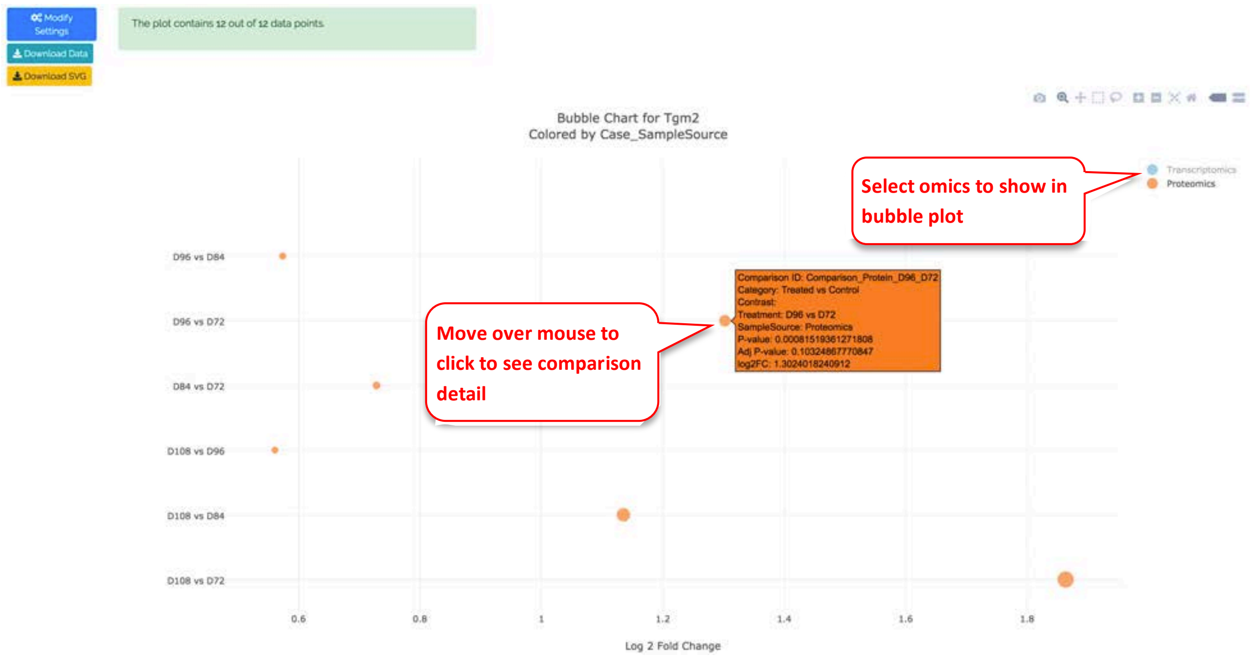

Data Filter and Advanced Settings in Bubble Plot

In addition, advanced users can change settings by click "Modify Settings Button". For example, the user may want to show a selected list of diseases. After clicking Customize in Case_Treatment, user can select which treatments to display in the pop-up window.

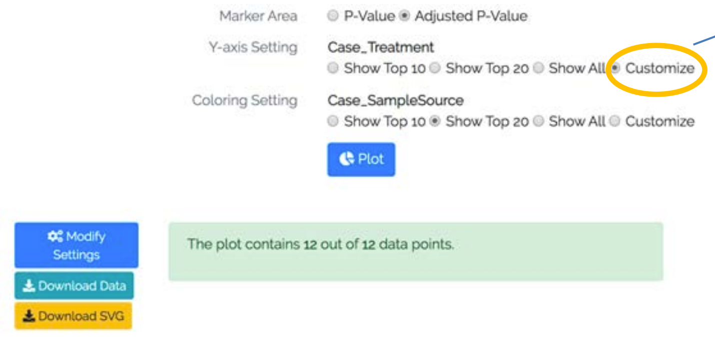

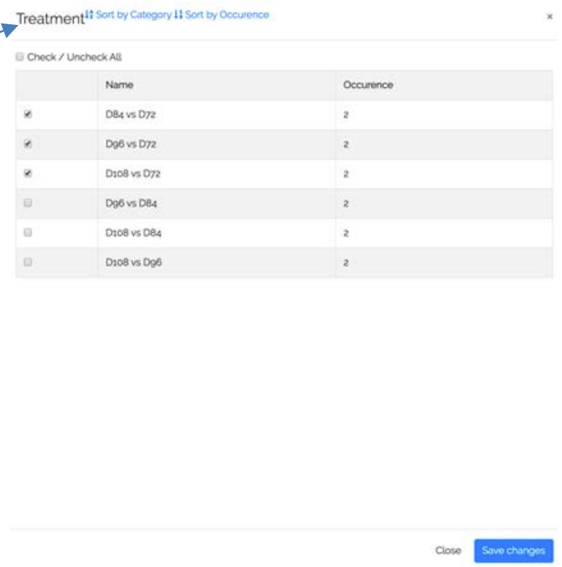

After modifying the setting, the user can click plot button to view the new chart. The system will display how many data points are chosen based on the filter.

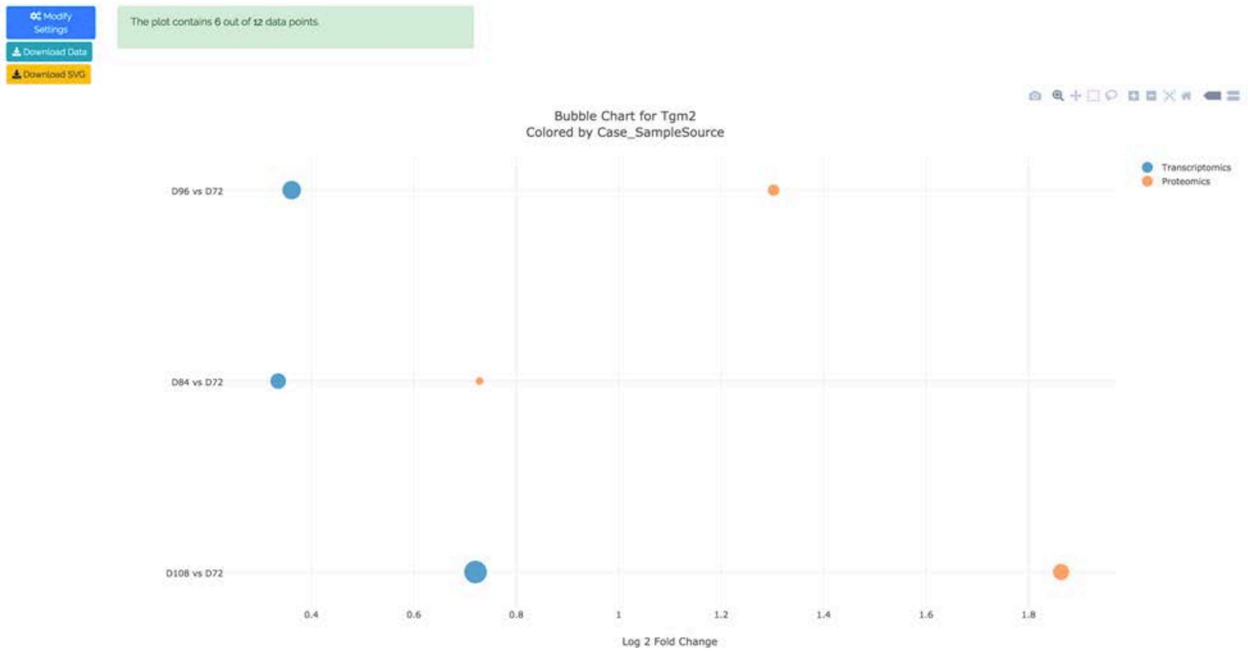

Bubble Plot of Multiple Genes and Multiple Comparisons

It can be useful to look at a set of genes (e.g. all differentially expressed genes, or genes from a certain pathways) in a set of related comparisons (e.g. all from the same disease).

To view this type of bubble plot, select the link for Multiple Genes vs. multiple comparisons.

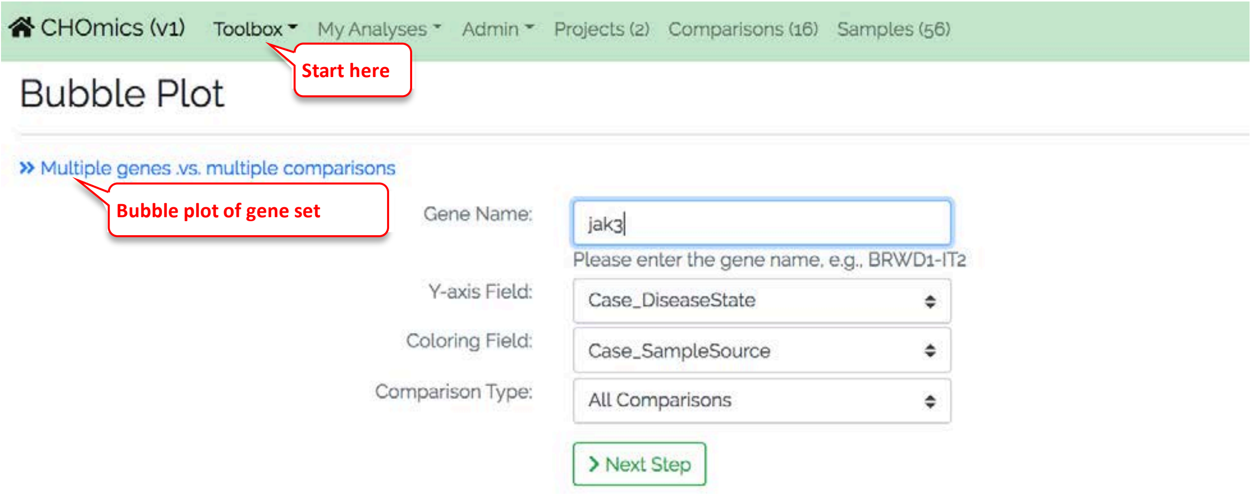

In the Genes and Comparisons Bubble plot window, you can now enter the symbols of the genes, and the comparison names. However, it is much easier to use the saved genes and saved comparisons features, or other tools from the system to quickly get a get set. Please see below for details.

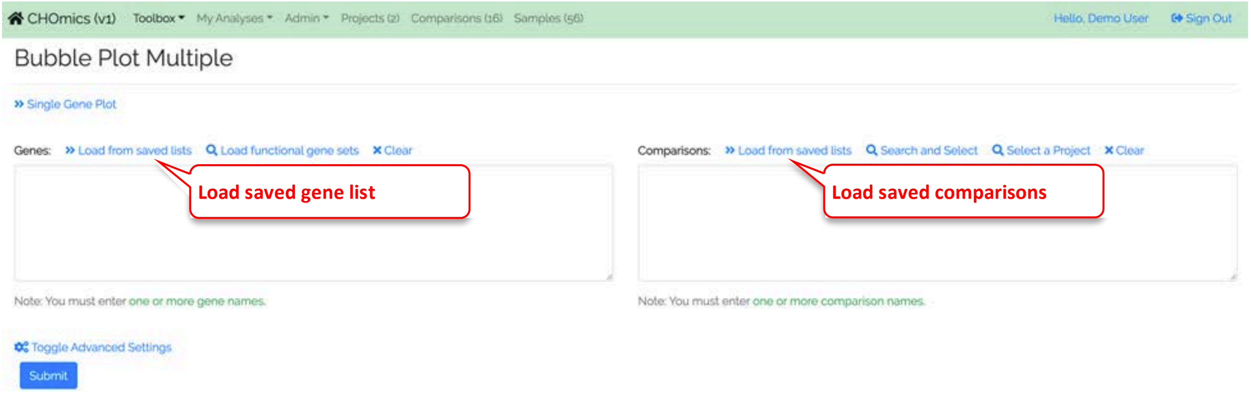

In the example below, we use dashboard to select 6 comparisons that are for different time points in CHO cell lines. We save the comparisons and load in the bubble plot tool. For gene list, we get the up-regulated genes from comparison D72 vs D108, and paste into the gene names fields.

In the bubble plot, the gene symbols are listed in Y-axis. The X-axis represents logFC, and color of the bubble represents comparison; the size of the bubble represents the significance.

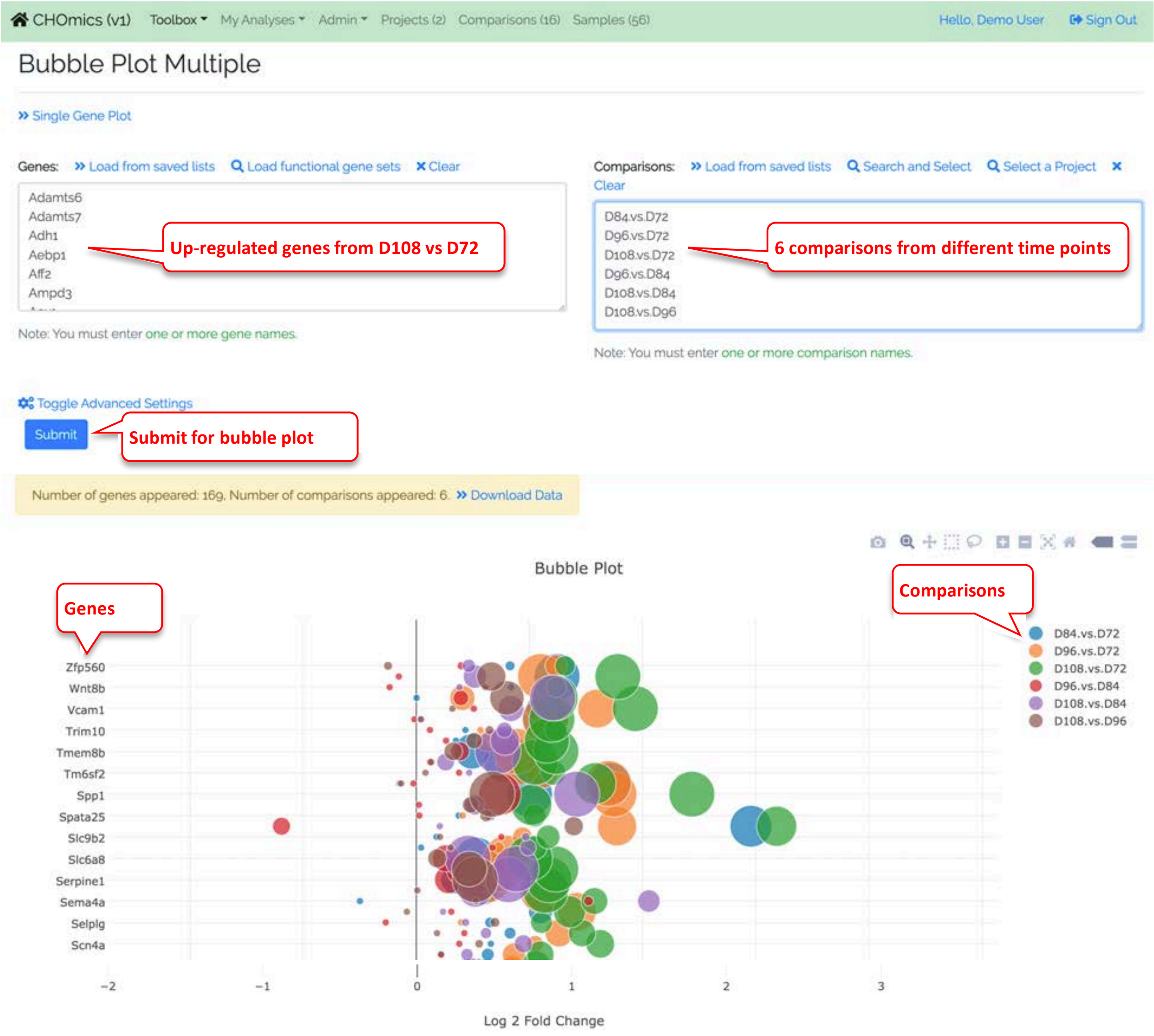

In the legend, the color keys for comparisons are shown. You can click the color key in the legend to hide/show comparisons. The size of the color dot in the legend correlates to the largest bubble for that comparison, which is the most significant gene with the smallest FDR.

Bubble Plot of Multiple Omics data

Similar to the bubble plot of multiple genes across multiple comparisons, users can further compare the genes on the comparisons from different omics data.

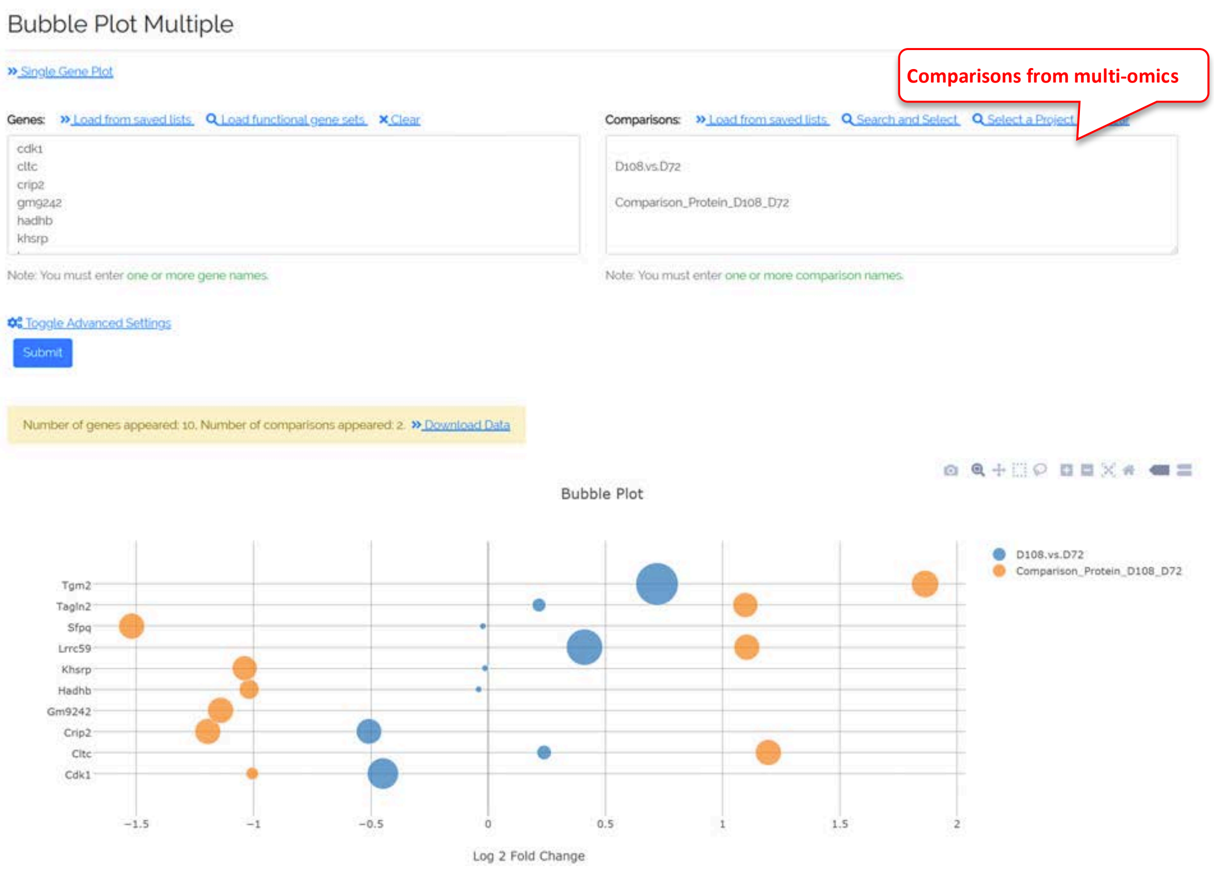

4.2.3 Get significant genes from comparisons

Another way to get a gene set to visualize in the genes/comparisons bubble plot is to filter for significantly changed genes. To do this, first select a few comparisons from the dash board, and click the "View Significantly Changed Genes" button.

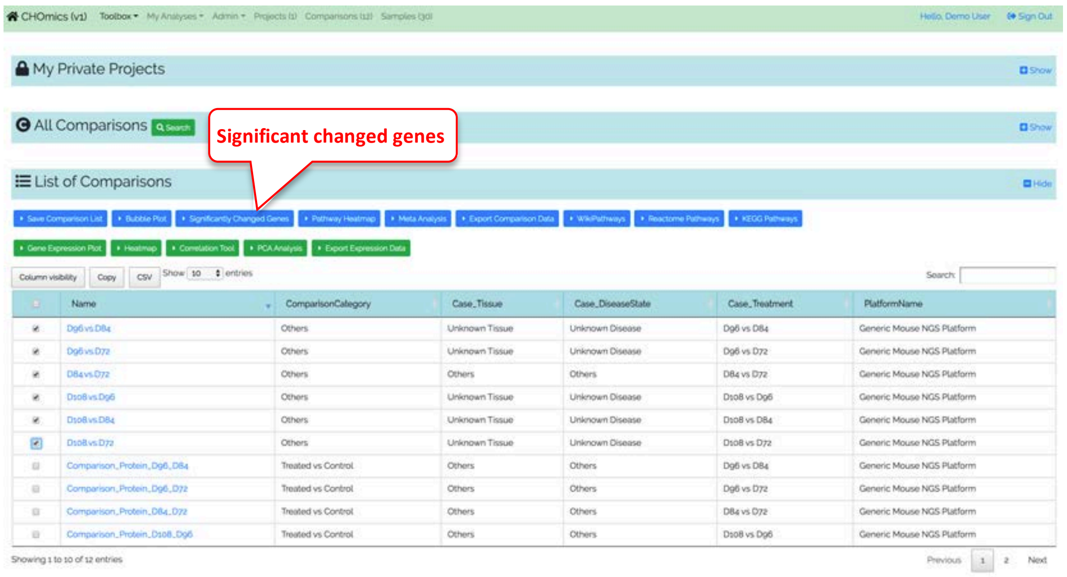

Dashboard filter.

In table, select comparisons and view significantly changed genes.

In the significantly Changed Genes window, the comparisons from the previous page are already loaded. You can add or remove comparisons if needed.

Now select direction (up-, down-, or both), and use the logFC cutoff and FDR value to get a list of genes. Depending on the comparisons, sometimes you may need to adjust the logFC and FDR values to get a good list of genes. In general, for bubble plot, using <100 genes will make the graph easier to read.

Once you are happy with the gene list, you can save it. You can also export the list for later use.

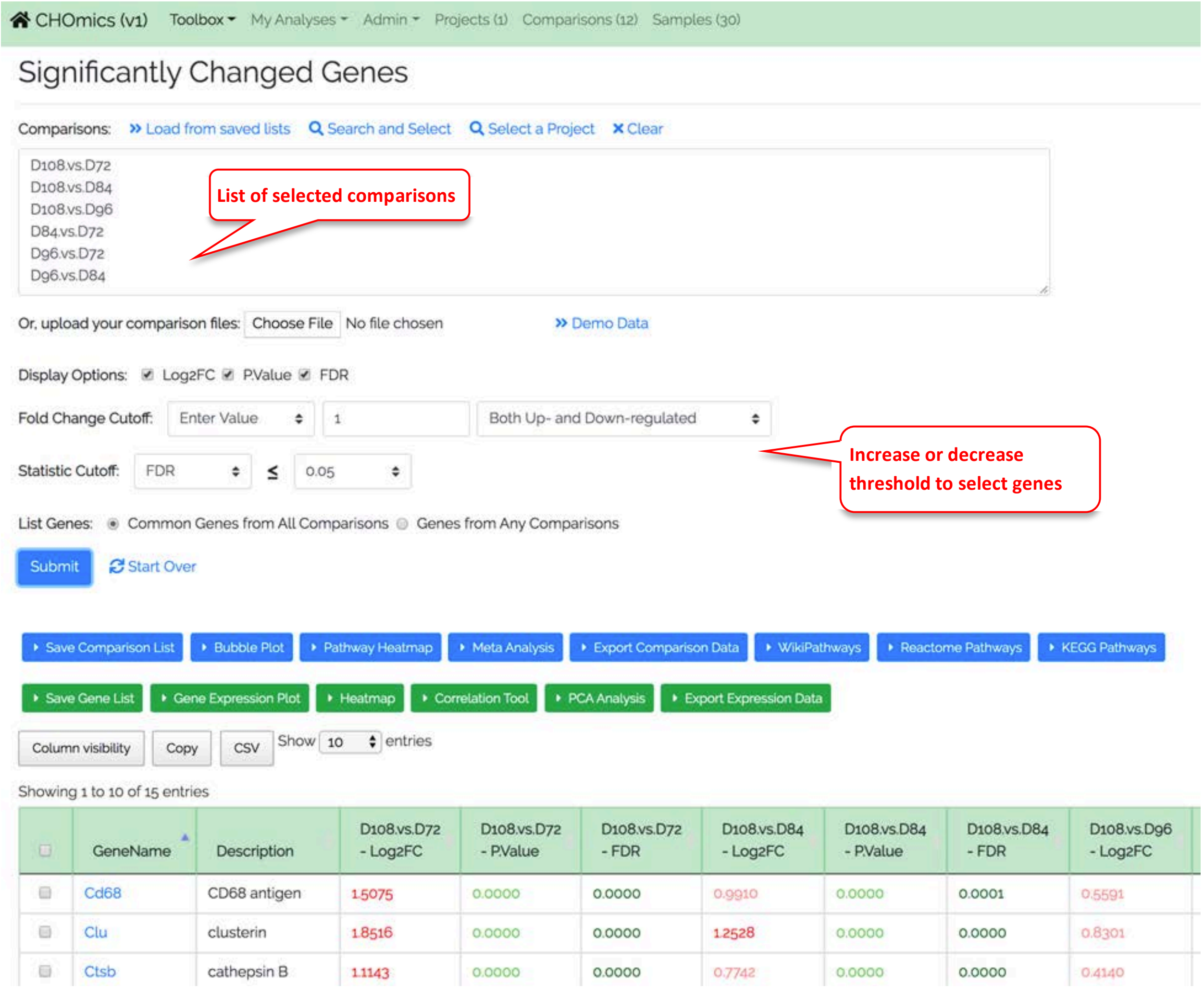

View Significantly Changed Genes in Bubble Plot

Back to the bubble plot, you can load the saved comparisons and saved genes and view the plot.

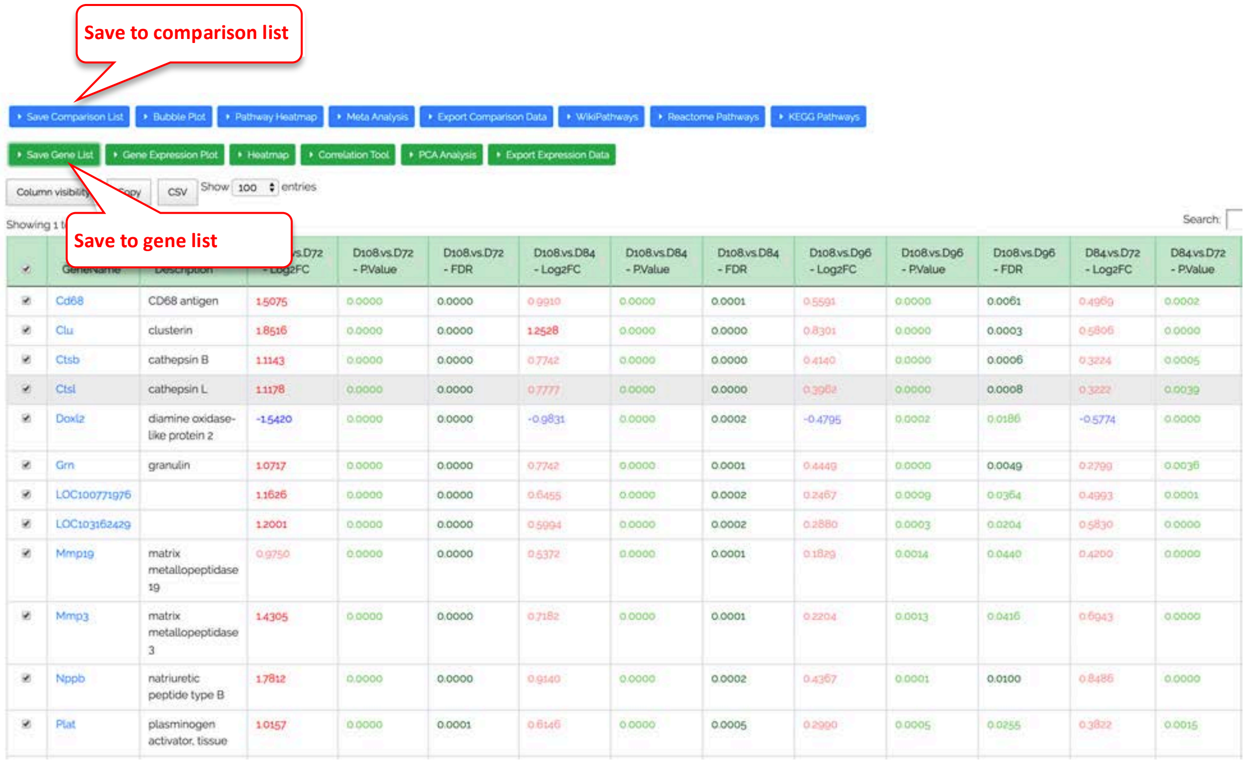

✔	GeneName	Description	[illegible]s.D72 - Log2FC	D108.vs.D72 - P.Value	D108.vs.D72 - FDR	D108.vs.D84 - Log2FC	D108.vs.D84 - P.Value	D108.vs.D84 - FDR	D108.vs.D96 - Log2FC	D108.vs.D96 - P.Value	D108.vs.D96 - FDR	D84.vs.D72 - Log2FC	D84.vs.D72 - P.Value
✔	Cd68	CD68 antigen	1.5075	0.0000	0.0000	0.9910	0.0000	0.0001	0.5591	0.0000	0.0061	0.4969	0.0002
✔	Clu	clusterin	1.8516	0.0000	0.0000	1.2528	0.0000	0.0000	0.8301	0.0000	0.0003	0.5806	0.0000
✔	Ctsb	cathepsin B	1.1143	0.0000	0.0000	0.7742	0.0000	0.0000	0.4140	0.0000	0.0006	0.3224	0.0005
✔	Ctsl	cathepsin L	1.1178	0.0000	0.0000	0.7777	0.0000	0.0000	0.3962	0.0000	0.0008	0.3222	0.0039
✔	Doxl2	diamine oxidase-like protein 2	-1.5420	0.0000	0.0000	-0.9831	0.0000	0.0002	-0.4795	0.0002	0.0186	-0.5774	0.0000
✔	Grn	granulin	1.0717	0.0000	0.0000	0.7742	0.0000	0.0001	0.4449	0.0000	0.0049	0.2799	0.0036
✔	LOC100771976		1.1626	0.0000	0.0000	0.6455	0.0000	0.0002	0.2467	0.0009	0.0364	0.4993	0.0001
✔	LOC103162429		1.2001	0.0000	0.0000	0.5994	0.0000	0.0002	0.2880	0.0003	0.0204	0.5830	0.0000
✔	Mmp19	matrix metallopeptidase 19	0.9750	0.0000	0.0000	0.5372	0.0000	0.0001	0.1829	0.0014	0.0440	0.4200	0.0000
✔	Mmp3	matrix metallopeptidase 3	1.4305	0.0000	0.0000	0.7182	0.0000	0.0001	0.2204	0.0013	0.0416	0.6943	0.0000
✔	Nppb	natriuretic peptide type B	1.7812	0.0000	0.0000	0.9140	0.0000	0.0002	0.4367	0.0001	0.0100	0.8486	0.0000
✔	Plat	plasminogen activator, tissue	1.0157	0.0000	0.0001	0.6146	0.0000	0.0005	0.2990	0.0005	0.0255	0.3822	0.0015

In the example below, it can be seen that most significant genes come from down-regulated direction from the first four comparisons.

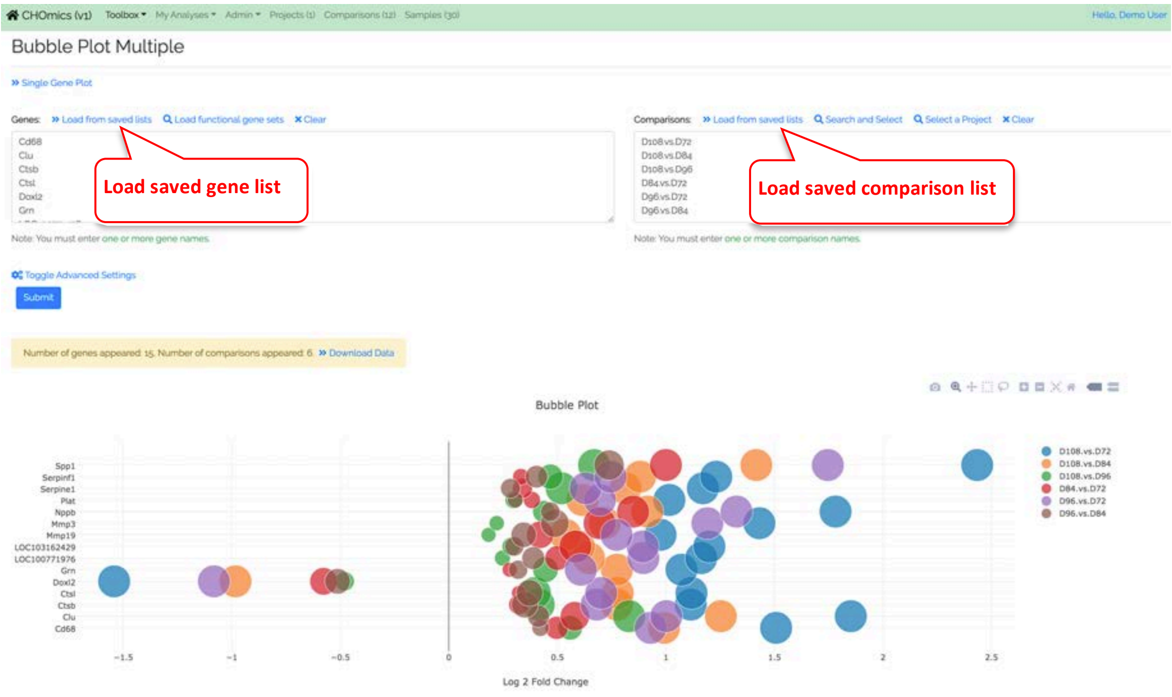

4.2.4 Volcano Plot

Volcano plot is useful to view a top level summary of how many genes are significantly up- or down-regulated in a comparison.

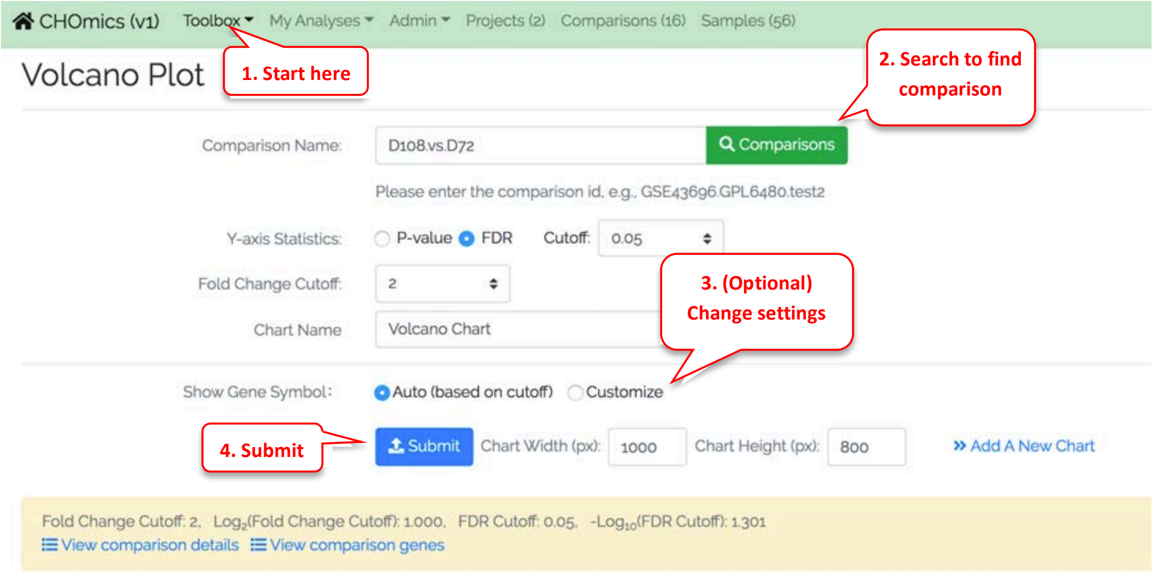

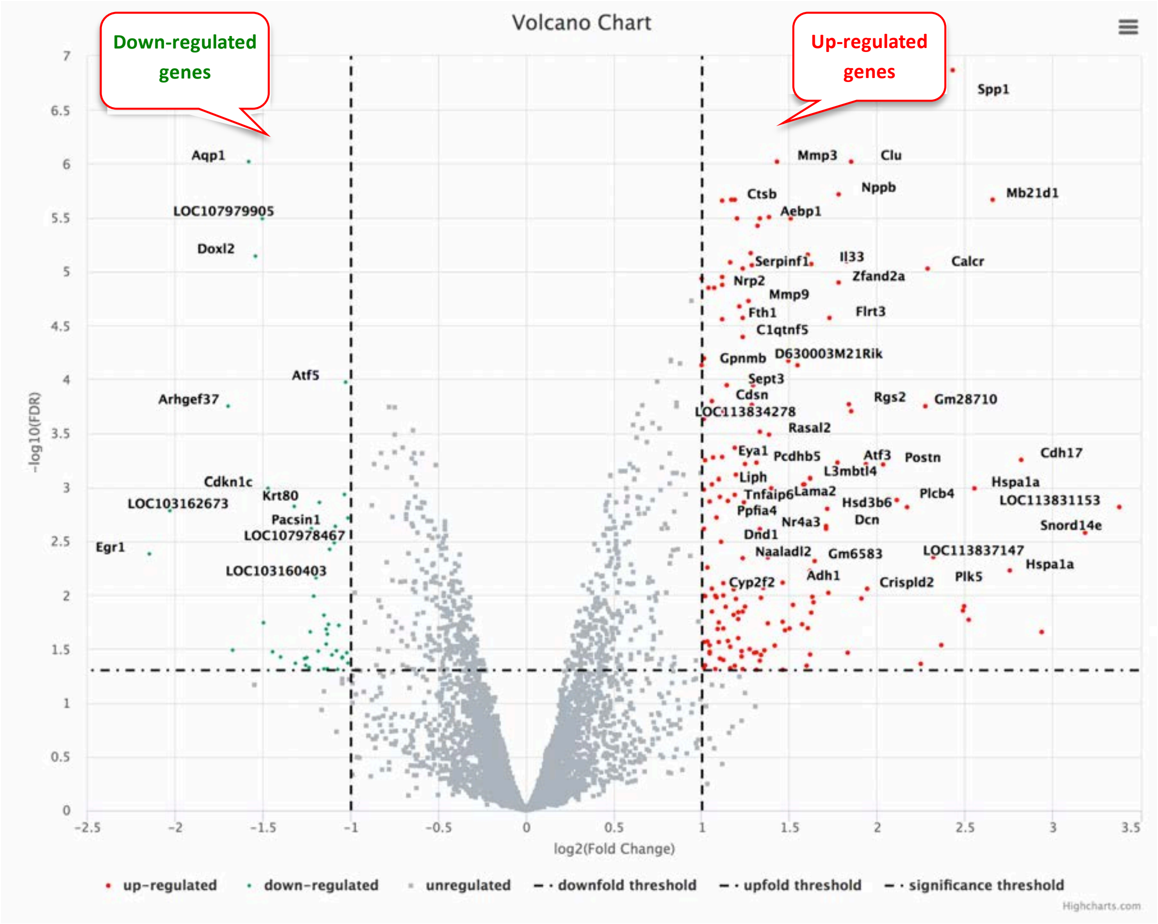

You can use mouse to drag over an area to zoom in.

Mouse over a point will show the gene details. Click the data point will show you links to other graphs.

View Multiple Volcano Plots Together

Users can also show multiple comparisons side-by-side. If needed, the user can also highlight the same group of genes across the volcano plots.

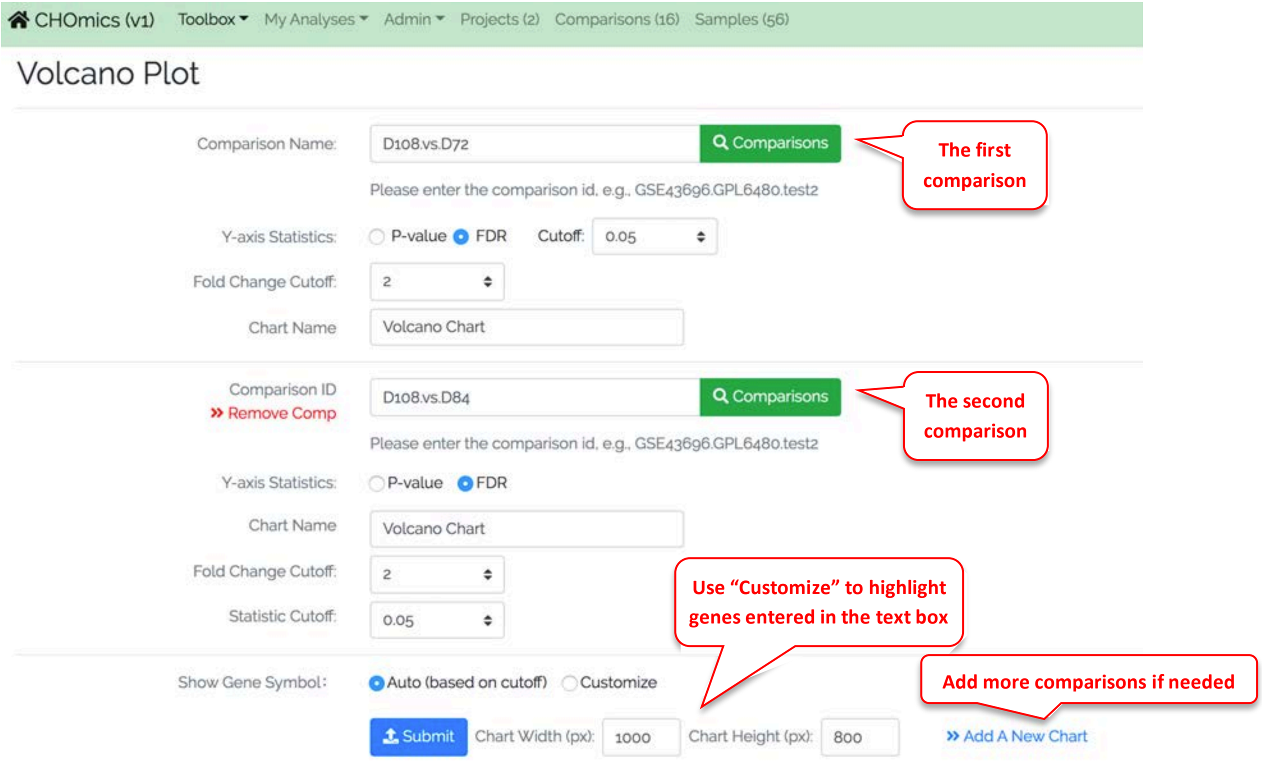

The resulting volcano plots are shown as below. Selected genes are shown as orange dots.

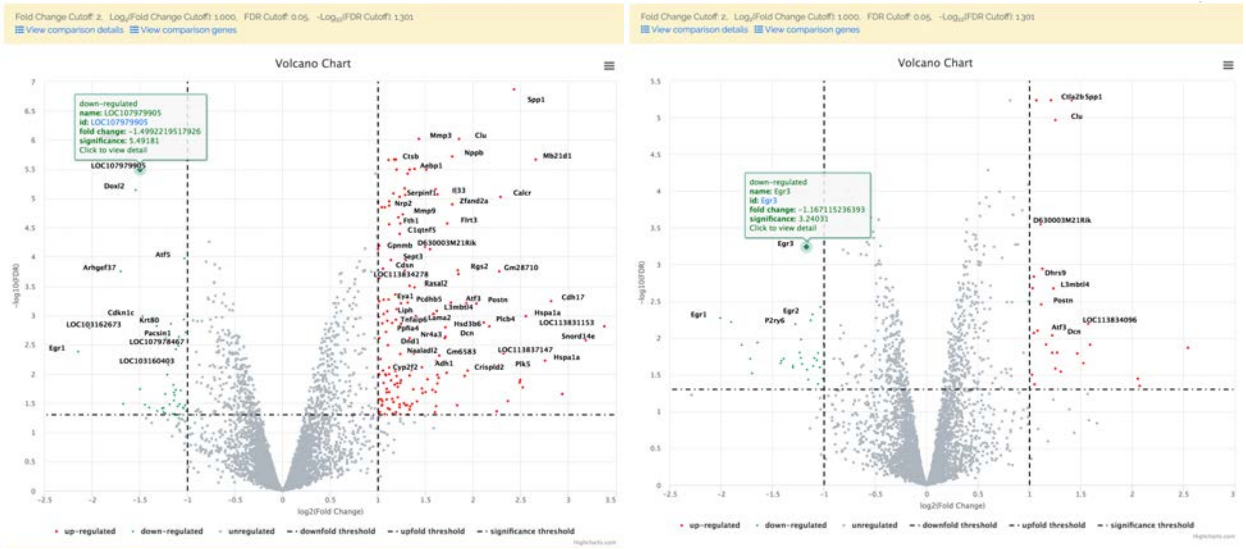

4.3 Visualize functional pathway

4.3.1 Enrichment from Up and Down Regulated Genes

When you view details of a comparison, the functional enrichment results are shown. Briefly, for each comparison, we generated the up- and down- regulate gene lists, and use these lists to compare with all genes in the genome to identify functions that are significantly enriched.

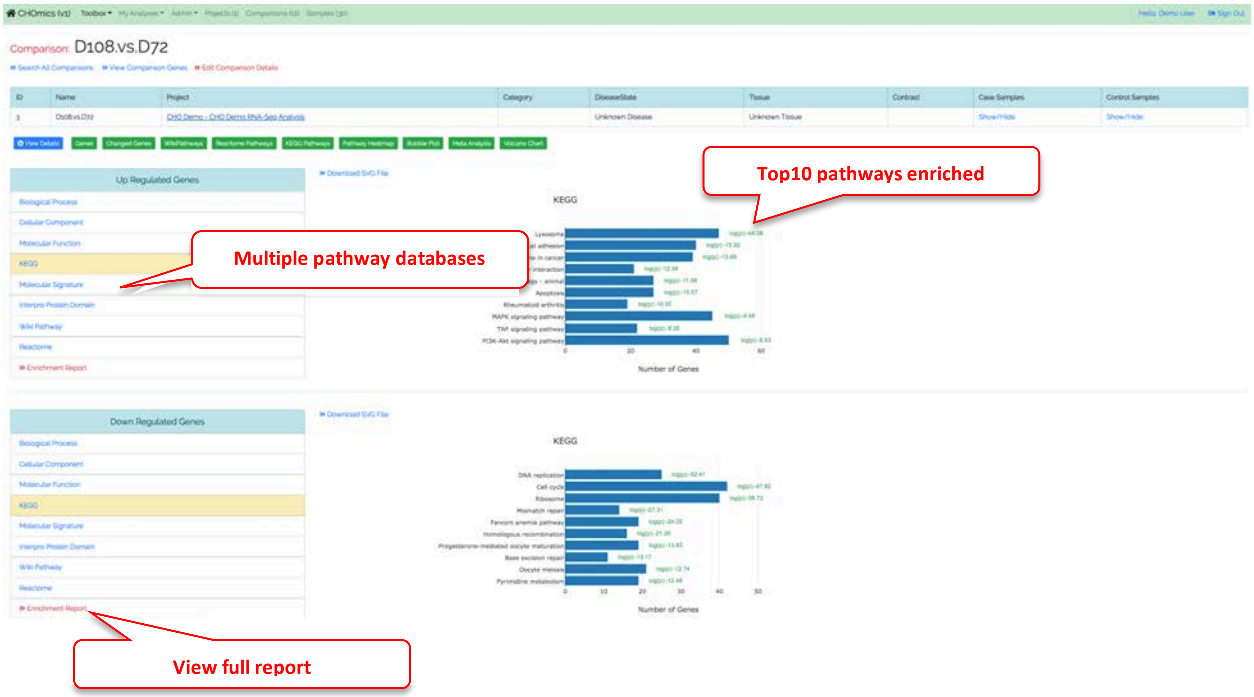

In the example above, this comparison is between D108 vs D72, and the top up-regulated biological processed are response to virus, immune effector process.

Click the left menu will switch the bar charts for different categories (Gene Ontology, KEGG, Molecular signature, Protein domain etc).

The bar charts here show the top 10 categories. To view complete results, click the Enrichment Report.

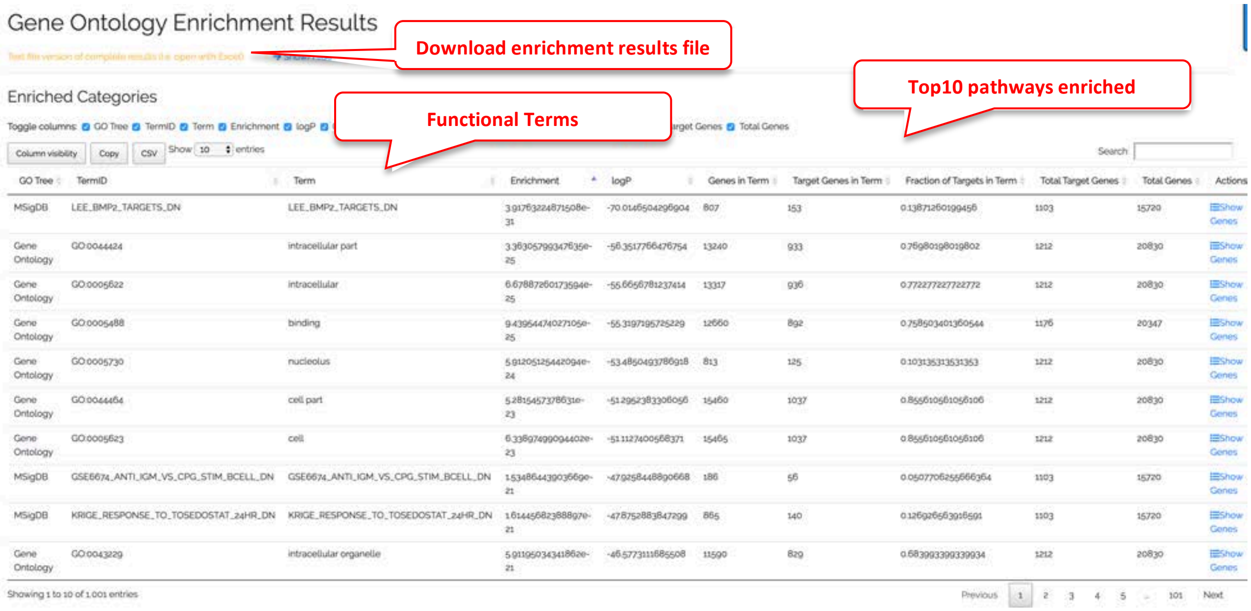

In the enrichment report, the full list of functional terms are shown by order of p-value.

4.3.2 View Changed Genes from a Functional Term in Volcano Plot

From the bar chat, click a functional term, and you have the option to view these genes in a volcano plot.

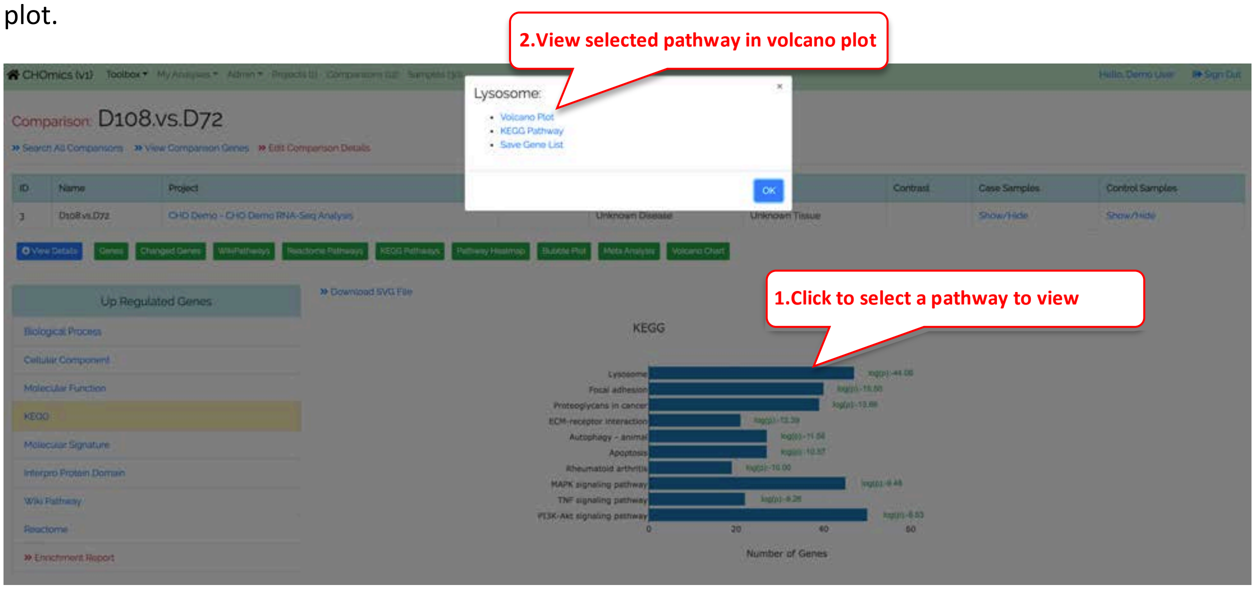

Once you click the link in the popup window, volcano plot will be generated for the comparison with the changed genes from the selected term highlighted.

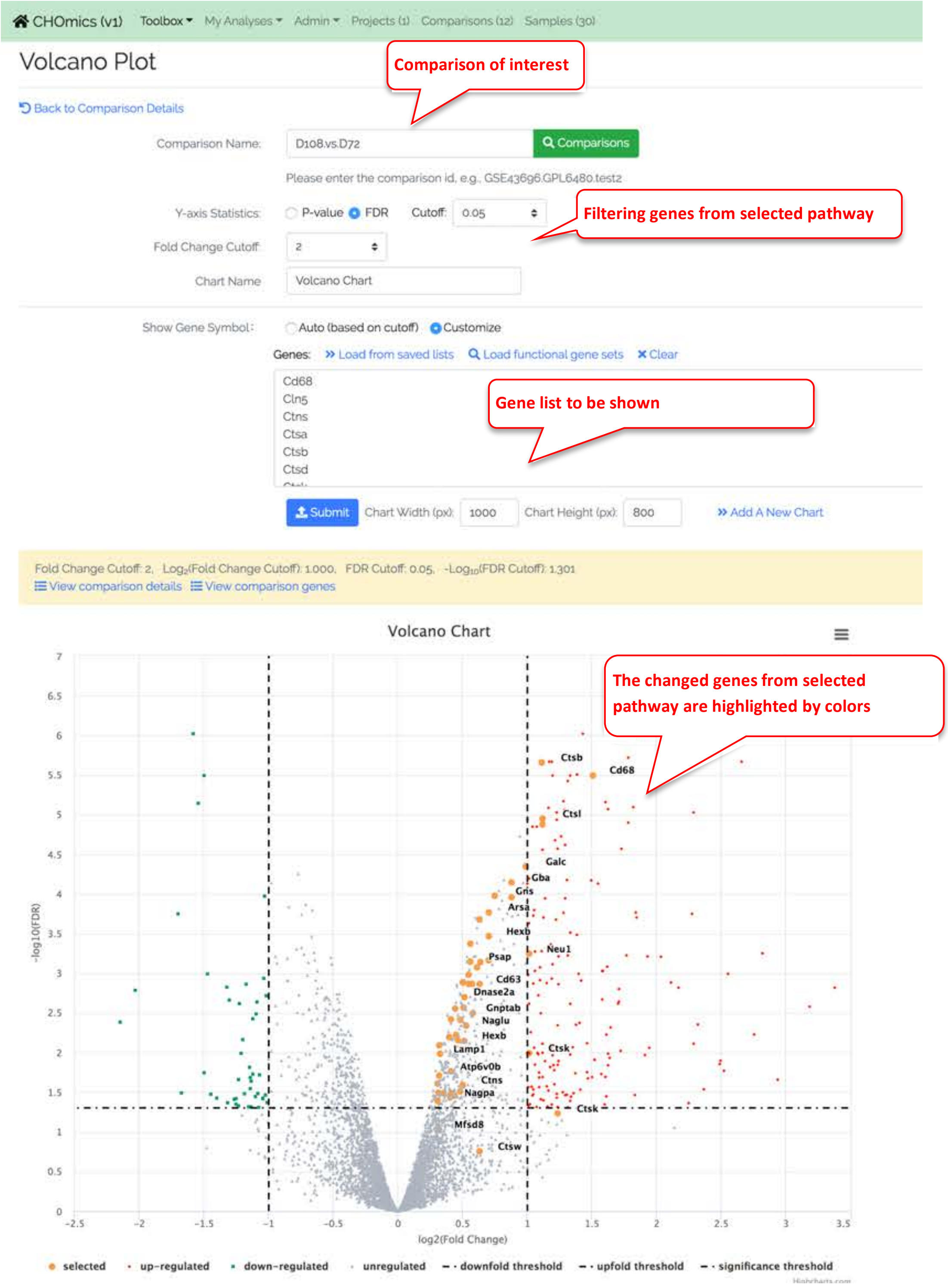
CHOmics (v1)
Toolbox
My Analyses
Admin
Projects (1)
Comparisons (12)
Samples (30)
Volcano Plot
Comparison of interest
Back to Comparison Details
Comparison Name:
D108.vs.D72
Comparisons
Please enter the comparison id, e.g., GSE43696.GPL6480.test2
Y-axis Statistics:
P-value
FDR
Cutoff:
0.05
Filtering genes from selected pathway
Fold Change Cutoff:
2
Chart Name
Volcano Chart
Show Gene Symbol:
Auto (based on cutoff)
Customize
Genes:
Load from saved lists
Load functional gene sets
Clear
Cd68
Cln5
Ctns
Ctsa
Ctsb
Ctsd
Gene list to be shown
Submit
Chart Width (px):
1000
Chart Height (px):
800
Add A New Chart
Fold Change Cutoff: 2, Log2(Fold Change Cutoff): 1.000, FDR Cutoff: 0.05, -Log10(FDR Cutoff): 1.301
View comparison details
View comparison genes
Volcano Chart
The changed genes from selected pathway are highlighted by colors
Ctsb
Cd68
Ctsl
Galc
Gba
Gns
Arsa
Hexb
Neu1
Psap
Cd63
Dnase2a
Gnptab
Naglu
Hexb
Lamp1
Ctsk
Atp6v0b
Ctns
Nagpa
Ctsk
Mfsd8
Ctsw
-log10(FDR)
log2(Fold Change)
selected
up-regulated
down-regulated
unregulated
downfold threshold
upfold threshold
significance threshold

4.3.3 View Enriched Pathways Directly from Comparison Details

From the bar chat, if you are viewing KEGG or wikipathway database, clicking the pathway name and you have the option to view pathway plot.

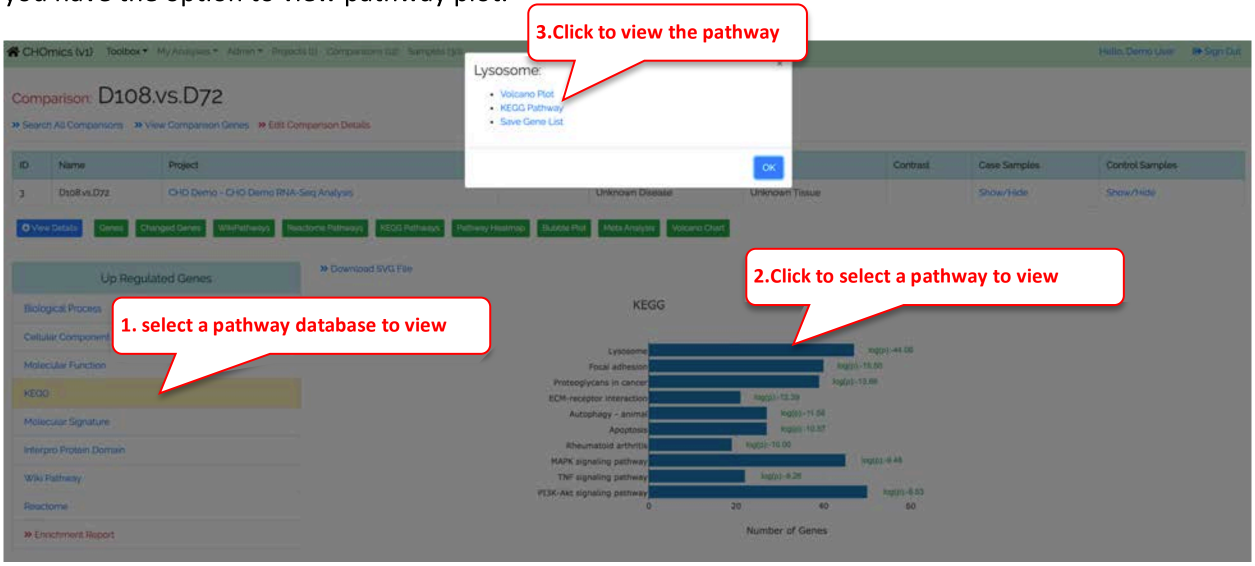

This will automatically open the pathway visualization page, and preload the pathway and comparison. Click submit to view the pathway.

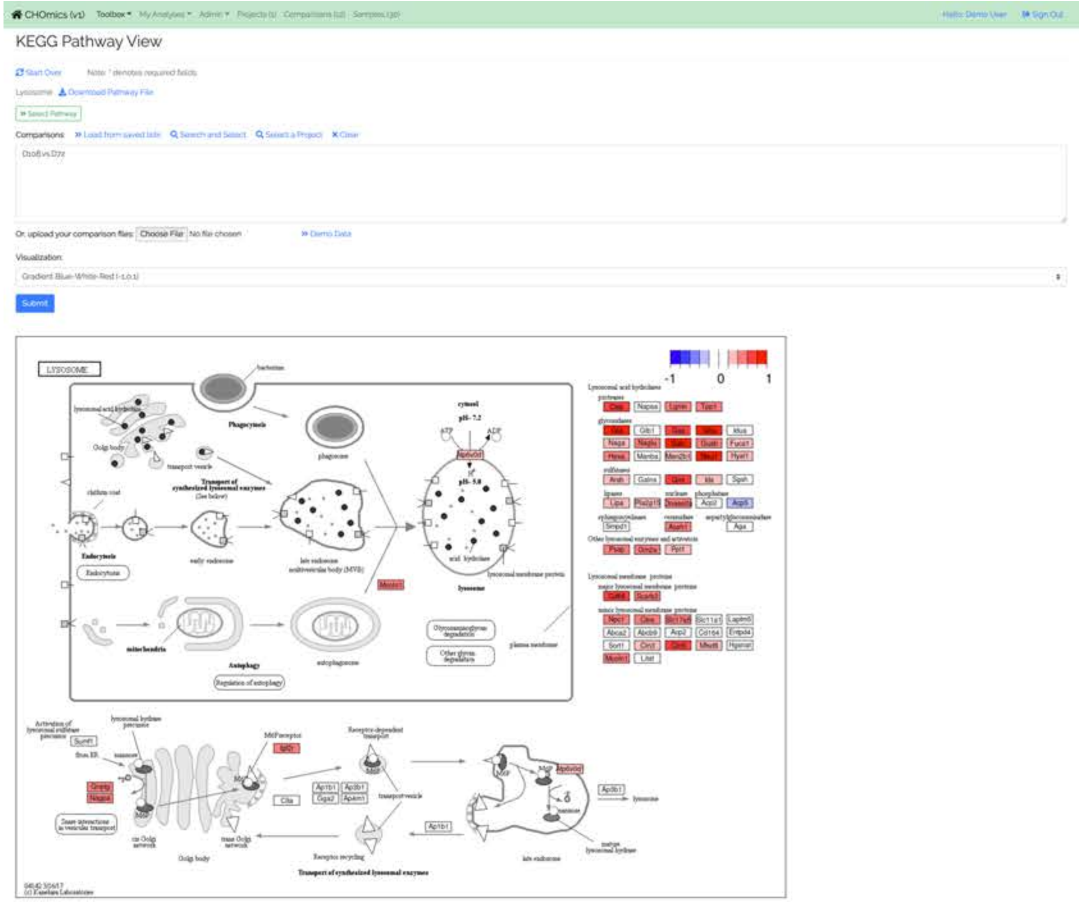

Gene Set Enrichment from Ranked Genes

For each comparison, we produce a rank file for all genes using logFC. We use PAGE (Parametric Analysis of Gene Set Enrichment) to identify significant biological changes. PAGE can be more sensitive for comparisons where the logFC is relatively small, but most genes in a functional set show the same direction of change.

The predefined gene sets were from MSigDB.

For each comparison, the top up-regulated and down-regulated gene sets are plotted.

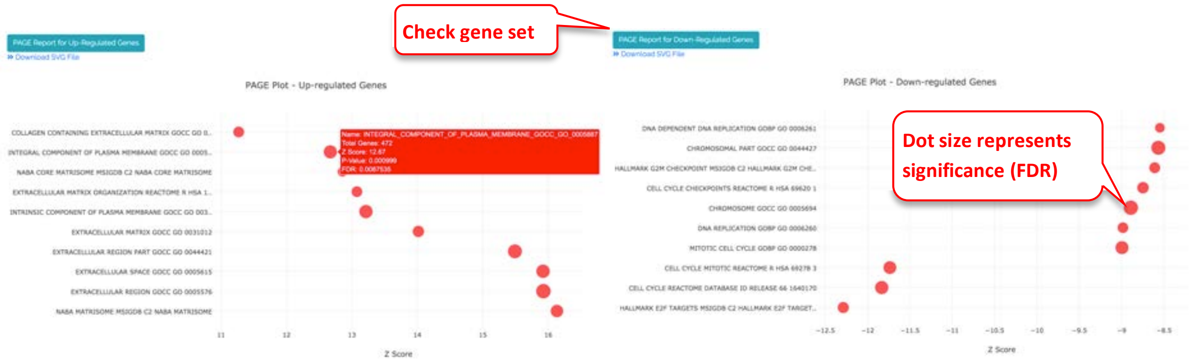

To view the full list of gene sets, you can click the report for genes as shown in following figure.

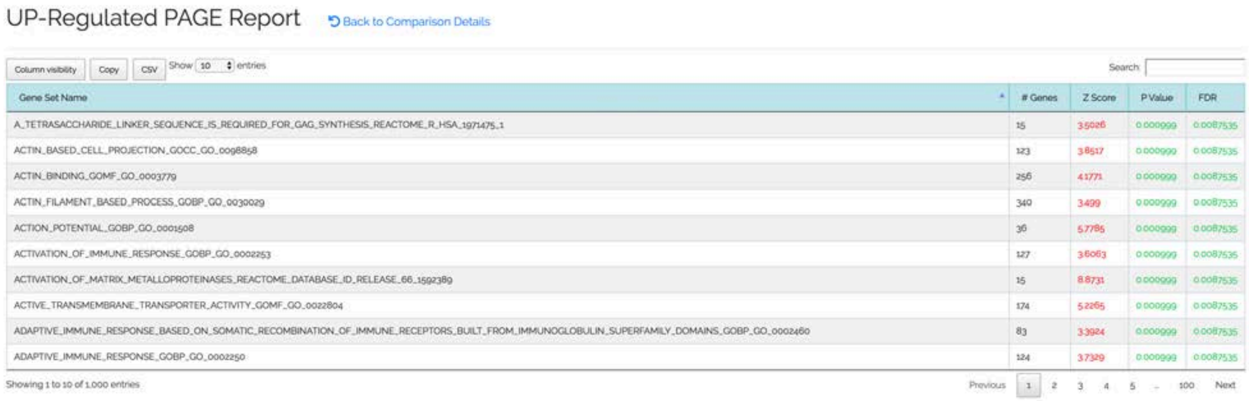

UP-Regulated PAGE Report   Back to Comparison Details

Column visibility | Copy | CSV | Show 10 entries   Search:

Gene Set Name	# Genes	Z Score	P Value	FDR
A_TETRASACCHARIDE_LINKER_SEQUENCE_IS_REQUIRED_FOR_GAG_SYNTHESIS_REACTOME_R_HSA_1971475_1	15	3.5026	0.000999	0.0087535
ACTIN_BASED_CELL_PROJECTION_GOCC_GO_0098858	123	3.8517	0.000999	0.0087535
ACTIN_BINDING_GOMF_GO_0003779	256	4.1771	0.000999	0.0087535
ACTIN_FILAMENT_BASED_PROCESS_GOBP_GO_0030029	340	3.499	0.000999	0.0087535
ACTION_POTENTIAL_GOBP_GO_0001508	36	5.7785	0.000999	0.0087535
ACTIVATION_OF_IMMUNE_RESPONSE_GOBP_GO_0002253	127	3.6063	0.000999	0.0087535
ACTIVATION_OF_MATRIX_METALLOPROTEINASES_REACTOME_DATABASE_ID_RELEASE_66_1592389	15	8.8731	0.000999	0.0087535
ACTIVE_TRANSMEMBRANE_TRANSPORTER_ACTIVITY_GOMF_GO_0022804	174	5.2265	0.000999	0.0087535
ADAPTIVE_IMMUNE_RESPONSE_BASED_ON_SOMATIC_RECOMBINATION_OF_IMMUNE_RECEPTORS_BUILT_FROM_IMMUNOGLOBULIN_SUPERFAMILY_DOMAINS_GOBP_GO_0002460	83	3.3924	0.000999	0.0087535
ADAPTIVE_IMMUNE_RESPONSE_GOBP_GO_0002250	124	3.7329	0.000999	0.0087535

Showing 1 to 10 of 1,000 entries   Previous 1 2 3 4 5 … 100 Next

4.3.4 Multi-layer visualization

If you are interested in a particular pathway, sometimes it is useful to map the RNA-Seq or microarray data to the pathway for visualization.

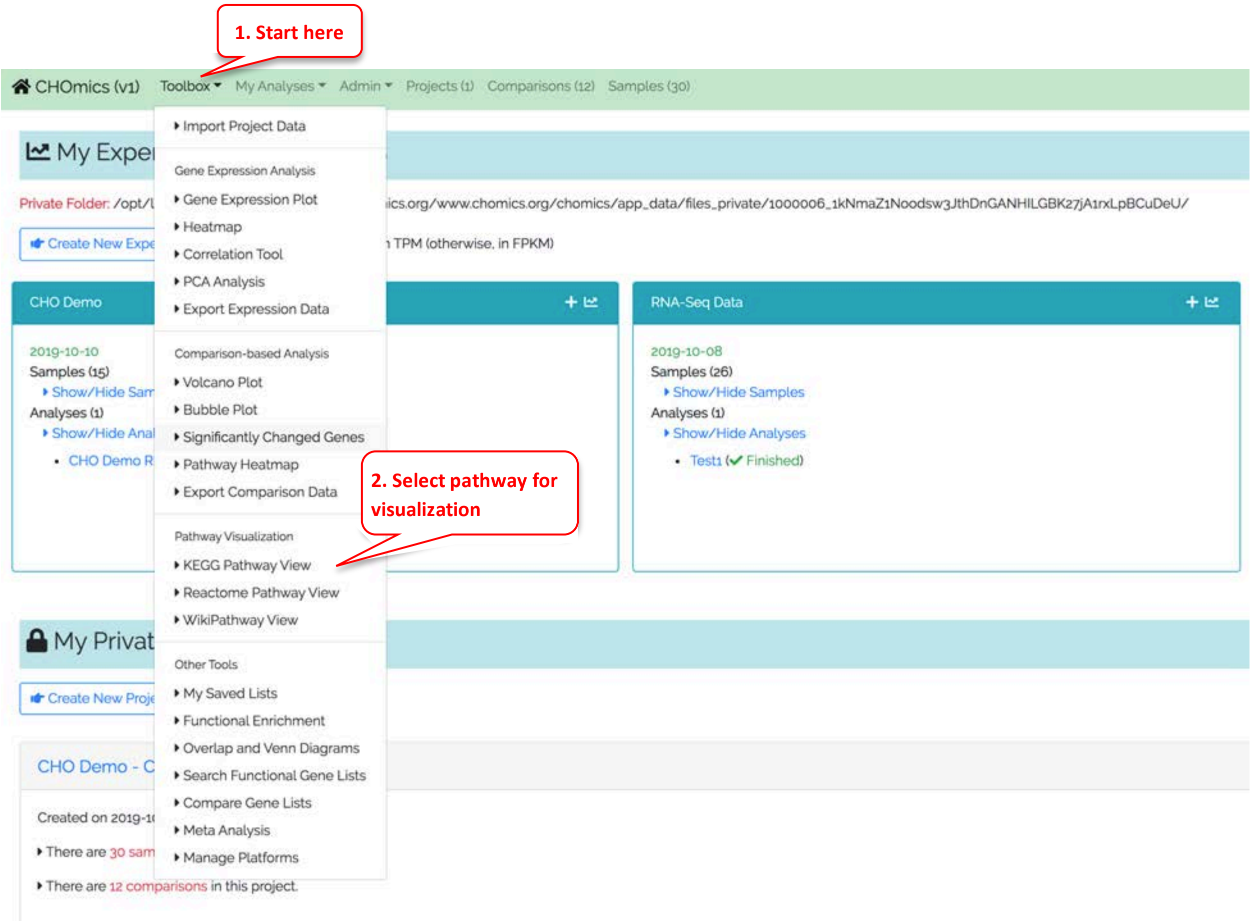
1. Start here
2. Select pathway for visualization
CHOmics (v1)
Toolbox
My Analyses
Admin
Projects (1)
Comparisons (12)
Samples (30)
Import Project Data
Gene Expression Analysis
Gene Expression Plot
Heatmap
Correlation Tool
PCA Analysis
Export Expression Data
Comparison-based Analysis
Volcano Plot
Bubble Plot
Significantly Changed Genes
Pathway Heatmap
Export Comparison Data
Pathway Visualization
KEGG Pathway View
Reactome Pathway View
WikiPathway View
Other Tools
My Saved Lists
Functional Enrichment
Overlap and Venn Diagrams
Search Functional Gene Lists
Compare Gene Lists
Meta Analysis
Manage Platforms
CHO Demo
RNA-Seq Data
2019-10-08
Samples (26)
Show/Hide Samples
Analyses (1)
Show/Hide Analyses
Test1 (Finished)
There are 12 comparisons in this project.

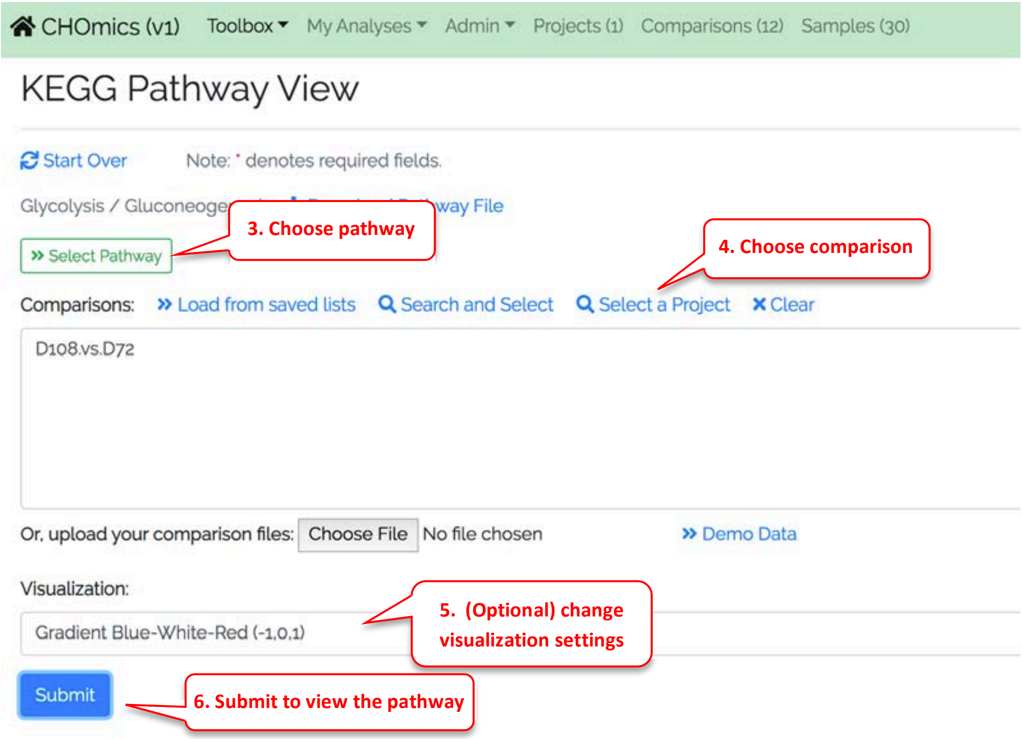
CHOmics (v1)
KEGG Pathway View
Start Over
Note: * denotes required fields.
3. Choose pathway
Select Pathway
4. Choose comparison
Comparisons:
Load from saved lists
Search and Select
Select a Project
Clear
D108.vs.D72
Or, upload your comparison files:
Choose File
No file chosen
Demo Data
Visualization:
Gradient Blue-White-Red (-1,0,1)
5. (Optional) change visualization settings
Submit
6. Submit to view the pathway

In the pathway plot, typically we use red-blue color scale to show the log2 Fold Change. Blue is down-regulated, red is up-regulated.

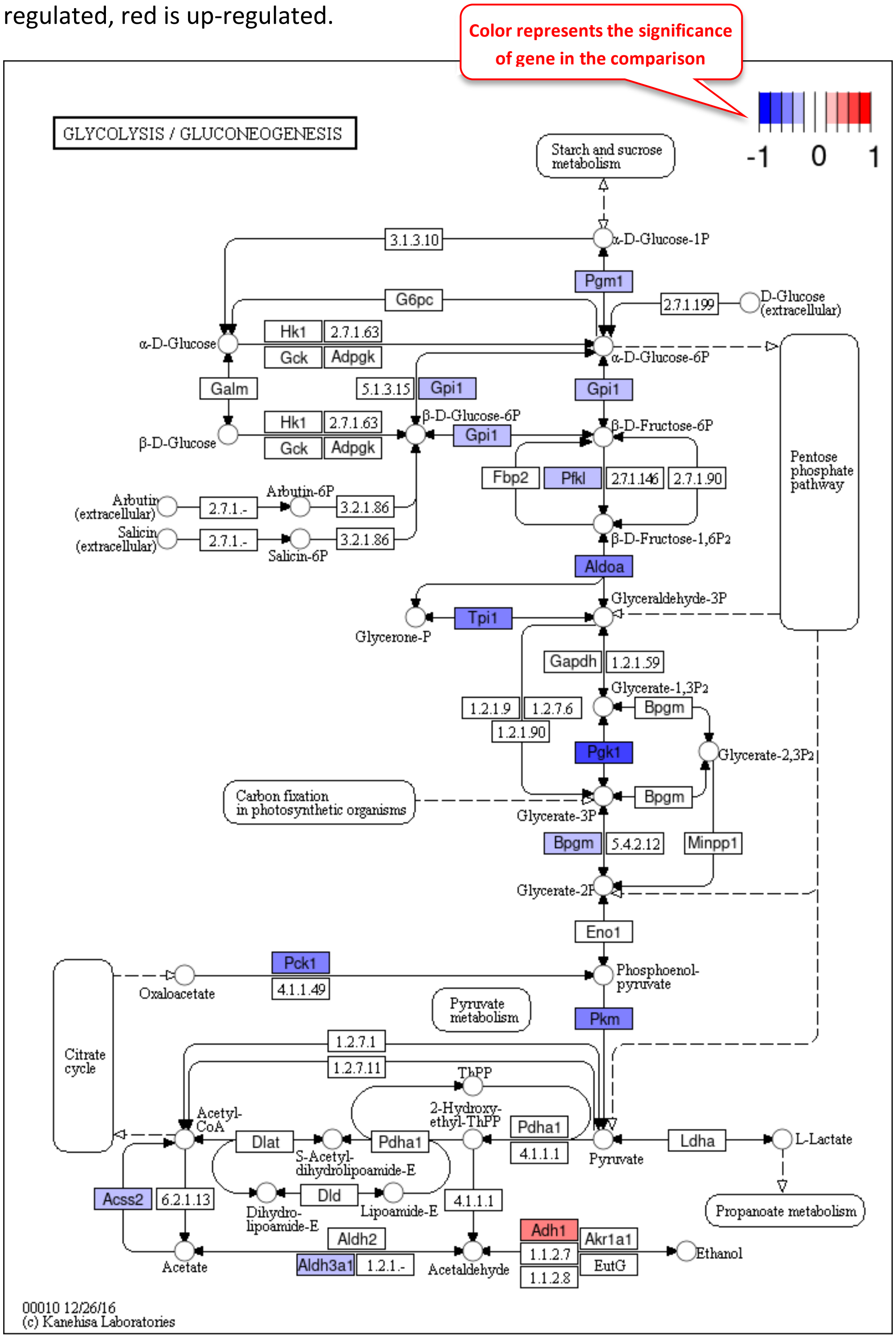

Pathway Plot from Several Comparisons

The user can add multiple comparisons from the pathway plot tool by clicking Add Comparison link. Besides showing log2 Fold Change, the user can also show statistical significance by clicking Enable Second Visualization Columns.

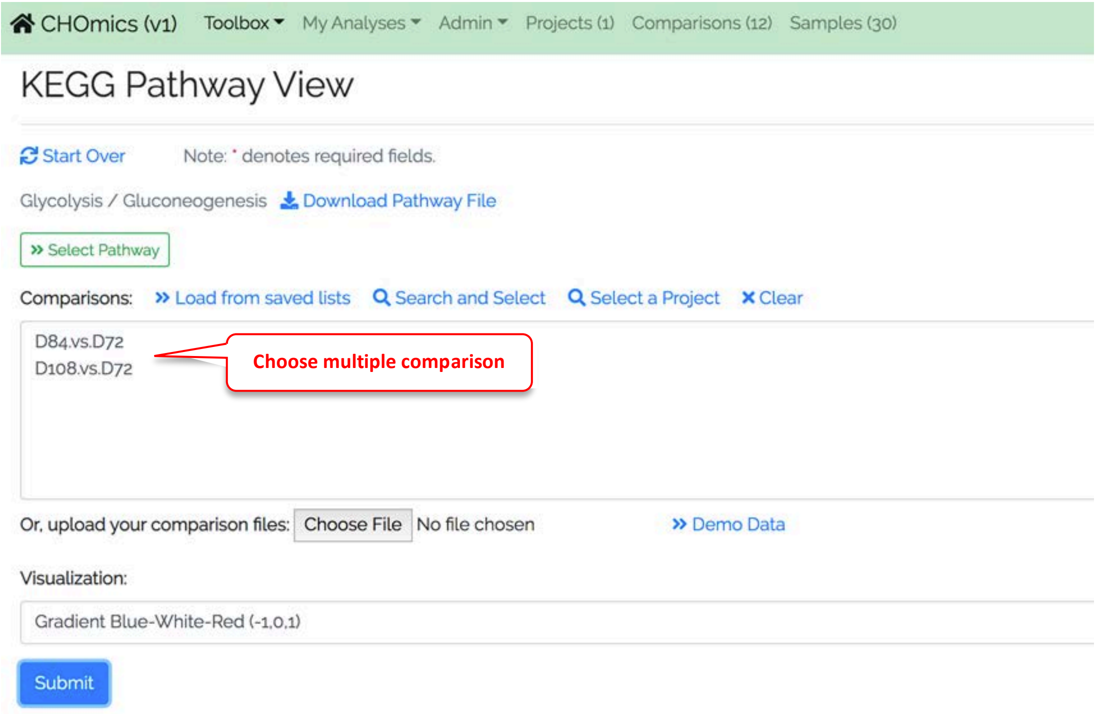

The pathway plot will now have multiple color bars corresponding to the different comparisons.

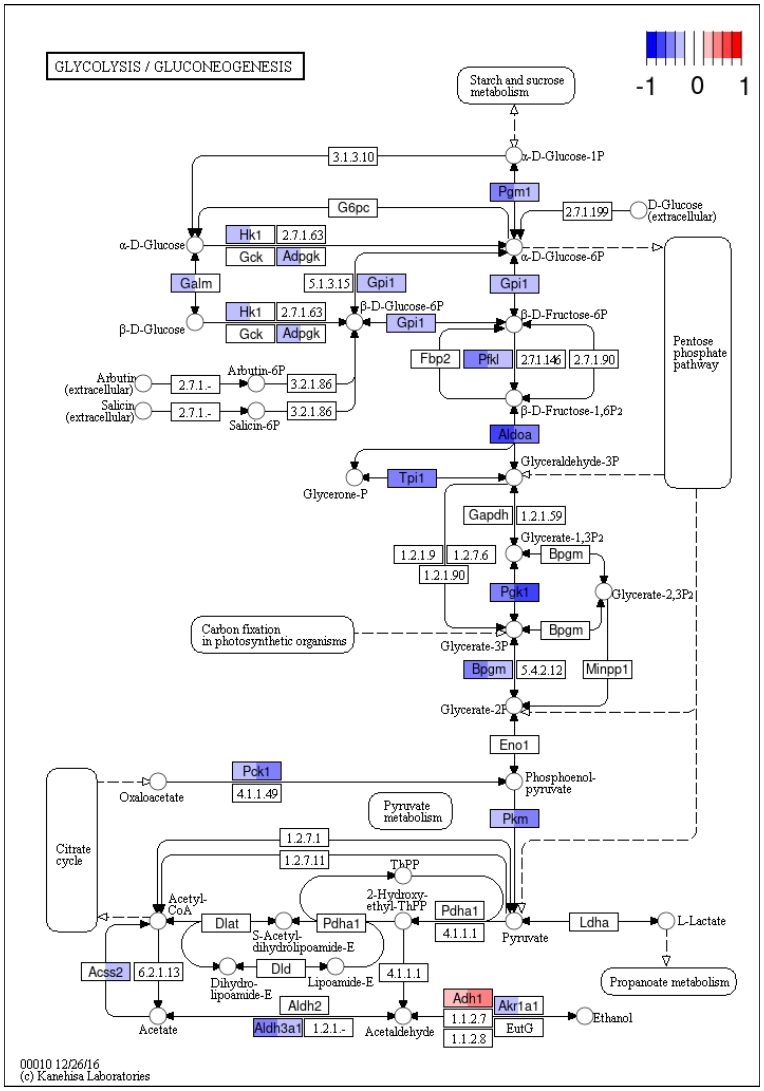

4.3.5 Pathway Heatmap From Comparisons

Users can display the enriched pathways from several related comparisons, and visualize the top enriched pathways across comparisons. Users can mix public data and inhouse comparisons.

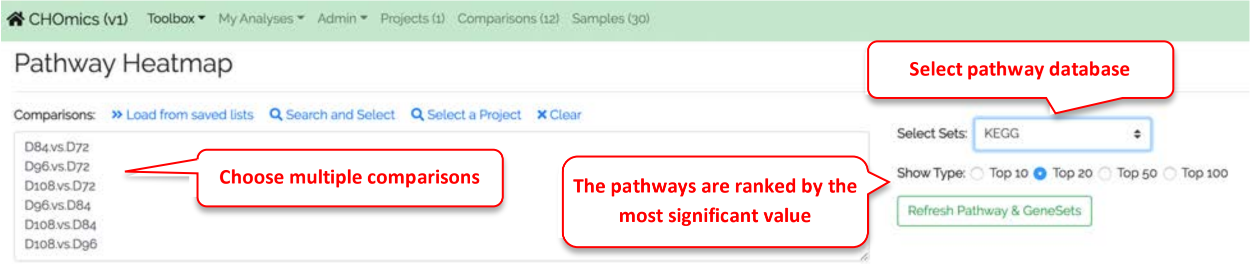

The heatmap shows pathways in rows, comparisons in columns. The statistical significance is color-coded (log P-value, or Z-score). Pathways are sorted by the negative logP values from the highest to the lowest.

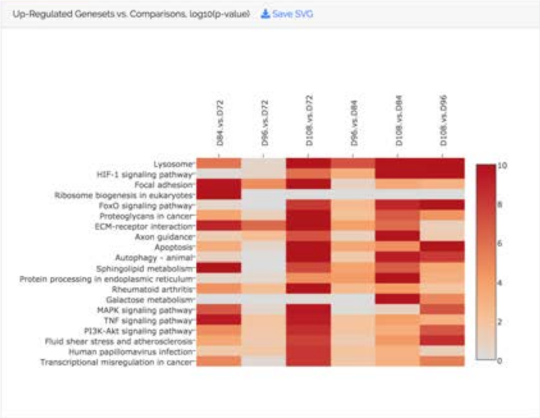

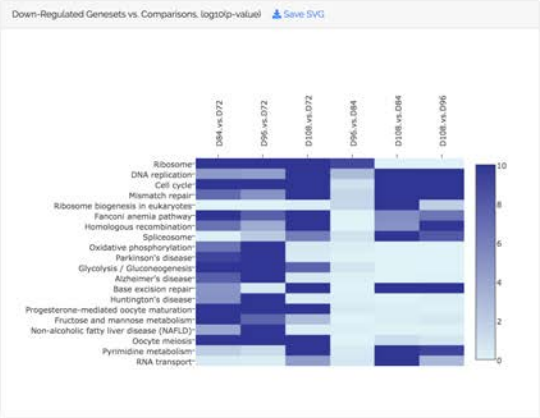

From the pathway heatmap, users can click any data point to view details.

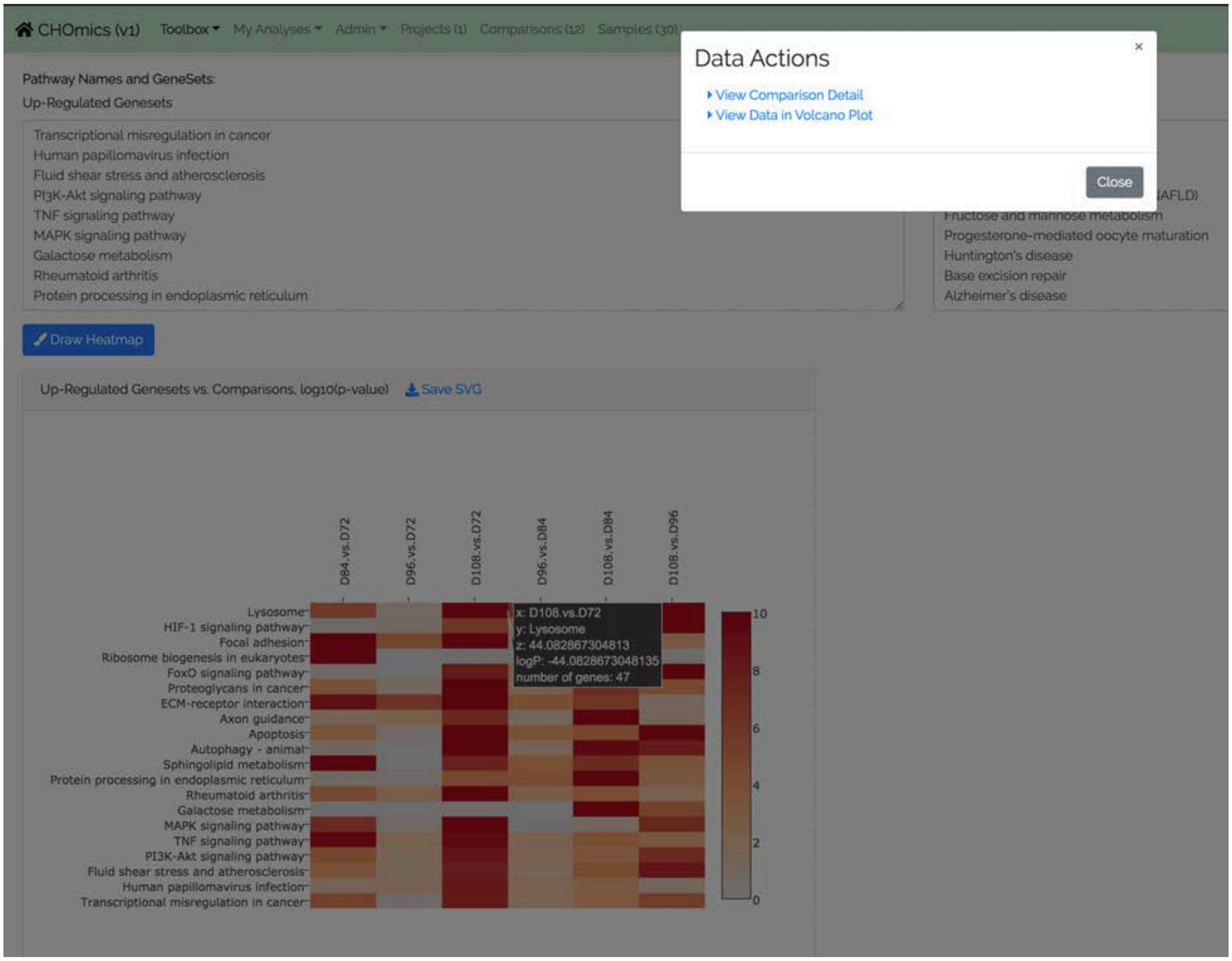

5 Customized analysis pipeline

5.1 Use alternative tool or algorithm

The analysis pipeline is modular, each step can be modified by uses to use an alternative method if desired. The users should be familiar with the Linux bash to run the analysis steps and be familiar with php programming to make modification to the source code.

The full analysis pipeline has four steps, and each step is listed in a bash file in the analysis folder in the system.

- step_0.sh FASTQC of raw data
- step_1.sh Alignment to genome
- step_2.sh Gene count
- step_3.sh DEG detection and functional enrichment

These bash files are created by PHP programs chomics/app/bxgenomics/bxgenomics_exe_analysis.php, when users launch analysis pipeline online in a web browser via chomics/app/bxgenomics/analysis.php. For example, the current pipeline uses subread to perform alignment. If users want to modify the pipeline to change it to use the STAR program for alignment, they need the following steps:

1) Install STAR program on the server, prepare STAR index for the CHO genome.

2) Check the commands in step_1.sh, and change the commands as needed. In this case, the subread command (subjunc step) needs to be replaced by the equivalent STAR command. Since STAR can sort the bam files, the samtools sort step can be omitted. Finally, the STAR output file is named as SampleIDAligned.sortedByCoord.out.bam, an extra step is needed to rename it to SampleID.sorted.bam, so step2.sh can output gene count files with the correct sample names.

3) Edit PHP program chomics/app/bxgenomics/bxgenomics_exe_analysis.php, find the part that generates step_1.sh (The section is marked as “Step 1. Alignment with Subread”), and then make changes accordingly.

4) Test the updated system to make sure it works as expected.
