# Supplementary material for: CHOmics: A web-based tool for multi-omics data analysis and interactive visualization in CHO cell lines
Source: PLoS Comput Biol. 2020 Dec 22;16(12):e1008498. doi: 10.1371/journal.pcbi.1008498 (PMC7790544; doi:10.1371/journal.pcbi.1008498)
Supplement: Attachment — Submitted filename: 2020_Lin_CHOmics_Response_Letter.doc [file pcbi.1008498.s010.doc]

Dear Editor and Reviewers,

We highly appreciate the reviewers and editor’s time and efforts in helping us to improve the manuscript. We have revised our manuscript “CHOmics: a web-based tool for multi-omics data analysis and interactive visualization in CHO cell lines” according to the editor and reviewers’ constructive and valuable comments. In the following, we detail our responses to each comment. Corresponding changes are made in our revised manuscript and GitHub repository.

***Reviewer #1***
**Comments to authors**: *This is an exciting project. I spent my formative years in CHO informatics learning how to do all of the steps of this pipeline individually. This tool will streamline the CHO data analysis process and represents a huge time and monetary saving in terms of a researcher having the lack or expensive hardware to run these processes and not needing to devote countless hours of learning how to do this manually or hiring someone who can. The website is intuitive and walks the user through how every important process works and why it's used. Just this aspect alone is a great learning tool. I was impressed by the ease of use and the actual usefulness of the website.*

*I was able to reproduce the figures and data sets generated in this manuscript by downloading the sample fasta files and manually processing them. This manuscript successfully invents a way to lower the barrier of entry into CHO data analytics and is to be commend.*

*I would have liked to have seen some modularity in the kinds of tools available for use. For instance, some researchers would prefer using subread aligner while others might prefer STAR. But that is a minor quibble. The manuscript does a great job of laying the case for why this tool is important and delivers on the execution of the tool.*

**Response**: Thanks for the valuable comment and verification of results manually. We agree with the reviewer that modular design that allows users to select their preferred tools like different aligners will further enhance the system. This feature can be added in the future update of the platform, nevertheless, we provide an alternative solution that allows users to plug their preferred tools in the platform. This solution will significantly increase the flexibility of the platform for users although it requires programming skills in Linux bash and PHP language. The instruction of how to plug alternative tools in the platform has been outlined in the supplementary tutorial (Section 5). We also provide an example of replacing subread with STAR for alignment in the tutorial.

***Reviewer #2***

**Comments to the Author**: *The authors designed a comprehensive web-based tool for analysis and visualization of CHO omics data. Especially, the tool could be useful in the visualization of different metabolic states of CHO cells, and thus practically relevant for users who are working with various omics data from CHO cell cultures. However, I can’t find any significance or novelty in terms of method development. I think the current work can be submitted to more relevant journals such as Bioinformatics or BMC Bioinformatics after front-end web application setup in the dedicated server so that users can directly do the analysis rather than installing the program. Open source should be available and accessible without signing.*

**Response**: Thank you for the constructive comments. We developed this platform, CHOmics, with strong motivation to provide an interactive and easy-to-use resource instead of a method or algorithm to democratize computational biology analysis in CHO cell line research. We feel that CHOmics fills several gaps in current omics research of CHO cells: 1) to our best knowledge, there is no publicly available software or platform enabling one stop-shop for CHO omics data analysis from raw data to tertiary analysis such as pathway enrichment across data sets while multi-omics analysis has been shown critical in understanding the process of CHO cell lines development. 2) In addition to the built-in comprehensive pipeline for high-throughput sequencing data processing, our tool also stands out from the others by incorporating advanced functionalities to allow users to visualize analysis result systematically and interactively while managing projects in a centralized system. 3) Our open source tool can be installed either on local server or in the cloud that allows for rapid deployment and encourages data sharing and collaboration among the CHO community including pharmaceutical, biotechnology, and academic labs. We provided a demo site ([www.chomics.org](http://www.chomics.org/)) built on a dedicated server but mainly for visualization of the results. If users want to run the data preprocessing pipeline on a large-scale raw dataset, which usually requires significant computing resources, it is recommended to install the platform on a private server using the source code in GitHub. In addition, we provide a publicly available Google Cloud Custom Image that users can deploy by signing up for a Google cloud server and creating an instance based on the image for computational heavy tasks. Based on needs, Google cloud servers can be easily expanded in terms of CPU numbers, memory, disk space, and network types. We have added this in the manuscript at line 293-298.

In addition, we developed this novel platform by integrating many leading algorithms such as multiple methods (PAGE, GSEA and Over-representation based on Hypergeometric Distribution) for pathway enrichment analysis and multiple algorithms for meta-analysis including Rank Product (RP), p-values combined by Fisher method, and p-values combined by maxP. We also developed advanced interactive visualization widgets such as PCA plot, volcano plot, functional overlap of DE analysis results on pathways (e.g, KEGG pathways and Wikipathways).

With theses, we think that CHOmics will have positive impact by providing an end-to-end system to facilitate big data analytics, bioinformatics and systems biology for CHO research that is often lacking in open source bioinformatics tool development but highly desired in industry and industrially oriented academic research on mammalian cell line. Therefore, our work falls in the scope of PLOS Computational Biology journal in publishing ‘software articles describing outstanding open source software of exceptional importance that has been shown to provide new biological insights’.

*I have some minor comments and questions for further improvement:*

*1. There are some abbreviations that are not stated clearly: CHO-S, FDR, TMM. The full names should be provided.*

**Response**: Thanks for the suggestion. We have listed the full name for these abbreviations as:

CHO-S: Chinese Hamster Ovary-Suspension; FDR: False discovery rate; TMM: Trimmed Mean of M-values

We have updated these in the manuscript accordingly.

*2. More details on requirements for installing CHOmics should be provided. Which web browser is most suitable or which programming language is needed to implement CHOmics source code?.*

**Response**: Thanks for the suggestion. The detailed installation steps can be accessed by the link (http://chomics.org/chomics/install.ph). The application is extensively tested with Chrome and Firefox browsers. The main programming languages used in the source code are PHP, R and JavaScript. We have added these details in manuscript at line 290-293.

***Editor***

**Comments to authors:** *We noticed that you used “data not shown”/"unpublished data" in the manuscript, we do not allow these references. The PLOS data access policy requires that all data be either published with the manuscript or made available in a publicly accessible database. Please amend the supplementary material to include the referenced data or remove the references. Please make a note of this change in your response to reviewers.*

*Please ensure that the Author List in your manuscript file matches the Author List in the online submission form, including initials.*

**Response:** We made the correct reference to supplementary tutorial to demonstrate functionality of meta-analysis in manuscript at line 181.

We also modified author list to ensure matches between the manuscript and online submission form.
