# Supplementary material for: CHOmics: A web-based tool for multi-omics data analysis and interactive visualization in CHO cell lines
Source: PLoS Comput Biol. 2020 Dec 22;16(12):e1008498. doi: 10.1371/journal.pcbi.1008498 (PMC7790544; doi:10.1371/journal.pcbi.1008498)
Supplement: Attachment — Submitted filename: 2020_CHOmics_Response_Letter_2ndRevise.docx [file pcbi.1008498.s011.docx]

Dear Editor and Reviewers,

We highly appreciate the reviewers and editor’s time and efforts in helping us to improve the manuscript. We have revised our manuscript “CHOmics: a web-based tool for multi-omics data analysis and interactive visualization in CHO cell lines” according to the editor and reviewers’ comments. In the following, we detail our responses to each comment. Corresponding changes are made in our revised manuscript and demo web site.

***Reviewer #2:*** *Well done and explanation is now clear to me. But, the current results showing the omics data processing and visualization features based on available datasets is not sufficient enough to demonstrate its applicability and usefulness unless the authors like to publish this tool development efforts in Bioinformatics journals. At least one case study should be provided. The authors can use available datasets from any CHO multi-omics publications and show the consistent results and even additional analysis which can be done by CHOmics.*

**Response**: Thanks for the suggestion. In the revised manuscript, we demonstrated the applicability and usefulness of CHOmics in analyzing CHO multi-omics data from an additional public study [1]. In the study, transcriptomics and proteomics data were collected to profile three commonly used parental cell lines (CHO‐K1, CHO‐DXB11, and CHO‐DG44) in suspension cultures. We obtained both transcriptomics data (RMA normalized log2 intensities) and proteomics data (normalized and scaled protein levels) provided by the publication and performed principle component analysis (PCA), differential analysis, and GO enrichment analysis across omics data sets as reported in the publication. We demonstrated that our results (Fig S3-5) are in good agreement with reported in the paper in the above analysis.

In addition, CHOmics offers some extra features in analysis and visualization, such as PCA analysis on the subset of genes (e.g., genes from specific pathway) as shown in Fig S6, investigating the effect size of selected genes across multiple comparisons as shown in the bubble plot (Fig S7), and enrichment analysis on multiple pathway databases (e.g., KEGG, WikiPathways) allowing users to map the differential analysis result from multi-omics data sets on any specific pathway (e.g., Glutathione metabolism) as shown in Fig S8.

We have highlighted the data collection, analysis and results for the additional dataset in the revised manuscript at line 259-276. The output figures by CHOmics from the analysis are listed in the supplementary file Fig S3-8.

1.	Lakshmanan M, Kok YJ, Lee AP, Kyriakopoulos S, Lim HL, Teo G, et al. Multi-omics profiling of CHO parental hosts reveals cell line-specific variations in bioprocessing traits. Biotechnol Bioeng. 2019;116(9):2117-29. Epub 2019/05/09. doi: 10.1002/bit.27014. PubMed PMID: 31066037.
